# Folding without glutamine: Proteome-scale removal of a canonical amino acid

**DOI:** 10.1371/journal.pcbi.1014931

**Published:** 2026-11-03

**Authors:** Alejandro Garciarrubio, Blanca Ramos-Cerrillo, Enrique Morett, Lorenzo Segovia

**Affiliations:** Departamento de Ingeniería Celular y Biocatálisis, Instituto de Biotecnología, Universidad Nacional Autónoma de México, Cuernavaca, Morelos, México; Indonesia International Institute for Life Sciences, INDONESIA

## Abstract

All known life uses a canonical alphabet of 20 amino acids, yet which residues are structurally essential remains untested at proteome scale. Isoleucine was recently eliminated from ribosomal proteins in *Escherichia coli*, establishing that amino acid alphabet reduction is feasible. Here we ask whether an entire amino acid can be eliminated from a proteome, and develop a computational framework to identify the best target. Using two independent approaches — ProteinMPNN structure-based probabilities and multiple sequence alignment conservation — we define “keep-score” as the fraction of positions where the native residue is optimal. Both methods converge on an unexpected candidate: glutamine has the lowest keep-score, with proline and glycine the highest — counter to substitution-matrix intuition. Glutamine’s low keep-score is not explained by a single obvious substitute; glutamate is preferred in only 14% of replacements. Removing this target proteome-wide, we replaced every glutamine across 4,148 proteins with ProteinMPNN-optimal alternatives and modeled the resulting sequences with AlphaFold2. The recoded proteome maintained native-like structures: 85.5% of models exceeded a TMscore of 0.8, indistinguishable from the 85.9% baseline for rebuilt native sequences. Orthogonal validation — ESMFold predictions, Rosetta energy calculations, and conformational ensemble comparisons — revealed no evidence of systematic misfolding attributable to glutamine removal. As a consistency check, a glutamine-free TEM-1 β-lactamase designed with ProteinMPNN conferred ampicillin resistance in *E. coli*, indicating fold preservation can extend to biological function. Evolutionary evidence further supports these findings: 24% of *E. coli* proteins have glutamine-free orthologs in prokaryotic genomes, and glutamine ranks lowest in keep-score in *Saccharomyces cerevisiae*. These results show that an entire amino acid can be removed from a proteome with folds broadly preserved, establishing keep-score as a general framework and glutamine as the primary candidate for recoding. Crucially, this reflects a computational analysis of structural replaceability, not a demonstration of biological dispensability.

## Author summary

All life builds its proteins from the same 20 amino acids. But are all 20 truly necessary, or could some be removed without destroying protein structure? Recent work showed this is possible: a bacterium was engineered to make key proteins without one amino acid. But which one would be easiest to remove from an entire organism? We developed a computational framework to answer this for every protein in *Escherichia coli*. Using an AI model that predicts which amino acids fit a protein’s three-dimensional shape, together with evolutionary conservation data, we ranked all 20 by how often each is the best choice at its position. Both independently singled out glutamine as the most readily removable and the best single target — a surprise, because no single alternative dominates its replacement; instead, many amino acids can fill in depending on structural context. Rebuilding the proteome without glutamine yielded predicted structures nearly identical to the originals. A computationally designed glutamine-free enzyme, tested against its native counterpart, retained comparable activity, showing that fold preservation can translate to biological function. Our framework applies to any organism, offering a rational strategy for choosing which amino acid to target when building cells with simplified genetic codes.

## Introduction

All known life encodes proteins using the same 20 standard amino acids, a universality that has persisted across billions of years of evolution [1]. Whether this conservation reflects strict biochemical necessity or evolutionary contingency has remained an open question [2]. Some organisms approach extreme amino acid depletion — AT-rich insect endosymbionts such as Carsonella exhibit severe compositional biases driven by genomic GC content [3,4] — but no free-living organism had been shown to completely eliminate any canonical amino acid from its proteome. Recently, Liu et al. [5] eliminated all isoleucine residues from the ribosomal proteins of *Escherichia coli* using generative AI-guided design, a first step toward a 19-amino-acid organism. Their Ec19 strain, carrying 21 Ile-free ribosomal subunits at a native genomic locus, remained viable and genomically stable for over 450 generations, establishing that even an essential, universally conserved machine can be rebuilt without one canonical amino acid. The feasibility of drastic alphabet reduction had earlier been demonstrated at the single-protein level: Shibue et al. [6] systematically eliminated amino acids from an ancestral nucleoside kinase and obtained soluble, stable, catalytically active variants composed of only 13 amino acid types, most of which coincide with prebiotically plausible residues. Comparable reductions have been achieved in other systems: Yagi et al. [7] reconstructed the core fold of RNA polymerase from only seven amino acid types, and Makarov et al. [8] engineered a catalytically active dephospho-CoA kinase devoid of Phe, Tyr, and Trp; earlier, Longo et al. [9] designed a stable, foldable protein from a prebiotically plausible alphabet. Together, these results transform the question from whether amino acid reduction is possible to which amino acid is most dispensable and why. Answering this question has fundamental implications for understanding protein architecture and practical consequences for engineering organisms with deliberately reduced genetic codes, incorporating non-canonical amino acids, or implementing biocontainment strategies [2,10,11].

Liu et al. [5] selected isoleucine on the basis of evolutionary conservation: across multiple sequence alignments of *E. coli* proteins, Ile is among the least conserved residues and is frequently substituted by the biochemically similar valine. This choice was reinforced by practical considerations — branched-chain amino acid redundancy, the higher biosynthetic cost of Ile relative to Val, and the availability of a clean auxotrophic knockout. Yet even with this favorable target, simple Ile-to-Val substitution succeeded in only 43% of essential proteins tested; the remaining cases required multiple generative models, compensatory mutations, and iterative experimental cycles involving hundreds of design variants. This difficulty raises a question their work did not address: is isoleucine actually the easiest amino acid to remove from the proteome, or would a different choice have been more tractable?

Classical intuition about amino acid replaceability derives from substitution matrices such as PAM and BLOSUM, which capture evolutionary exchange patterns across protein families [12,13]. These matrices suggest that hydrophobic residues — particularly isoleucine, which frequently exchanges with leucine and valine — would be among the easiest to eliminate [14,15]. Similarly, studies on reduced amino acid alphabets have sought to identify groups of residues that can be merged without losing structural information. Murphy et al. [16] developed a hierarchy of simplified alphabets optimized for fold recognition, and Takabatake et al. [17] extended this approach for large-scale homology searches. In both cases, the first merger is Ile with Val — consistent with their well-known biochemical interchangeability — and the second is glutamine with glutamate, suggesting that these two residues carry partially redundant structural information. An information-theoretic analysis of long-range contact interactions reached a compatible conclusion: Solis [18] found that reduced alphabets of fewer than 10 amino acids preserve virtually all the structural information encoded in native contacts, and that optimal clusters derived from contact potentials converge with those derived from physicochemical intuition and sequence-alignment-based approaches. However, substitution matrices and reduced alphabets reflect the composite pressures of evolution — functional constraints, expression-linked selection, and lineage-specific biases — rather than purely structural considerations [19–21]. What evolution has tolerated in pairwise exchanges may differ fundamentally from what protein architecture actually requires. Testing this distinction demands a different approach: systematically evaluating each amino acid’s contribution to fold stability across an entire proteome and asking which one is least required.

Recent advances in artificial intelligence make such an assessment computationally tractable. AlphaFold2 predicts protein structures with near-experimental accuracy [22,23], while ProteinMPNN performs inverse folding — designing sequences likely to adopt a target three-dimensional structure [24]. Crucially, ProteinMPNN evaluates each position independently, assigning probabilities to all 20 amino acids based on local and global structural context. This means that when evaluating the removal of an amino acid, each occurrence can be replaced with the optimal alternative at that specific position, rather than a single uniform substitution as in simple recoding schemes. That inverse-folding models can navigate severely restricted sequence spaces has been confirmed experimentally: Giacobelli et al. [25] used ProteinMPNN and RFdiffusion to design *de novo* protein folds from a 10-amino-acid alphabet lacking all basic and aromatic residues, obtaining structures that adopted their intended topologies and resisted thermal and chemical denaturation. Combined with AlphaFold2’s ability for rapid structural validation, these tools enable proteome-scale redesign experiments that were previously impossible [26,27]. Importantly, structure prediction models have also been shown to interpret reduced-alphabet sequences: Ieremie et al. [28] demonstrated that ESMFold can predict native-like structures from protein sequences translated into reduced alphabets of 12 or fewer amino acid types, supporting the validity of using deep-learning structure predictors to evaluate recoded sequences. Both ProteinMPNN and AlphaFold2 proved essential in the Liu et al. [5] study for generating functional Ile-free ribosomal proteins; here, we apply the same tools not to solve individual design problems but to answer the prior question of target selection.

We asked whether an entire canonical amino acid could be eliminated from a proteome, and applied this approach to *E. coli*, developing quantitative metrics to identify the most promising target. We define “keep-score” as the fraction of positions where the native residue is deemed optimal — either the highest probability under ProteinMPNN or the most conserved in multiple sequence alignments — and “keep-propensity” as the margin by which the native residue is favored over its best alternative. Two independent methods converge on the same unexpected candidate: ProteinMPNN, a structure-based inverse-folding model, and MSA conservation, reflecting billions of years of evolutionary selection, both assign the lowest keep-score to glutamine — not isoleucine — with proline and glycine highest. This ranking contradicts predictions from substitution matrices and reveals a disconnect between evolutionary exchangeability and structural constraint. Glutamine’s replaceability is not explained by the availability of an obvious substitute: although reduced-alphabet analyses group it with glutamate, no single residue dominates its replacements — unlike isoleucine, which has an obvious substitute in valine yet ranks among the more constrained residues by keep-score. Replaceability, it appears, is not determined by similarity to a single alternative but reflects the degree to which a residue is structurally unconstrained — rarely imposing strict geometric requirements on the folds it occupies.

Removing this candidate proteome-wide, we replaced every glutamine in 4,148 *E. coli* proteins with the optimal ProteinMPNN-predicted alternative and modeled the resulting sequences with AlphaFold2. The glutamine-free proteome maintained native-like structures indistinguishable from the rebuilt-native baseline within the resolution of the prediction method, and multiple complementary approaches — local confidence, Rosetta energetics, an orthogonal structure predictor (ESMFold), flexible alignments, and conformational-ensemble comparisons — revealed no evidence of systematic misfolding attributable to glutamine removal. Applying the same analysis to isoleucine — the residue eliminated by Liu et al. — confirmed that it is more disruptive to remove than glutamine, showing that the framework discriminates among amino acids rather than tolerating any substitution uniformly. As a consistency check, we engineered a glutamine-free TEM-1 β-lactamase that confers ampicillin resistance in *E. coli*, indicating that fold preservation can extend to biological function. Evolutionary evidence supports these findings: at least 24% of *E. coli* proteins have glutamine-free orthologs in prokaryotic genomes, and keep-scores computed independently for *Saccharomyces cerevisiae* rank glutamine lowest and correlate strongly with those of *E. coli*, indicating that the ranking reflects general features of protein architecture rather than a peculiarity of *E. coli*. We emphasize that this study is a computational analysis of structural replaceability, not a demonstration of biological dispensability — a protein that folds correctly without glutamine may still lack catalytic activity or binding specificity if glutamine participates directly in its mechanism [29,30]. Nevertheless, fold preservation is a necessary first step: a redesigned protein that cannot properly fold certainly will not function. That an entire amino acid can be removed from a proteome with folds broadly preserved raises a new question: why has evolution universally retained an amino acid that protein architecture, as assessed by current computational methods, does not appear to strictly require?

## Results

We asked whether a bacterial proteome could eliminate a single canonical amino acid without compromising its ability to fold. Amino acid abundances vary widely across prokaryotes (S1 Fig); *E. coli* closely matches the prokaryotic median, making it a suitable model for this study. In *E. coli*, glutamine is the sixth least abundant amino acid—relatively rare, but not exceptionally so. To identify which residues are structurally dispensable, we asked how often each native amino acid is the optimal choice at its position. We define “**keep-score**” as the fraction of positions where the native residue is deemed best: either the highest probability under ProteinMPNN or the most frequent in the MSA column (Fig 1). Both criteria agree that Pro and Gly are the least removable, consistent with their unique roles in defining secondary structure. Conversely, both methods identify Gln as the most removable amino acid. Under ProteinMPNN, the keep-score for Gln is only 0.13, meaning this tool would replace 87% of all glutamines in the *E. coli* proteome. The convergence of two independent methods—one trained on structure, the other reflecting evolutionary selection—argues that glutamine’s dispensability is not an algorithmic artifact.

**Fig 1:**
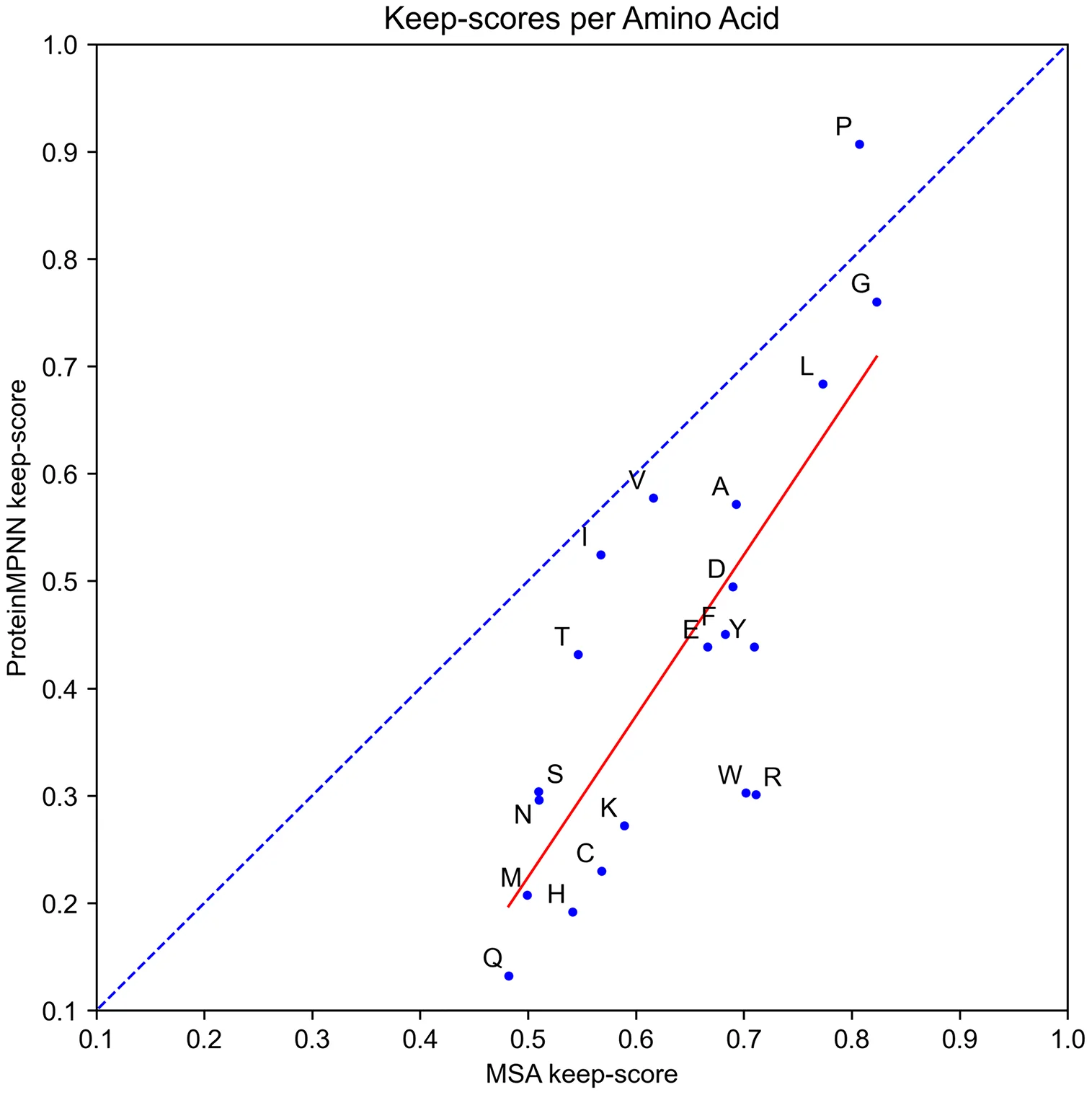
Keep-scores by amino acid. Keep-score is the fraction of positions in the *E. coli* proteome (4,363 proteins) where the native amino acid is deemed optimal — either the highest-probability residue under ProteinMPNN (y-axis) or the most conserved in multiple sequence alignments of eubacterial homologues (x-axis). Both methods identify glutamine (Q) as the most replaceable amino acid and proline (P) and glycine (G) as the least replaceable. Dashed line: y = x; solid red line: linear regression (y = 1.50 x − 0.53; Pearson r = 0.78, P = 5.2 × 10^−5^). Single-letter amino acid codes are shown next to each point.

Keep-score, however, reflects only a binary retain-or-replace call at each position: it reveals how often a residue is retained but not by how much it is favored or disfavored. To address this, we defined “keep-propensity” as the difference between the probability (or frequency) of the native amino acid and that of its best alternative, ranging from −1.0 (strong preference for replacement) to +1.0 (strong preference for retention). Under ProteinMPNN, only Ala, Gly, Leu, and Pro show distributions with prominent peaks near +1.0, reflecting their irreplaceable structural roles (S2A Fig). MSA-based distributions show retention peaks for all amino acids, but Gln has one of the smallest (S2B Fig). Thus, glutamine is not only replaced most often—it is also replaced with the least resistance.

What would the *E. coli* proteome look like under optimal recoding? When we replaced every residue with the top choice from ProteinMPNN or the MSA consensus, the largest shift was for glutamine: its abundance would decrease approximately threefold under ProteinMPNN, from 3.8% to 1.2% (S3 Fig). MSA-guided recoding predicted more modest changes, with cysteine showing the largest relative shift. Examining replacement preferences reveals a counterintuitive pattern (S4 Fig). Isoleucine shows the strongest single replacement bias—over 30% of substituted isoleucines become valine—yet Ile is not among the most replaceable amino acids by keep-score, contradicting predictions from classical substitution matrices. Conversely, glutamine lacks a dominant substitute: glutamate is favored in only ~14% of cases. Thus, the availability of a physicochemically similar alternative does not determine dispensability. Glutamine’s replaceability appears to reflect a lack of strict positional constraints rather than the presence of an obvious substitute.

The preceding analyses identify glutamine as structurally dispensable based on sequence-level metrics, but do the resulting proteins actually fold? To answer this, we used AlphaFold2 (AF2) to model mutant sequences and compared them to reference structures from AlphaFoldDB. First, we established a baseline by modeling the native *E. coli* sequences themselves (Fig 2A). These “rebuilt” models are necessary because AF2 does not always reproduce the same structure for flexible or disordered proteins. We found that 86% of rebuilt models have TMscore above 0.8 against their AlphaFoldDB references—a threshold we adopt throughout as indicating near-native agreement. Proteins falling below this threshold are not misfolded; their low TMscores correlate strongly with high predicted aligned error (PAE), indicating intrinsic flexibility (Fig 2B). We define each protein’s rebuilt TMscore as its “ease-score,” which quantifies how reliably AF2 reproduces that structure and allows us to control for flexibility in subsequent comparisons.

**Fig 2:**
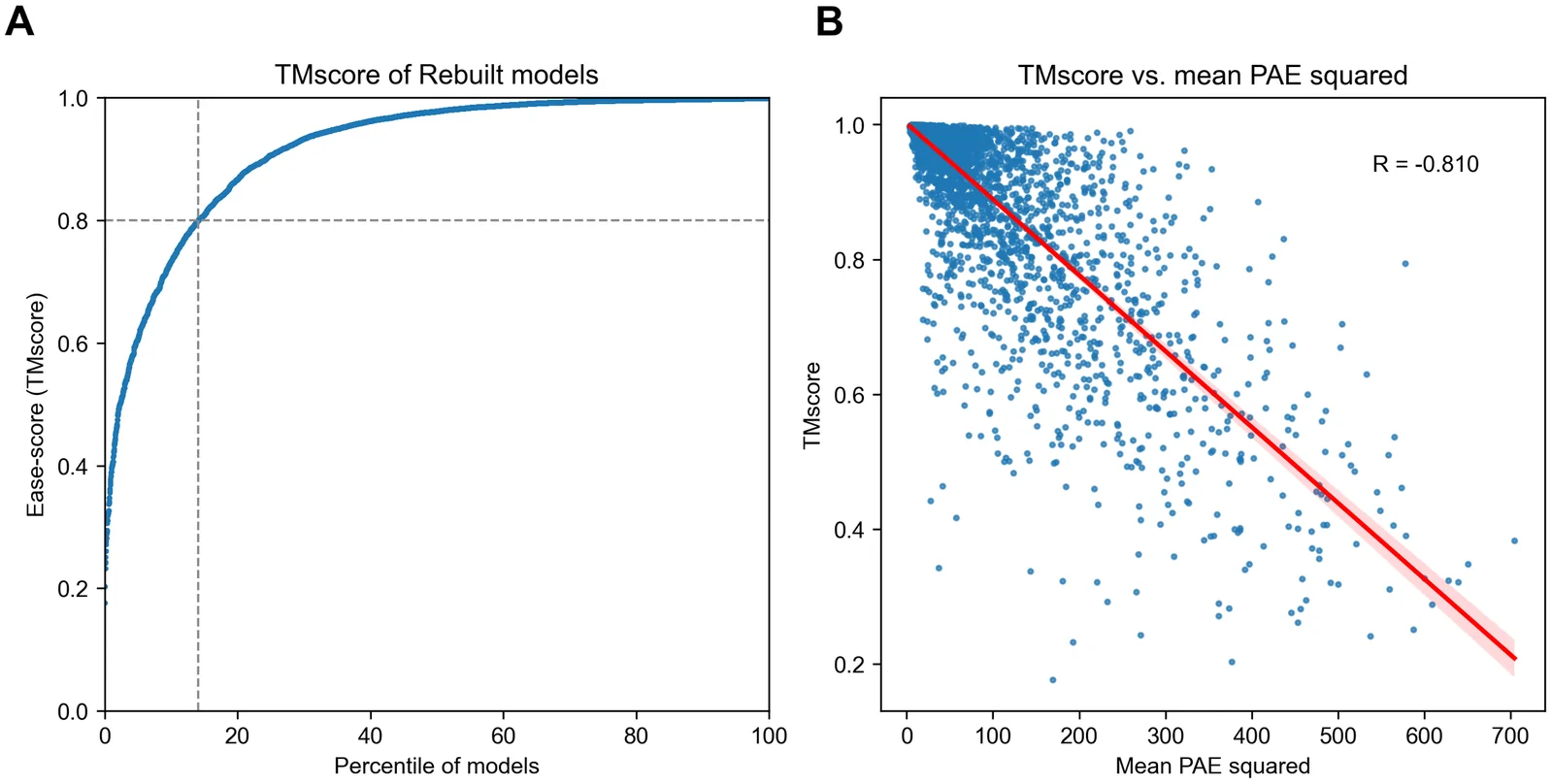
Baseline structural accuracy of Rebuilt models. Native *E. coli* sequences (4,148 proteins, 50–890 aa) were remodeled with AlphaFold2 (Rebuilt models) and compared to AlphaFoldDB reference structures using TMscore. **(A)** Cumulative distribution of TMscore. Each protein’s Rebuilt TMscore defines its “ease-score,” a baseline that quantifies how reliably AlphaFold2 reproduces that structure and controls for intrinsic flexibility in subsequent comparisons. Horizontal dashed line: TMscore = 0.8, the threshold adopted throughout this study for near-native structural agreement; 86% of proteins exceed this threshold. **(B)** TMscore vs. squared mean predicted aligned error (PAE). Proteins with low TMscores have high PAE values (r = −0.81), indicating that low scores reflect intrinsic disorder or flexibility rather than prediction failure.

We next asked whether keep-scores predict the structural impact of removing an amino acid. For adequate statistical power we assembled 300 proteins with very high ease-scores (the mut_each_300_msa set; Methods), and for each of the 20 amino acids in turn replaced every occurrence with the most frequent alternative in its MSA column, modeling the resulting sequences with AlphaFold2. We later use a second, independently designed set (mut_each_200; below) as a control against circularity. We quantified impact as the RMSD to the AlphaFoldDB reference, averaged over the five AF2 models per sequence; for these high-ease proteins TMscore saturates near 1.0 and cannot resolve small differences between amino acids, whereas RMSD remains sensitive. MSA-consensus replacements were used rather than ProteinMPNN-optimal ones, which minimize structural perturbation for every amino acid and thereby compress the differences we sought to measure.

Ranking the amino acids by median removal RMSD reproduces the keep-score order: of 134 pairwise differences significant after Hochberg correction, 126 agree with the keep-score ranking and only 8 oppose it (Fig 3). Removing glycine, leucine, alanine, valine, or proline—all high-keep residues—caused the largest disruptions, consistent with their roles in backbone geometry and packing. Removing glutamine was among the least disruptive of all: it was significantly less disruptive than eleven higher-keep residues (R, S, T, F, D, I, A, V, L, G, and P) and never significantly more disruptive than any residue, though it was statistically indistinguishable from the other low-keep residues (H, M, C, K, N, W, E, and Y). Glutamine is therefore not uniquely dispensable by this measure, but it lies firmly among the least impactful residues and, having the lowest keep-score, is the best single target identifiable a priori.

**Fig 3:**
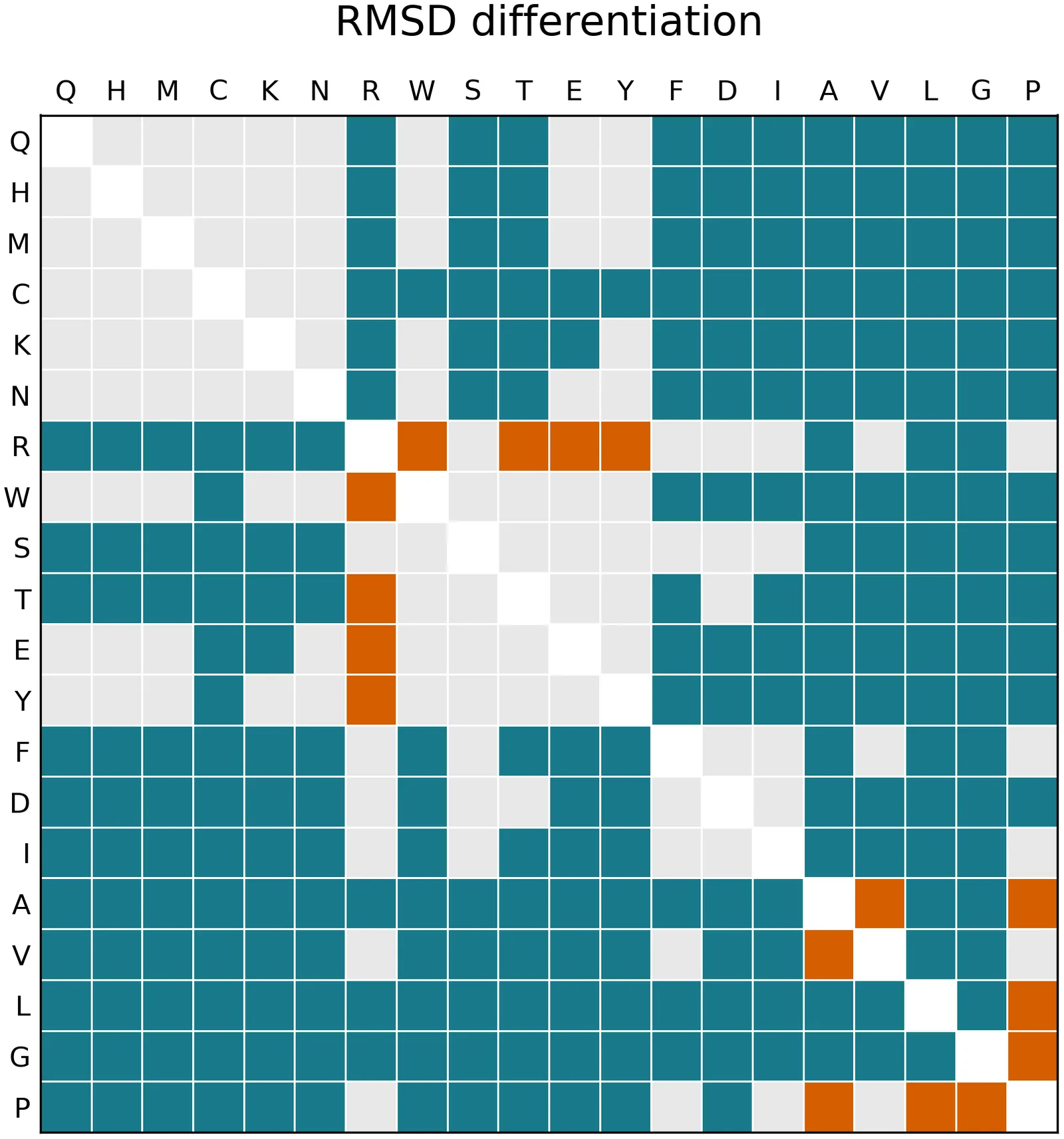
RMSD differentiation among amino-acid variants. Pairwise RMSD differentiation between the 20 amino-acid removal variants across the 300 matched proteins in the `mut_each_300_msa` set. Amino acids are ordered by ascending ProteinMPNN keep score (mpnn_keep). Each off-diagonal cell represents a two-sided paired Wilcoxon signed-rank test comparing RMSD values for the corresponding amino-acid pair across proteins. P-values were Hochberg-corrected across all 190 amino-acid pairs; colored cells meet the adjusted P < 0.05 threshold. Teal indicates a significant difference that agrees with the expected ordering (lower keep score associated with lower RMSD); orange indicates a significant difference in the opposite direction; gray indicates a non-significant comparison; and white diagonal cells are self-comparisons and were not tested. Of the 134 Hochberg-significant pairs, 126 agree with the mpnn_keep ordering and 8 oppose it.

This ranking is not an artifact of using the same tool to design and to score. We repeated the analysis on an independent set of 200 proteins (the mut_each_200 set; Methods) in which each amino acid was removed by ProteinMPNN-optimal replacement rather than MSA consensus, and again ranked the residues by median removal RMSD. The two sets are designed by opposite criteria, yet in each the RMSD ranking agrees best with the *other* method’s keep-score: the MSA-designed mut_each_300_msa ranking correlates most strongly with the ProteinMPNN keep-score (Spearman ρ = 0.88), and the ProteinMPNN-designed mut_each_200 ranking with the MSA keep-score (ρ = 0.71; S5 Fig). Were the agreement between keep-score and removal impact merely a consequence of scoring sequences with the tool that designed them, each set would track its own design criterion rather than the other’s. That the association crosses methods indicates that keep-score captures a property of each position that is visible to structure-based design and to evolutionary conservation alike, independent of which one generated the sequence.

Before removing glutamine specifically, we tested whether proteome-wide optimal recoding preserves structure. We rebuilt every *E. coli* protein using either the highest-probability amino acid at each position (ProteinMPNN-ideal) or the MSA consensus, then modeled both with AF2. Both strategies preserved native folds, with median TMscores of 0.96 (ProteinMPNN) and 0.95 (MSA) against AlphaFoldDB references (S6 Fig). Notably, ProteinMPNN achieved this at much lower sequence identity: 0.46 versus 0.66 for MSA consensus (S7 Fig). This confirms ProteinMPNN’s ability to design highly divergent sequences that remain structurally compatible with the native fold—a prerequisite for the proteome-wide glutamine removal that follows.

We now arrive at the central experiment: can the *E. coli* proteome fold without glutamine? We replaced every glutamine with the highest-probability alternative under ProteinMPNN (all 4,148 proteins) or with the most frequent non-glutamine residue in the conservation MSA (the 3,960 proteins with an available MSA), then built AF2 models for both (Fig 4). The results are striking. ProteinMPNN-designed sequences without glutamine reached a mean TMscore of 0.914, virtually identical to 0.917 for the rebuilt native models; 85.5% exceeded the 0.8 threshold, compared with 85.9% for natives. Grouped by ease-score, the No-Gln models matched the rebuilt baseline across the entire range—from the most rigid to the most flexible proteins. MSA-based designs performed worse, with a mean TMscore of 0.895 and only 78% above threshold. In short, ProteinMPNN can remove every glutamine from a bacterial proteome matching the same structural accuracy of native sequences.

**Fig 4:**
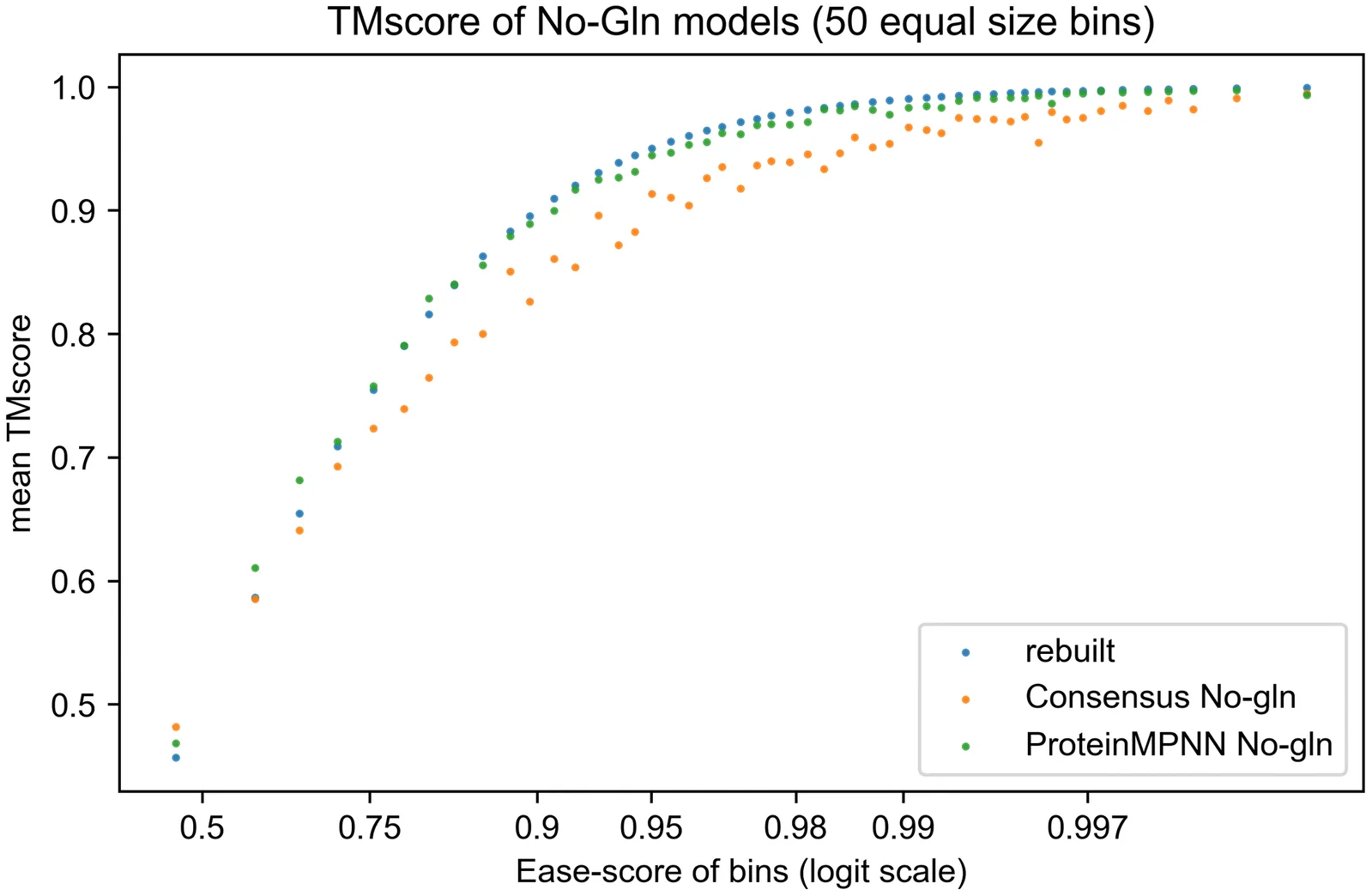
Structural accuracy of glutamine-free proteomes. Every glutamine in 4,148 *E. coli* proteins was replaced with the highest-probability non-glutamine residue from ProteinMPNN (green; 4,148 proteins) or the most frequent non-glutamine residue in the MSA (orange; 3,960 proteins with an available MSA), and the resulting sequences were modeled with AlphaFold2. Rebuilt native models (blue) serve as the baseline. Mean TMscore (vs. AlphaFoldDB references) is plotted per ease-score bin (50 equal-sized bins on a logit-transformed x-axis; each protein keeps its bin across strategies, so the three series are compared on the same proteins — except the MSA series, which lacks a few proteins in some bins). ProteinMPNN-designed glutamine-free sequences (mean TMscore 0.914; 85.5% above 0.8) matched the Rebuilt baseline (0.917; 85.9%) across the entire flexibility range. MSA-based designs performed worse (mean TMscore 0.895; 78% above 0.8).

We validated the structural integrity of the No-Gln proteome through three complementary approaches, each probing a different potential failure mode (Fig 5). First, we asked whether AF2’s confidence changed near substitution sites. For each replaced glutamine, we computed the mean pLDDT of residues within 7 Å in both No-Gln and Rebuilt models. The correlation is near-perfect (slope = 0.954, R^2^ = 0.95; Fig 5A), indicating that AF2 predicts local geometry with equal certainty before and after glutamine removal. Second, we evaluated energetic stability using Rosetta’s all-atom energy function. After constrained relaxation, No-Gln and Rebuilt energies correlated almost perfectly (slope = 0.995, R^2^ = 0.985), with No-Gln models showing marginal mean stabilization (−4.2 REU; Fig 5B). Third, we tested whether an entirely different folding method would reach the same conclusion. ESMFold, a protein language model that does not use explicit sequence alignments, produced TMscores for No-Gln models that were indistinguishable from Rebuilt (slope = 0.971, R^2^ = 0.955; Fig 5C). Across all three metrics—local confidence, energetic stability, and an orthogonal folding algorithm—the glutamine-free proteome is structurally indistinguishable from the native within the resolution of these methods.

**Fig 5:**
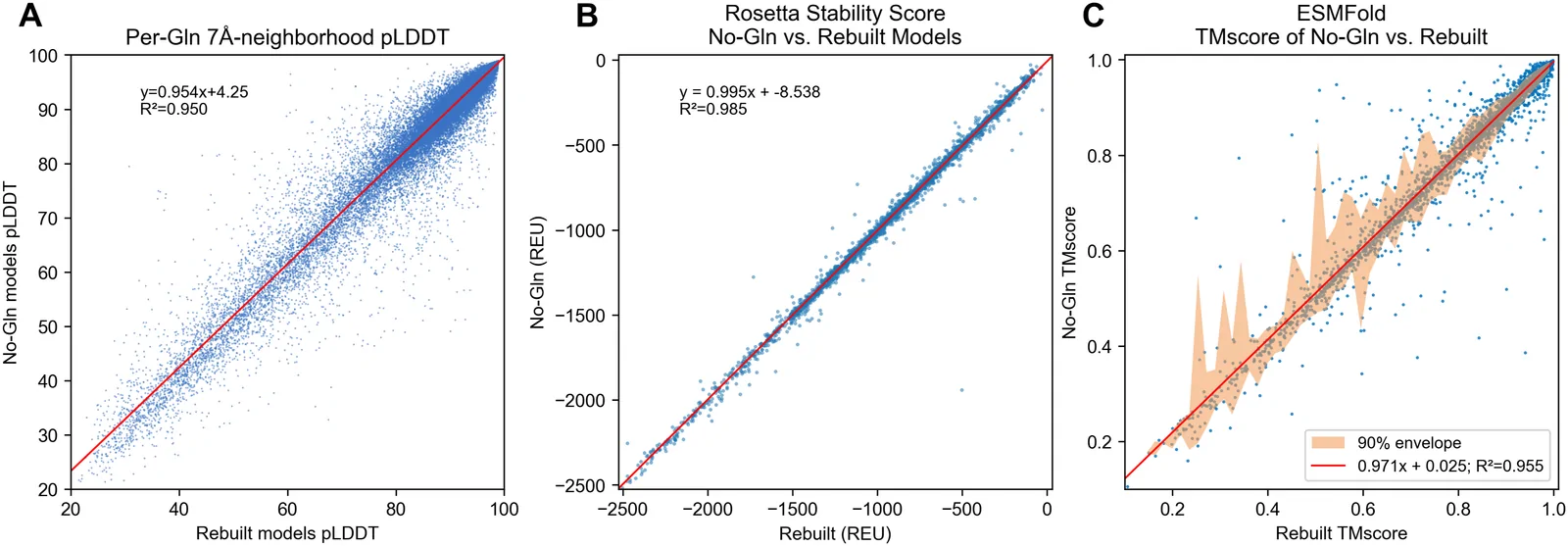
Validation of glutamine-free (No-Gln) structural integrity by three complementary approaches. All comparisons are between ProteinMPNN No-Gln and Rebuilt models for 4,148 *E. coli* proteins. **(A)** Local structural confidence. For each replaced glutamine, the mean pLDDT of all residues within 7 Å (Cα–Cα distance) was computed in both Rebuilt and No-Gln models. The near-perfect correlation (slope = 0.954, R² = 0.95) indicates that AlphaFold2 predicts local geometry with equal confidence before and after glutamine removal. **(B)** Energetic stability. Rosetta all-atom energy (REU) after constrained relaxation (Cα harmonic restraints, FastRelax with ref2015), shown for proteins with ease-score > 0.8. No-Gln and Rebuilt energies correlate almost perfectly (slope = 0.995, R² = 0.985), with No-Gln models showing marginal mean stabilization (−4.2 REU). **(C)** Orthogonal structure prediction. ESMFold, a protein language model independent of explicit sequence alignments, was used to model both Rebuilt and No-Gln sequences. TMscores (vs. AlphaFoldDB references) are indistinguishable (slope = 0.971, R² = 0.955). Orange shading: 5th–95th percentile envelope computed over 50 equal-width segments.

About 14% of No-Gln models fall below TMscore 0.8—do these represent misfolded structures? Three analyses indicate they do not (Fig 6). Alignment with FoldMason, which scores local structural similarity independently of global superposition, classified 99.2% of ProteinMPNN No-Gln models as perfect (mean LDDT = 1.0) and 99.7% as good (mean LDDT > 0.9), virtually identical to Rebuilt natives (Fig 6A). Flexible alignment—which allows domain rearrangements while preserving local structure—rescued most low-scoring proteins (Figs 6B and S8). We analyzed 548 proteins whose No-Gln models had rigid TMscore below 0.8; of these, 60 had Rebuilt TMscore above the threshold, indicating that glutamine removal lowered their scores. Under flexible alignment, 333 No-Gln models (61%) and 341 Rebuilt models (62%) crossed the 0.8 threshold, showing equivalent rescue rates regardless of starting point. Finally, we asked whether low-scoring No-Gln models were statistically distinguishable from native conformational variation. Using BioEmu, we generated 50 conformational samples from each native sequence, obtained their TMscores to the Reference models, and compared these distributions to the No-Gln TMscores (Figs 6C and S9). Of 497 proteins tested, only nine scored significantly lower than the native ensemble (P < 0.05), reduced to two after correction for multiple testing (q ≤ 0.05). Visual inspection of these two proteins (P37619 and P64439) confirmed that all three models—AlphaFoldDB reference, Rebuilt, and No-Gln—share the same fold (S10 Fig). In summary, we found no evidence that removing glutamine causes misfolding in any *E. coli* protein (S11 Fig).

**Fig 6:**
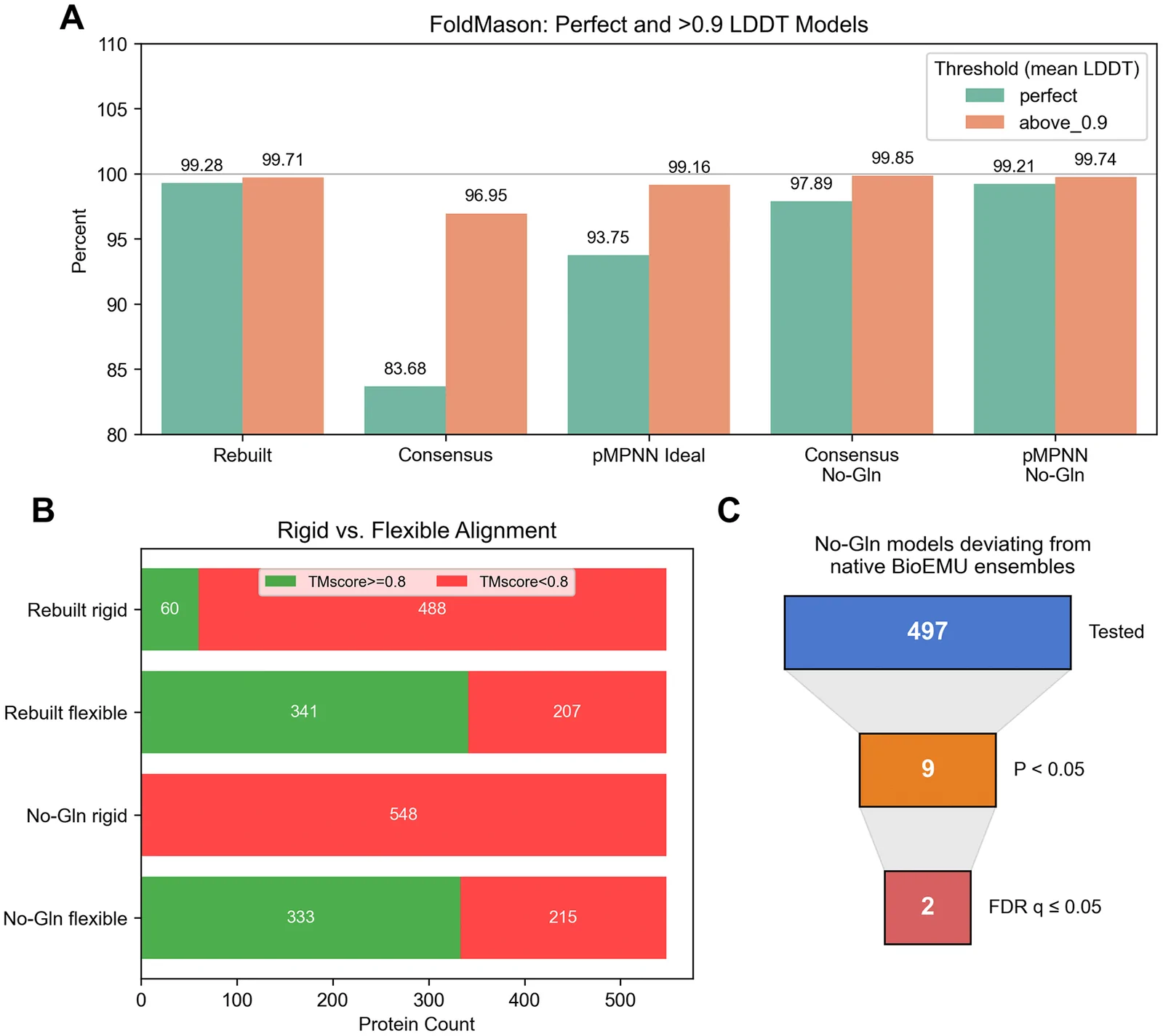
Low-TMscore No-Gln models are not misfolded. Three analyses addressing the ~ 14% of ProteinMPNN No-Gln models that fall below the TMscore 0.8 threshold. **(A)** FoldMason local structural alignment. Percentage of models classified as “perfect” (mean LDDT = 1.0) or “good” (mean LDDT > 0.9) for each recoding strategy and the Rebuilt baseline. FoldMason scores local structural similarity independently of global superposition; 99.2% of ProteinMPNN No-Gln models are classified as perfect, virtually identical to Rebuilt natives. **(B)** Rigid vs. flexible alignment for 548 proteins whose No-Gln models had rigid TMscore < 0.8. Of these, 60 had Rebuilt rigid TMscore ≥ 0.8. With flexible alignment, which allows relative domain movements, 333 No-Gln models (61%) and 341 Rebuilt models (62%) had a TMscore ≥ 0.8. **(C)** Comparison with native conformational variation. BioEmu was used to generate 50 conformational samples from each native sequence; each protein’s single No-Gln TMscore was tested as a one-sided outlier against its native ensemble (predictive t-test, n − 1 df; No-Gln below the ensemble mean). Of 497 proteins tested, nine scored significantly lower than their native ensemble (P < 0.05), reduced to two after Benjamini–Hochberg correction (FDR q ≤ 0.05).

The preceding analyses are computational; can natural proteins fold without glutamine? To address this, we searched for glutamine-free orthologs of *E. coli* proteins across 6,369 complete prokaryotic proteomes from RefSeq. Orthologs were defined as reciprocal best hits with MMseqs2 (E-value < 1e-15). Of the 4,363 *E. coli* proteins, 103 already lack glutamine. Among the remaining 4,260, we found 948 with at least one ortholog completely lacking this amino acid. Thus, at least 24% of *E. coli* proteins (1,051/4,363) have natural orthologs that lack glutamine. Collectively, proteins with glutamine-free orthologs contain 16% of all glutamines in *E. coli* (9,405/59,955). We then asked whether glutamine-free orthologs are more or less common than expected. Across all 20 amino acids, the fraction of proteins with amino-acid-free orthologs decreases with abundance in *E. coli*—rare amino acids are more likely to be absent by chance (Fig 7). Three amino acids lie notably above this trend: Lys, Asn, and Gln, with more amino-acid-free orthologs than the fitted abundance relationship would place them. This places glutamine among the few residues that appear unusually dispensable in natural proteomes. Even within this limited sample of 6,369 proteomes, hundreds of *E. coli* proteins have glutamine-free orthologs; larger datasets will likely reveal many more.

**Fig 7:**
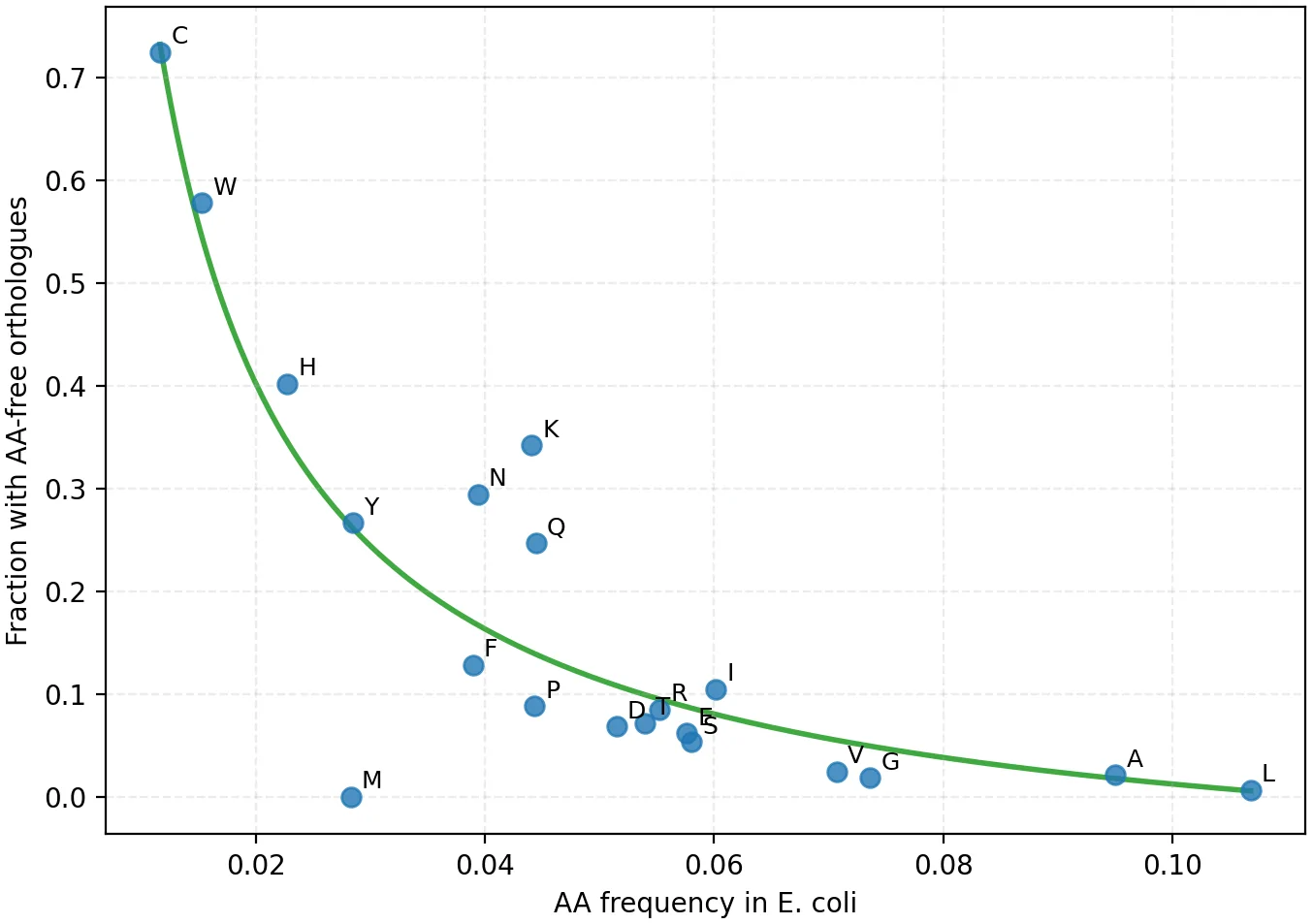
Amino-acid-free orthologs in prokaryotic proteomes. For each of the 20 amino acids, the fraction of *E. coli* proteins (out of 4,363) possessing at least one ortholog completely lacking that amino acid is plotted against the amino acid’s abundance in the *E. coli* proteome. Orthologs were identified as reciprocal best hits (MMseqs2, E-value < 1e-15) across 6,369 complete prokaryotic proteomes from RefSeq. Green curve: descriptive fit (y = a + b·x^−p^, nonlinear least squares, a = −0.099, b = 0.013, p = 0.933) summarizing the overall relationship — rarer amino acids tend to have more orthologs lacking them. Three amino acids lie notably above the fitted trend: lysine (K), asparagine (N), and glutamine (Q), which have more amino-acid-free orthologs than their abundance places on the curve. For glutamine, 1,051 *E. coli* proteins (24%) have at least one glutamine-free ortholog.

### Keep-score discriminates: Isoleucine is harder to remove than glutamine

A recurring concern with pipelines that both design and evaluate sequences is circularity: if ProteinMPNN and AlphaFold2 share structural priors, a glutamine-free proteome might fold well simply because the design tool avoids whatever the folding tool would penalize — in which case any amino acid, not glutamine specifically, would appear replaceable. We therefore tested whether the framework discriminates among amino acids of differing keep-score. We compared glutamine removal with removal of isoleucine, a higher-keep-score residue that Liu et al. eliminated experimentally from the *E. coli* ribosome [5], and included glycine — among the least replaceable residues — as a positive control that must register as disruptive. Across 571 proteins (the QIG_571_msa set; Methods), with each residue replaced by its most frequent MSA alternative and modeled with AlphaFold2, structural impact increased in the order Q < I << G (Fig 8A). All three differences were significant (paired Wilcoxon P = 1.7 × 10^−11^ for Q vs I, and P < 10^−51^ for both comparisons against G): glutamine removal was less disruptive than isoleucine removal in 65% of proteins, and glycine was substantially more disruptive than either. The pipeline is therefore not uniformly permissive; it ranks these residues in the order their keep-scores predict.

**Fig 8:**
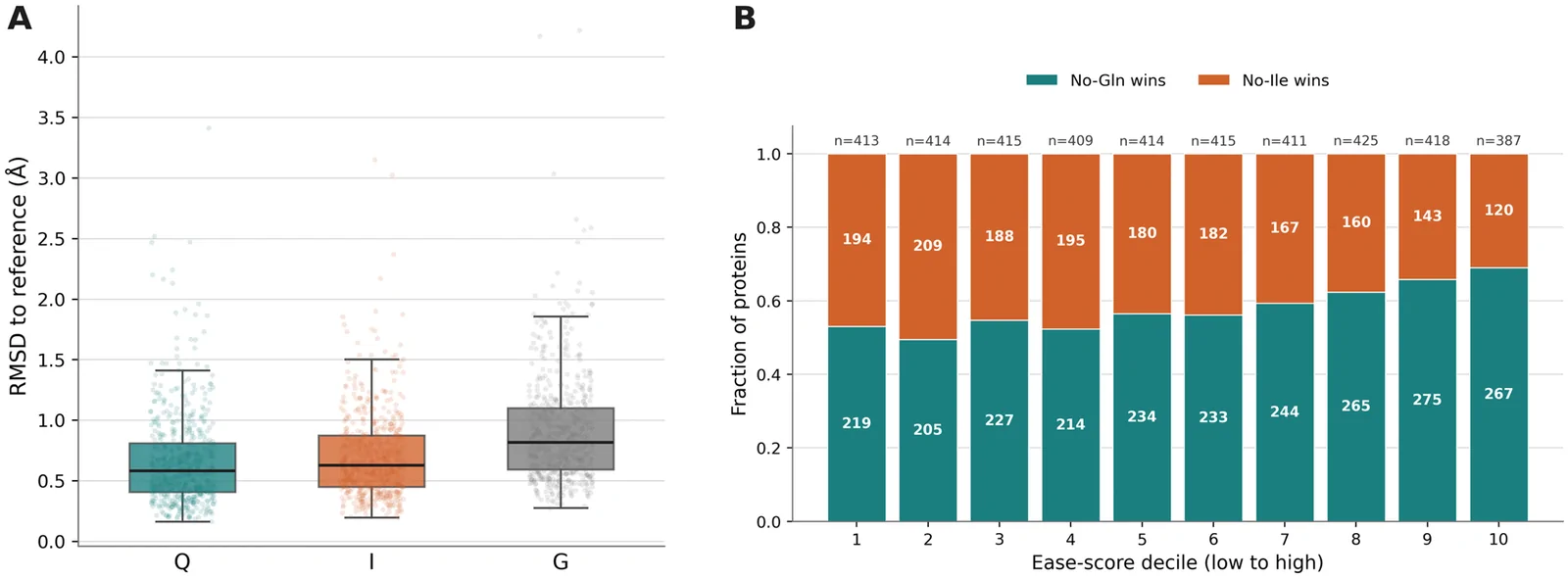
Isoleucine is harder to remove than glutamine. Structural impact of eliminating an amino acid, quantified as RMSD to the AlphaFoldDB reference (mean of five AlphaFold2 models per sequence). **(A)** Per-protein RMSD after removing glutamine (Q), isoleucine (I), or glycine (G) from each of 571 proteins (the QIG_571_msa set; Methods). Boxes: median and interquartile range; whiskers: 1.5 × IQR; points: individual proteins. RMSD increases in the order Q < I << G (medians 0.58, 0.63, and 0.82 Å); glycine, among the least replaceable residues, serves as a positive control. All three pairwise differences are significant (paired Wilcoxon P = 1.7 × 10^−11^ for Q vs I; P < 10^−51^ for both comparisons with G). Glutamine removal was less disruptive than isoleucine removal in 65% of proteins. **(B)** Proteome-wide comparison of glutamine versus isoleucine removal (the IQ_full set; Methods; all 4,148 proteins, each with both variants). Proteins are grouped into ease-score deciles (low to high); bars show the fraction in which No-Gln versus No-Ile had the lower RMSD, with per-decile counts and totals (n) annotated. The 27 proteins with identical RMSD for the two variants are ties and are omitted from the bars, so the per-decile totals sum to 4,121. The No-Gln win fraction increased overall from approximately 0.53 in the lowest ease-score decile to 0.69 in the highest. Across the full proteome, No-Gln had lower RMSD in 57.4% of proteins (median ΔRMSD = −0.024 Å; paired Wilcoxon P = 1.1 × 10^−15^).

We extended the glutamine-versus-isoleucine comparison to the whole proteome, removing each residue from all 4,148 proteins with ProteinMPNN-optimal replacements (the IQ_full set; Methods; Fig 8B). Here the two variants were nearly indistinguishable by TMscore (mean 0.9076 vs 0.9073; paired t-test P = 0.57) — an expected consequence of the design method, since ProteinMPNN selects replacements that reproduce the native fold regardless of which residue is removed, compressing the very difference we sought to measure. This is why the MSA-based comparison above, which does not optimize for structure, resolves the per-residue difference more cleanly. By the more sensitive RMSD metric, however, glutamine removal remained the less disruptive of the two across the full proteome (lower RMSD in 57.4% of proteins; median ΔRMSD = −0.024 Å; paired Wilcoxon P = 1.1 × 10^−15^). This advantage tracked structural reliability: binning proteins by ease-score, the fraction in which glutamine removal was less disruptive rose from ~0.53 in the lowest-ease decile to ~0.69 in the highest. Where AlphaFold2 determines structure most reliably, and prediction noise is smallest, the systematic advantage of removing glutamine over isoleucine emerges most clearly — as expected if the difference is real but subtle. Together these results show that the framework does more than rank glutamine lowest: it correctly orders residues of differing replaceability, confirming that the near-native folding of the glutamine-free proteome reflects a genuine property of glutamine rather than an artifact of the design-and-evaluate pipeline.

Having shown that keep-score predicts structural impact within the *E. coli* proteome, we asked whether the ranking itself generalizes beyond it. We computed ProteinMPNN keep-scores for *Saccharomyces cerevisiae*, a eukaryote separated from *E. coli* by over a billion years of evolution (Fig 9). The keep-scores of all 20 amino acids correlate strongly between the two organisms, and in both cases glutamine has the lowest keep-score by a wide margin. This consistency across more than a billion years of evolutionary divergence indicates that, from ProteinMPNN’s perspective, keep-score reflects intrinsic properties of protein structure rather than the composition of any single proteome.

**Fig 9:**
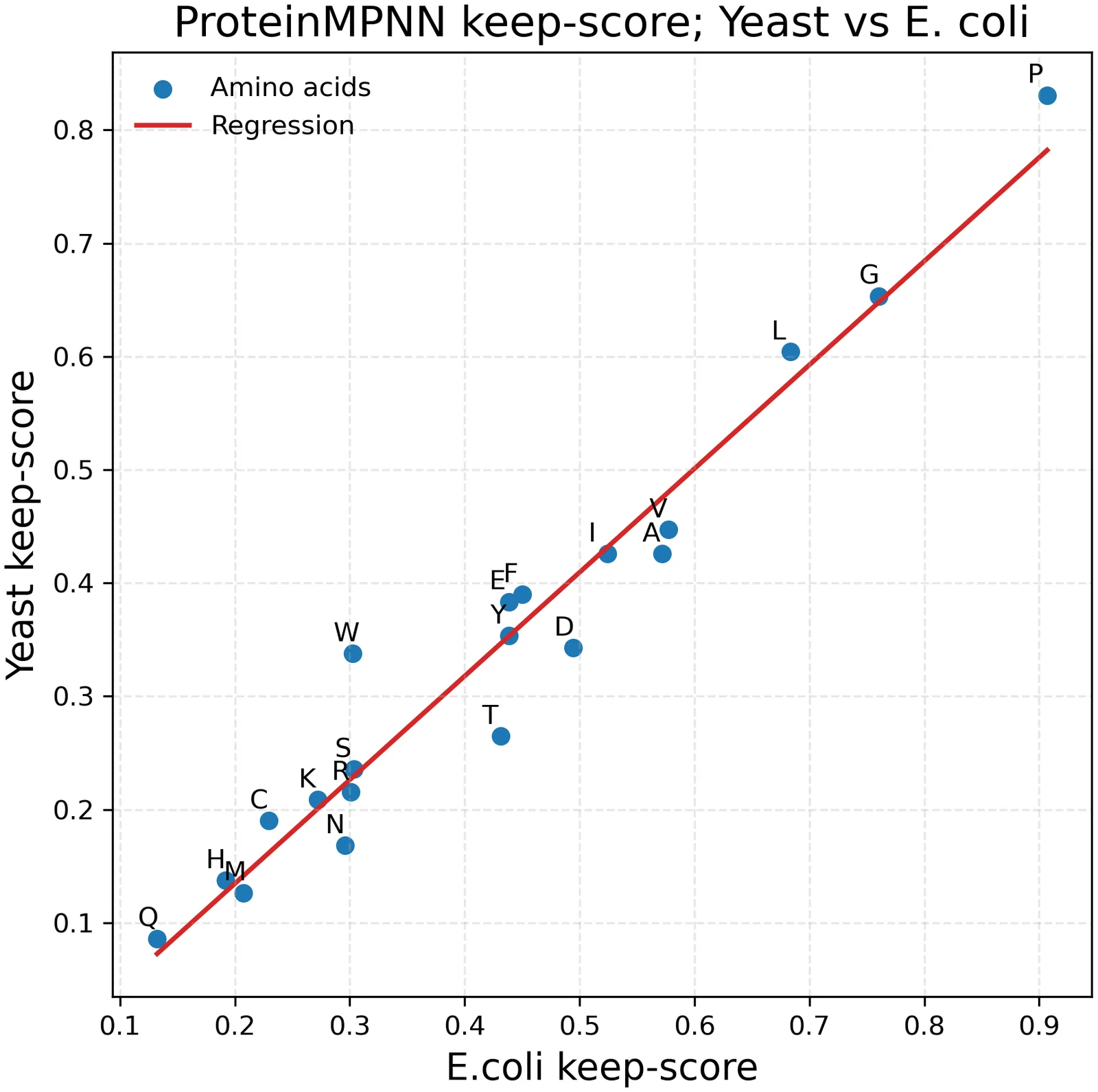
Keep-score is stable across domains of life. ProteinMPNN keep-scores (fraction of positions where the native residue is the highest-probability choice) computed independently for the *Saccharomyces cerevisiae* proteome (6,055 proteins; y-axis) and the *Escherichia coli* proteome (4,363 proteins; x-axis). Keep-scores correlate strongly across these two organisms, separated by over a billion years of evolution (Spearman ρ = 0.98, P = 7 × 10^−14^; Pearson r = 0.97). Red line: ordinary-least-squares regression (yeast keep = 0.92 × *E. coli* keep − 0.049; R² = 0.95). In both proteomes, glutamine (Q) has the lowest keep-score by a wide margin, and proline (P) and glycine (G) have the highest. Single-letter amino acid codes are shown next to each point.

### A glutamine-free β-lactamase is enzymatically active

The preceding analyses identify glutamine as the residue with the lowest keep-score and, by both structural and evolutionary criteria, among the most replaceable. As an independent consistency check that a glutamine-free protein can fold and function in vivo, we selected TEM-1 β-lactamase, a well-characterized periplasmic enzyme whose activity requires correct folding, Sec-dependent export, signal-peptide cleavage, and precise assembly of the catalytic triad (Ser70, Lys73, Glu166) and the oxyanion hole — none of which involves a glutamine. The mature enzyme contains eight glutamines; we replaced all eight with ProteinMPNN-optimal alternatives, retaining the single glutamine of the signal peptide, which is cleaved from the mature protein, to produce the variant TEM1-Q. Modeled without the leader peptide, TEM1-Q was predicted to adopt a structure essentially identical to native TEM-1 (TMscore 0.999, RMSD 0.16 Å) with the catalytic triad superposed, and had a lower Rosetta score than native TEM-1 after relaxation (−878.4 versus −865.4 REU). We therefore tested directly whether this predicted glutamine-free enzyme folds and functions.

Both constructs — the standard pQE30 vector, whose ampicillin-resistance marker is native TEM-1, and a derivative in which that resident coding sequence was replaced by TEM1-Q — were transformed into *E. coli* BL21 and conferred similar ampicillin resistance: each grew at up to 400 µg/mL ampicillin, and neither grew at 800 µg/mL. Because the two plasmids are identical except for the glutamine codons of a single gene, the comparison is exact. To compare activity at matched extract loading, we resolved soluble-fraction cell extracts and detected β-lactamase in situ with the chromogenic substrate nitrocefin (S12 Fig). Native TEM-1 and TEM1-Q produced activity bands of comparable intensity, and halving the load halved the signal for both, while the untransformed host showed none. At the expected position, neither enzyme produced a band above the cellular-protein background in the total-protein stain, so activity — not an abundant band — is the readout. A glutamine-free β-lactamase therefore folds into a catalytically competent enzyme, conferring native-like ampicillin resistance and comparable in-gel activity at matched extract loading.

## Discussion

The central finding of this study is that an entire canonical amino acid can be removed from a bacterial proteome without large structural consequences for essentially any of its proteins, and that both keep-scores singled out glutamine, a priori, as the best target for removal. Within the resolution of current computational methods, the *Escherichia coli* proteome achieves its native three-dimensional folds without it. When every glutamine was replaced with the highest-probability alternative under ProteinMPNN, the resulting sequences produced AlphaFold2 models that, as a population, matched the rebuilt-native baseline: mean TMscore of 0.914 versus 0.917 for rebuilt natives, with 85.5% exceeding the 0.8 threshold compared with 85.9% for the baseline (Fig 4). This result acquires particular significance in light of Liu et al. [5], who experimentally eliminated isoleucine from the *E. coli* ribosome as a first step toward a 19-amino-acid organism. Their work establishes that amino acid alphabet reduction is biologically feasible; ours addresses the prior question of which amino acid to target and provides a systematic framework for making that choice. The convergence of these two independent lines of work — one experimental, one computational — strengthens the conclusion that the 20-amino-acid alphabet is not a hard constraint of cellular life.

The identification of glutamine as the best a priori target for removal emerges from the agreement of two independent methods. ProteinMPNN, trained to predict sequences compatible with a given backbone structure, and MSA-derived conservation, reflecting billions of years of evolutionary selection, both assign glutamine the lowest keep-score among the 20 canonical amino acids (Fig 1). We do not claim that this definitively establishes glutamine as the “most dispensable” amino acid in a biological sense — dispensability encompasses function, regulation, and metabolic integration beyond structural integrity.

Rather, we report two distinct but consistent findings: glutamine has the lowest keep-score of any amino acid under both methods, and its removal is among the least structurally perturbing across the proteome — significantly less disruptive than removal of eleven higher-keep-score residues, yet indistinguishable from the small group of other low-keep-score residues (Fig 3). Both methods also agree that proline and glycine are the least removable, consistent with their unique roles in defining backbone geometry. This convergence argues against the possibility that glutamine’s replaceability is an artifact of any single algorithm. When two orthogonal approaches — one optimizing for structural compatibility, the other summarizing evolutionary constraint — reach the same conclusion, the signal likely reflects genuine biophysical properties of protein architecture. The keep-score and keep-propensity metrics introduced here provide a general framework for ranking amino acid replaceability at proteome scale and should prove useful for guiding future alphabet-reduction efforts.

A striking feature of glutamine’s replaceability is that it cannot be explained by the availability of a physicochemically similar substitute. When glutamine is replaced, glutamate — its closest chemical relative and the residue with which it is grouped in reduced-alphabet hierarchies [16,17] — is favored in only approximately 14% of cases (S4 Fig). This contrasts sharply with isoleucine, where over 30% of replacements favor valine, yet isoleucine is not among the most removable amino acids by keep-score. Classical substitution matrices, which quantify pairwise similarity based on evolutionary exchange rates, would predict that residues with obvious substitutes are most replaceable. Our data contradict this intuition: replaceability appears to be an emergent property of context-dependent structural constraints rather than a consequence of chemical similarity to a single alternative. This lack of a dominant substitute is itself informative: it points to the absence of a unique structural requirement rather than to any single obvious replacement. Glutamine’s surface amide contributes to solvation, helix propensity, or polar turn-bridging depending on position, and each of these functions can be satisfied by any of several residues — so different positions recruit different substitutes and none predominates. In contrast, glycine and proline show prominent retention peaks in their keep-propensity distributions (S2 Fig), reflecting their irreplaceable roles in backbone flexibility and rigidity, respectively.

If that constraint is context-dependent, it should vary from position to position, and the glutamines that conservation and design retain should be those in the more constrained settings. This is what we find. Partitioning every reference glutamine by solvent accessibility, buried glutamines (relative accessibility below 0.20) are retained far more often than exposed ones: glutamine is the most conserved residue in its alignment column at 68% of buried positions versus 42% of exposed ones, and ProteinMPNN likewise retains it more often when buried (28% versus 9%; S13 Fig). By MSA conservation the margin favoring glutamine is positive at buried positions and essentially absent at exposed ones (median per-position keep-propensity +0.35 versus −0.05). ProteinMPNN’s continuous keep-propensity, by contrast, does not separate the two groups — it remains negative for both — so buried glutamines are retained more often, though not more strongly favored by every metric. Secondary-structure context shows the same directionality far more weakly: by retention rate, glutamines in helices and strands are kept somewhat more often than those in coil under both methods, though the effect is small (Cramér’s V = 0.0824) and explains little of the variation in retention. This coupling of glutamine retention to burial has an independent, pre-AI precedent. Analyzing conservation across sequence alignments, Fiser et al. [31] reported that glutamine is the least conserved of all twenty amino acids and that, across residues, conservation declines with solvent accessibility — with glutamine falling off that trend, less conserved than even its high exposure would predict. Their population-level observation, reached decades earlier and without reference to any structure-prediction model, is consistent with our per-position finding that it is the buried minority of glutamines that tend to be retained. Taken together, these observations indicate that whether a glutamine is retained is governed largely by its local structural environment — chiefly whether the position is buried — rather than by any intrinsic property of the residue in isolation. Surface exposure may also offer a common root for the agreement of our two keep-scores: an exposed glutamine tends to be both structurally unconstrained, which ProteinMPNN registers as a low retention probability, and weakly conserved across homologues, which the alignments register directly — even though structural and evolutionary exchangeability, convergent here, are distinct properties.

A potential confound in ranking amino acids by removal impact is abundance: the residues whose removal perturbs structure most — glycine, valine, alanine, leucine — are also among the most common, and across the twenty amino acids removal impact is correlated with frequency (Spearman ρ = 0.73). This correlation is unsurprising, since residues under strong structural constraint should be both harder to remove and, over evolutionary time, more abundant; it nonetheless raises the question of whether keep-score predicts impact only because it tracks abundance. It does not. Controlling for amino-acid frequency, keep-score remains strongly associated with median removal RMSD (partial Spearman ρ = 0.74; Freedman–Lane P = 0.0003), whereas frequency controlling for keep-score is weak and not significant (ρ = 0.43, P = 0.065; S14 Fig). Keep-score therefore carries structural information beyond composition, while composition adds little beyond keep-score: glutamine is the best a priori target because its low keep-score reflects genuine structural permissiveness, not merely the ease of removing a rare residue.

Consistent with this picture, the global topological complexity of a fold does not predict how well it tolerates glutamine removal. Across the proteome, a protein’s relative contact order — a measure of how non-local its stabilizing contacts are — predicts neither which glutamines are retained (Spearman ρ = −0.002 for keep-score and 0.010 for keep-propensity; both non-significant) nor how well the fold tolerates their removal (ρ = −0.044; P = 0.009, n = 3,581): a significant but negligible correlation, if anything toward greater tolerance — indicating no penalty for recoding topologically complex proteins. We also examined proteins predicted to contain knots, an extreme topological feature that affects only about 0.4% of proteins in any proteome, *E. coli* included [32]. Only 9 of the 1,589 well-ordered chains we analyzed (Rebuilt PAE < 5) were predicted to be knotted, and glutamine removal in these did not measurably affect their structures (change in RMSD) relative to the unknotted set (one-sided Mann–Whitney P = 0.383; one-sided Welch t-test P = 0.138). Knotted chains did, however, retain glutamine somewhat more than unknotted ones (median Q-keep-score 0.67 vs 0.50; Q-keep-propensity 0.36 vs 0.10; two-sided Mann–Whitney P = 0.075 and 0.012; Welch two-sample t test P = 0.042 and 0.028, respectively), though with only nine knotted chains these differences are tentative. Knotting, where detectable, therefore tracks greater glutamine retention rather than reduced tolerance to its removal. Any effect of discrete topology on removal tolerance is thus at most marginal and does not alter the proteome-wide conclusion. Structural tolerance of glutamine removal is thus governed by local environment rather than global fold complexity.

The comparison with isoleucine removal is instructive beyond the question of target selection. Liu et al. [5] found that simple Ile-to-Val substitution — the most obvious replacement for a branched-chain amino acid — succeeded in only 43% of essential proteins tested. The remaining cases required iterative application of ESM2, MSA Transformer, ProteinMPNN, and AfDesign, with compensatory mutations at structural neighbor positions, and a median of four design attempts per gene. Three ribosomal genes resisted all computational approaches entirely and were solved only by random mutagenesis, yielding substitutions (Ile to Gly, Trp, or Phe) that no model had predicted. Correspondingly, our proteome-wide glutamine removal — using only the top ProteinMPNN prediction at each position, without compensatory mutations or iterative optimization — preserved native folds in 85.5% of proteins, matching the rebuilt baseline. These are different measures — computational fold preservation versus experimental fitness — not the same scale. We therefore tested the comparison directly, removing isoleucine across the same proteome with the same pipeline used for glutamine: isoleucine is the more disruptive of the two to remove (Fig 8), a computational counterpart to the experimental isoleucine removal of Liu et al. This is what the keep-score framework predicts — a lower keep-score corresponds to easier elimination, because fewer positions impose strong constraints on the resident amino acid.

A recognized concern with pipelines that both design and evaluate sequences is circularity: ProteinMPNN and AlphaFold2 are trained on overlapping structural data, so a glutamine-free proteome might fold well in silico simply because the design tool avoids what the evaluation tool would penalize, and because the AlphaFoldDB references are themselves predictions, the comparison is in part of predicted models against predicted models. Several features of our analysis argue that the result is not merely this artifact. First, the framework discriminates: removing isoleucine — a higher-keep-score residue — is measurably more disruptive than removing glutamine across the same proteome and pipeline (Fig 8), and glycine, a positive control, is more disruptive still. A pipeline that only reproduced shared training bias could not separate these residues in the order their keep-scores predict. Second, the two keep-scores are methodologically independent: MSA conservation uses no structural model and shares no training data with ProteinMPNN or AlphaFold2, yet converges on the same ranking (Fig 1), and sequences designed by one criterion are predicted as replaceable by the other. Third, our baseline is an independent reproducibility metric rather than model self-confidence — the ease-score is the TMscore of the native rebuild against its AlphaFoldDB reference, computed without recourse to pLDDT or PAE, so it normalizes precisely for the predicted-versus-predicted concern by asking only whether a redesigned sequence reproduces its own reference as faithfully as the native sequence does. We nonetheless concede a real limit of the design method: because ProteinMPNN selects replacements that reproduce the native backbone whatever residue is removed, it folds an isoleucine-free proteome nearly as well as a glutamine-free one by TMscore, compressing the per-residue difference (Fig 8). This is why we resolve that difference with the more sensitive RMSD metric and with MSA-guided designs, which do not optimize for structure — and why ProteinMPNN’s fold-reproducing tendency is itself the reason we cross-validate every conclusion against evolutionary conservation.

We validated the structural integrity of the glutamine-free proteome through three complementary computational approaches, each targeting a different potential failure mode: local confidence at substitution sites, Rosetta energetics, and an orthogonal structure predictor (ESMFold) — whose capacity to interpret reduced-alphabet sequences has been independently demonstrated by Ieremie et al. [28]. All three approaches confirmed that glutamine removal does not compromise fold integrity (Fig 5). For the approximately 14% of No-Gln models falling below the TMscore 0.8 threshold, FoldMason local alignment, flexible superposition, and comparison with BioEmu conformational ensembles consistently indicated that low scores reflect intrinsic protein flexibility rather than misfolding (Fig 6). We found no evidence that removing glutamine causes structural failure in any *E. coli* protein.

The TEM1-Q experiment provides a single but informative data point. It does not validate the proteome-wide analysis — that would require systematic experimental testing of essential proteins, as Liu et al. [5] performed for isoleucine in the ribosome. What it does show is that the computational pipeline produces predictions consistent with biological function in at least one well-characterized case, and that glutamine removal is compatible with the full chain of events required for a periplasmic enzyme: translation, secretion, processing, and catalysis. Expressed from the same vector and regulatory context as native TEM-1, so that the two differ only by the eight glutamine substitutions, TEM1-Q matched the native enzyme’s resistance profile: both supported growth at ampicillin concentrations up to 400 µg/mL and neither grew at 800 µg/mL. An in-gel activity assay corroborated this: total cell extracts of the two strains showed comparable β-lactamase (nitrocefin) activity at the same migration position (S12 Fig). By both a whole-cell resistance phenotype and an in-gel activity assay, glutamine removal leaves folding and catalytic function intact to the resolution of these assays.

ProteinMPNN-guided glutamine removal substantially outperformed MSA-based removal, with 85.5% of models above threshold compared with 78% for MSA-guided designs. This difference likely reflects the distinct information captured by each method. ProteinMPNN leverages structural context and long-range packing constraints to identify substitutions compatible with the native fold, whereas MSA consensus preserves residues that have persisted through evolutionary history — a signal that includes functional constraints, translational efficiency, and phylogenetic inertia beyond fold stability alone. Notably, in the optimal-recoding analysis, ProteinMPNN preserved native folds at much lower sequence identity: 46% compared with 66% for MSA consensus (S7 Fig). This demonstrates that the sequence space compatible with natural protein folds is vastly larger than what evolution has explored, and that structural compatibility is less restrictive than evolutionary conservation suggests. That inverse-folding models can navigate severely restricted sequence spaces has been confirmed experimentally: Giacobelli et al. [25] used ProteinMPNN and RFdiffusion to design de novo proteins from a 10-amino-acid alphabet, obtaining stable structures that adopted their intended topologies and resisted thermal and chemical denaturation. Liu et al. [5] reached a similar conclusion from the experimental side: their most challenging ribosomal designs required structural models (ProteinMPNN, AfDesign) rather than evolutionary ones (ESM2, MSA Transformer), and the successful designs often occupied regions of sequence space distant from any natural homologue. The convergence of these observations across two independent studies and two different amino acids reinforces the view that inverse-folding models capture design principles that evolutionary sampling has not fully explored.

The computational finding that glutamine is dispensable aligns with natural evolutionary evidence. Among the 4,363 *E. coli* proteins, 103 already lack glutamine natively, and among the remaining 4,260, we identified 948 with at least one glutamine-free ortholog across 6,369 prokaryotic proteomes. Thus, at least 24% of *E. coli* proteins have natural orthologs that lack this amino acid. When we examined all 20 amino acids, glutamine was among three residues — alongside lysine and asparagine — lying above the fitted abundance trend in amino-acid-free orthology (Fig 7). This places glutamine among the few residues that appear unusually dispensable in natural proteomes, consistent with our computational results reflecting genuine biological possibility. The keep-score ranking is not confined to *E. coli*: ProteinMPNN keep-scores for the *Saccharomyces cerevisiae* proteome correlate strongly with those of *E. coli* across all twenty amino acids, with glutamine lowest and proline and glycine highest in both (Fig 9). That the ranking holds across more than a billion years of evolutionary divergence indicates that, from ProteinMPNN’s perspective, keep-score reflects intrinsic properties of protein structure rather than the composition of any single proteome.

If glutamine is so readily replaceable, why has every known organism retained it in the genetic code? Several considerations may resolve this apparent paradox. First, glutamine serves essential metabolic functions as the primary carrier of organic nitrogen in cellular metabolism; its abundance in proteomes may be driven by biosynthetic convenience and metabolic integration rather than structural necessity. Second, our study demonstrates fold preservation and, in the case of TEM-1, catalytic activity, but glutamine residues in other active sites, protein-protein interfaces, allosteric networks, or post-translational modification sites may be essential for function even when the surrounding fold is maintained. A protein that folds correctly without glutamine may nonetheless be catalytically dead or unable to participate in essential interactions — a distinction that genome-wide experimental testing will be needed to resolve. Third, theories of genetic code evolution often propose that glutamine was a late addition to the amino acid alphabet, biosynthetically derived from glutamate after the core protein architecture had already been established [1,33]. Our finding that modern proteins can be recoded without glutamine while preserving their folds is consistent with this hypothesis: if the ancestral proteome evolved before glutamine entered the repertoire, contemporary structures may retain the capacity to function without it. Finally, the genetic code may simply be frozen in a suboptimal state — removing an amino acid would require coordinated changes across thousands of proteins, a barrier that may be insurmountable through natural evolution even if the endpoint is structurally viable. The experimental framework of Liu et al. [5] shows that synthetic biology can overcome this barrier one protein complex at a time.

Several limitations warrant acknowledgment. This study is primarily a computational analysis; apart from the single TEM1-Q consistency check, all evidence for fold preservation comes from structure prediction algorithms. AlphaFold2 may systematically underestimate the structural impact of mutations because it was trained predominantly on native sequences, potentially biasing outputs toward folded conformations. We mitigated this concern through validation with ESMFold, Rosetta energetics, and comparison with BioEmu conformational ensembles, but we cannot exclude that some proteins scored as correctly folded would in fact misfold experimentally.

A further limitation concerns the design step rather than the evaluation. Because each glutamine replacement was chosen from the ProteinMPNN probability table conditioned on the native sequence (Methods), the substitutions represent per-position marginal optima and do not model second-order coupling among the co-substituted positions; the recoded proteins were not jointly re-optimized. This concern is mitigated by the readout: because fold evaluation was performed on the fully recoded sequence, destabilization arising from coupling among the simultaneous substitutions would register in the AlphaFold2, ESMFold, Rosetta, and BioEmu comparisons, and none was detected. Consistent with this, Liu et al. [5] found that isoleucine removal frequently required compensatory mutations at structural-neighbour positions, whereas single top-choice glutamine replacement, without compensatory changes, preserved native folds in 85.5% of proteins — as expected if glutamine tends to occupy the less constrained, less coupled positions. The AlphaFoldDB references against which we compare are themselves predictions, and our ease-score normalization, while controlling for prediction variability, does not eliminate this dependency. Additionally, our analyses excluded proteins shorter than 50 or longer than 890 residues. Within these bounds, we found no evidence of misfolding attributable to glutamine removal using any available computational method — but absence of evidence from prediction tools is not evidence of absence of misfolding *in vivo*. A further limitation is intrinsic to the readout rather than the tools. Our analysis evaluates whether reduced-alphabet sequences are compatible with the native structural state, not the kinetics by which that state is reached; preservation of the predicted native fold therefore does not establish preservation of folding rates, kinetic stability, folding cooperativity, or the avoidance of off-pathway intermediates. This distinction is not merely formal. Using alphabet size as a proxy for evolutionary time, Faísca and co-workers found that the folding consequences of a reduced alphabet depend strongly on topology: unknotted and shallowly knotted proteins largely retained two-state cooperativity and efficient folding under an ancestral ten-amino-acid alphabet, whereas a deeply knotted protein had its cooperativity and folding ability significantly compromised [34]. A companion study, isolating topology in structure-based simulations, found that kinetic stability rises with knot depth and that enlarging the amino-acid alphabet predominantly enhances kinetic stability [35]. Native-state structure can thus appear preserved while kinetic and cooperative properties are not — a caveat that applies directly to conclusions, such as ours, drawn from structural and stability metrics alone.

Enzymes in which a glutamine residue participates directly in catalysis would provide the most stringent tests of whether fold preservation translates to functional preservation. It would also be informative to ask where glutamine persists despite low keep-score. The three metrics used here are complementary by design: ProteinMPNN keep-score reflects structural constraint alone, MSA keep-score summarizes the evolutionary behaviour of glutamines in bulk, and the fraction of positions conserved in over 99% of orthologues isolates the minority retained across evolution despite low structural constraint — a set enriched for glutamines essential for reasons beyond fold stability, such as catalysis, binding, post-translational modification, or regulation. The ~ 7% of glutamine positions so conserved (S15 Fig) may therefore mark functionally critical sites worthy of targeted investigation. Two further questions remain open: whether lysine and asparagine — the other amino acids lying above the abundance trend in amino-acid-free orthology — are similarly eliminable, and whether multiple amino acids can be removed simultaneously before a hard structural barrier is reached. On the latter, the demonstration by Shibue et al. [6] that an ancestral nucleoside kinase retains solubility, stability, and catalytic activity with only 13 amino acid types suggests that the barrier may lie well beyond single-amino-acid removal, at least at the level of an individual protein.

The iterative design-build-test framework established by Liu et al. [5] for isoleucine-free ribosomal proteins provides a direct experimental path to test whether the keep-score ranking predicts biological outcome. Systematic replacement of glutamine in essential *E. coli* genes, followed by fitness measurements, would reveal whether computational structural replaceability translates to experimental viability — and whether glutamine removal is indeed more tractable than isoleucine removal, as our analysis predicts. Liu et al.'s demonstration that an essential complex can be rebuilt without one canonical amino acid, combined with the computational framework developed here for rational target selection, together provide a roadmap toward identifying the minimal amino acid alphabet compatible with cellular life.

## Materials and methods

### External data sources

Reference 3D models for the complete proteome of *Escherichia coli* K-12 (UniProt Proteome ID UP000000625) were downloaded as PDB files from the AlphaFold Protein Structure Database version 4 (AlphaFoldDB) [36] as a tar archive (https://ftp.ebi.ac.uk/pub/databases/alphafold/latest/UP000000625_83333_ECOLI_v4.tar), which includes 4,363 single-chain models. Native sequences were extracted from these PDB files with a Python script.

The *Saccharomyces cerevisiae* proteome was similarly downloaded from AlphaFoldDB (UniProt Proteome ID UP000002311) with 6,055 single-chain models.

From UniProt Reference Proteomes (https://www.uniprot.org/proteomes) we downloaded all 9,285 Eubacterial proteomes as of September 14, 2024. This “eubacterial” collection was the source of all multiple sequence alignments (see below).

From the same database we downloaded all 9,371 Eubacterial and 349 Archaeal proteomes as of October 26, 2024. This “prokaryotic” collection was used only to determine per-species amino acid compositions.

From NCBI RefSeq (https://www.ncbi.nlm.nih.gov/refseq/about/prokaryotes/), we downloaded 6,369 complete reference prokaryotic proteomes as of October 1, 2025. These were used to identify orthologs of *E. coli* proteins lacking glutamine (see below).

### Conservation multiple sequence alignments

Proteins from the eubacterial collection were made non-redundant at 95% identity using MMseqs2 cluster [37]. We then searched this non-redundant database for homologs of the 4,363 *E. coli* native sequences with MMseqs2, run with its default --max-seqs 300; because the target database was split three ways, each query returned at most 420 hits. Homologs were read from the MMseqs2 tabular output (.m8), which is ordered by alignment quality. For queries with more than 200 homologs, every second entry of the quality-sorted.m8 (odd-indexed, i % 2) was dropped, halving alignment depth while preserving the quality-rank distribution; this decimation is deterministic, using no random selection and no seed. A conservation MSA was then built from each *E. coli* query and its retained homologs using MUSCLE [38]; no MSA was built for queries with fewer than 30 homologs. Alignments were trimmed to remove columns where the *E. coli* query had a gap. These conservation MSAs are the source of every sequence-conservation quantity in this work — keep-score and keep-propensity, replacement tendencies, MSA-consensus recoding, and MSA-guided glutamine replacement — and are distinct from the a3m alignments used as input to AlphaFold2 (see “In-house Prediction of 3D Structures”).

### ProteinMPNN probability tables

For each protein, the inverse-folding probability table was obtained with LigandMPNN version 1.5.5 [39] as the average of 10 scoring runs. All inputs were ligand-free protein backbones, and these probabilities are referred to as ProteinMPNN probabilities throughout this work. Parameters: --model_type 'ligand_mpnn' --seed 111 --single_aa_score 1 --use_sequence 1 --batch_size 1 --number_of_batches 10. With --single_aa_score 1 --use_sequence 1, each position’s amino-acid probabilities are evaluated in the context of the native residues at all other positions, so the per-position scores are structure-derived but conditioned on native sequence context rather than computed from backbone geometry alone. Scoring was therefore always performed on native, glutamine-containing backbones in native sequence context; no glutamine-free sequence was ever scored by ProteinMPNN, and each glutamine replacement was taken as the highest-probability non-glutamine residue from this native-context table. Averaging over 10 runs with a fixed seed makes the per-position probabilities reproducible while accounting for the stochastic decoding order. A position-specific scoring matrix (PSSM) was similarly derived from normalized amino acid frequencies in the corresponding conservation-MSA columns.

### Keep-propensity and keep-score

For any residue in a protein, its **keep-propensity** measures how strongly it is favored or disfavored at that position. From ProteinMPNN, it is the predicted probability of the native amino acid minus the probability of the highest-probability alternative. From MSAs, it is the frequency of the native amino acid minus the frequency of the most frequent alternative. Keep-propensity ranges from −1.0 (strong preference for replacement) to +1.0 (strong preference for retention).

An amino acid’s **keep-score** is the fraction of positions where the native residue scores higher than the best alternative—equivalently, the area under the positive region of its keep-propensity distribution. Keep-scores for the *S. cerevisiae* proteome were calculated similarly from ProteinMPNN probability tables.

### Conservation and replacement tendencies

For amino acid X, we calculated its conservation tendency as the mean frequency of X across all MSA columns where X is native (or, for ProteinMPNN, the mean P(X) at those positions). We also calculated 19-component replacement tendency vectors: for each amino acid Y ≠ X, the vector element is the mean frequency (or probability) of Y at positions where X is native. The 19 replacement tendencies were normalized to sum to 1.0.

### Solvent accessibility and glutamine burial

Solvent accessibility was computed for the AlphaFoldDB reference structures with DSSP [40]. For each glutamine, relative solvent accessibility (RSA) was taken as the DSSP absolute solvent-accessible surface area divided by 225 Å², the maximum accessibility reported for glutamine by Tien et al. [41]. Glutamines with RSA < 0.20 were classified as buried and the remainder as exposed. DSSP residues were mapped to one-based sequence positions within each chain (by chain identifier, residue number, and insertion code) so that structural and sequence records could be joined by protein identifier and position; the same DSSP output also provided the per-residue secondary-structure state used below.

For each glutamine position we drew two retention measures from its conservation-MSA column: an MSA keep indicator, set to 1 when glutamine was the most frequent standard amino acid in the column and 0 otherwise, and the per-position keep-propensity defined earlier (glutamine’s gap-normalized column frequency minus that of its most frequent non-glutamine alternative). ProteinMPNN retention was defined analogously from the probability table (glutamine the highest-probability residue, and its keep-propensity). Keep fraction and keep-propensity were then summarized separately for buried and exposed glutamines.

The association of burial with the binary keep indicator was tested by chi-square and point-biserial correlation (exposed coded as 1); keep-propensity was compared between buried and exposed positions by two-sided Mann–Whitney test and Spearman rank correlation.

We further tested whether glutamine retention depends on secondary structure, reading the DSSP state from the same records. States were grouped as helix (H, G), strand (E, B), and coil (I, T, S, the DSSP blank state, and poly-proline-II, P). Retention was summarized per class as the keep fraction (the MSA keep indicator) and as the gap-normalized keep-propensity defined above. The keep fraction differed across the three classes (chi-square = 398.4, 2 df, P = 3.0 × 10^−87^; Cramér’s V = 0.0824), as did keep-propensity (Kruskal–Wallis H = 413.3, 2 df, P = 1.8 × 10^−90^; epsilon-squared = 0.0070). Both effects are small despite the decisive P-values: glutamines in helices and strands are retained modestly more often than those in coil, with strands marginally above helices, and the association explains little of the position-to-position variation in retention.

### Contact order and fold topology

To ask whether global fold topology influences glutamine removability, we computed relative contact order (RCO) for the AlphaFoldDB reference structures following Plaxco et al. [42]. Intrachain contacts were defined between Cβ atoms (Cα for glycine): a residue pair was a contact when its representative atoms lay within 8 Å and the residues were separated by at least three positions in sequence. RCO was calculated as the sum of sequence separations |i − j| over all qualifying contacts, divided by the product of chain length and contact count. Residues were ordered by their PDB ATOM records, non-primary alternate locations were ignored, and proteins without qualifying contacts were assigned a missing RCO and excluded from correlation analyses.

We related RCO to two per-protein quantities. First, to test whether glutamine conservation tracks topology, we joined finite RCO values to the MSA-derived glutamine keep-score and keep-propensity (defined above), restricting to proteins with finite RCO and at least one reference glutamine, and repeated the analysis retaining only proteins with at least three reference glutamines. Second, to test whether topology predicts the structural cost of glutamine removal, we quantified per-protein sensitivity as ΔRMSD = RMSD(No-Gln) − RMSD(Rebuilt), restricting to proteins with finite RCO and Rebuilt TMscore > 0.8, and repeated the rank analysis at Rebuilt TMscore thresholds of 0.90 and 0.95. Associations were assessed with two-sided Spearman rank and Pearson product-moment correlations.

As an extreme topological feature, we also tested chain knotting. Backbone entanglement was classified with Knoto-ID [43], which evaluates open protein backbones as knotoids without imposing an arbitrary chain closure. We used the precomputed Knoto-ID output for the models whose Rebuilt structures had mean PAE < 5 Å, since disordered or flexible chains can mislead the knotoid classification; all 1,589 such chains returned a valid classification. Chains were assigned to the knotted or unknotted group by the Knoto-ID prediction field, yielding 9 knotted and 1,580 unknotted; “knotted” here denotes a predicted, not experimentally determined, topology. We asked whether knotted proteins were more sensitive to glutamine removal, quantifying sensitivity as ΔRMSD = RMSD(No-Gln) − RMSD(Rebuilt), computed independently for the ProteinMPNN (pMPNN_no_gln) and MSA (MSA_no_gln) glutamine-removal sequences, each against its own Rebuilt baseline and including only proteins with both RMSD values present. Because the a priori hypothesis was directional, knotted and unknotted groups were compared by one-sided Mann–Whitney and Welch two-sample t tests (alternative: greater). We separately compared the two groups’ glutamine keep-score and keep-propensity — with keep-score counting a position as Q-retained when Q was the most frequent residue in its alignment column (ties to Q) — by two-sided Mann–Whitney and Welch two-sample t tests, restricted to the 1,570 chains with a conservation MSA and at least one reference glutamine.

### In-house prediction of 3D structures

Sequences were converted to 3D structures with AlphaFold2 using LocalColabFold [26], by running colabfold_batch <a3m_input> <outdir> with default parameters (five models per sequence); no parameters were overridden.

Two a3m sources were used, and every model is tagged by which one:

- **Own a3m** (previous datasets): the a3m was derived from the protein’s conservation MSA (above).
- **ColabFold a3m** (new datasets): the a3m was generated by the ColabFold MMseqs2 server, obtained by running colabfold_batch --msa-only <native_fasta> <outdir>.

In both regimes a single a3m was built per protein, from the native sequence, and reused for every variant of that protein by replacing only the query line with the sequence to be modeled (native or variant) while preserving the alignment gaps; the a3m columns are therefore identical across all of a protein’s variants. Consequently, model-to-model comparisons are always made within a single a3m source — within each new dataset every variant of a protein shares one ColabFold a3m — and no model built from one a3m source is ever compared with a model built from the other.

### Structural datasets

The datasets below were generated from native *E. coli* sequences. Because recoding substitutes residues without insertion or deletion, every model has the same length as its native counterpart. Unless otherwise noted, analyses used the 4,148 proteins between 50 and 890 amino acids. Each dataset was modeled from one of two a3m sources (see “In-house Prediction of 3D Structures”), and models are never compared across a3m sources; the datasets are therefore grouped below by a3m source.

#### Datasets modeled from own a3m.

- **Reference** (4,363): the *E. coli* proteome as downloaded from AlphaFoldDB; native sequences, not remodeled in house.
- **Rebuilt** (4,148): native sequences remodeled in house with LocalColabFold; supplies the ease-score baseline.
- **mut_each_200** (4,000 models = 200 proteins × 20): for 200 selected proteins, each amino acid in turn was replaced at all of its occurrences by the highest-probability alternative according to ProteinMPNN. Proteins were selected for TMscore > 0.95 and mean PAE < 7 in Rebuilt models, > 400 homologs, length 250–550 aa, ≥ 5 occurrences of each amino acid, and no single amino acid exceeding 15% of the sequence.
- **Consensus** (3,960): the most frequent amino acid at each conservation-MSA column. Fewer than 4,148 because some columns lacked a robust consensus.
- **mpnn_ideal** (4,148): the highest-probability amino acid at each position according to ProteinMPNN.
- **pMPNN_no_gln** (also called **No-Gln**; 4,148): native sequences with every glutamine replaced by the highest-probability non-glutamine residue according to ProteinMPNN.
- **MSA_no_gln** (3,960): native sequences with every glutamine replaced by the most frequent non-glutamine residue at its conservation-MSA column. The count is below 4,148 because proteins with fewer than 30 homologs have no conservation MSA from which to design the replacement.

#### Datasets modeled from ColabFold a3m (all comparisons made within a set).

- **IQ_full** (8,296 models = 4,148 proteins × 2): each of the 4,148 proteins had glutamine removed and, separately, isoleucine removed, in both cases by ProteinMPNN-optimal replacement. The set contains a No-Gln and a No-Ile model for every protein and is the basis of the proteome-wide glutamine-versus-isoleucine comparison.
- **mut_each_300_msa** (6,000 models = 300 proteins × 20): for 300 proteins, each amino acid in turn was replaced at all of its occurrences by the most frequent alternative at its conservation-MSA column. Proteins were selected for ease-score ≥ 0.95, mean PAE ≤ 7, Rebuilt RMSD between 0.15 and 0.5 Å, length > 50 and < 890 aa, each amino acid present at least 3 times and constituting at most 15% of the sequence, and a conservation MSA present.
- **QIG_571_msa** (1,713 models = 571 proteins × 3): for 571 proteins, glutamine, isoleucine, and glycine were each removed in turn by most-frequent conservation-MSA-column replacement. Starting from proteins in which every one of the 20 amino acids constitutes at least 1% of the sequence, we selected those with length > 50 and < 600 aa, ease-score > 0.9, Rebuilt RMSD < 0.5 Å, and a conservation MSA present.

Two distinct No-Gln model sets exist and are reported separately: **pMPNN_no_gln** (own a3m; the headline No-Gln results, compared against the Rebuilt baseline) and the glutamine arm of **IQ_full** (ColabFold a3m; used only for the within-set comparison against isoleucine). These two No-Gln sets are never compared to each other.

### Structural evaluation

All structural models were compared to their AlphaFoldDB reference with USalign [44], run as USalign <query_pdb> <reference_pdb> -outfmt 2. From the tabular output we used two fields: TM1 as the TMscore (TM1 and TM2 are identical here, because query and reference have the same length and no gaps, so TMscore normalized by query length equals TMscore normalized by reference length) and the RMSD field as the RMSD in Å. The reference was always the corresponding model from the AlphaFoldDB *E. coli* proteome.

AlphaFold2 produces five models per sequence. TMscore, ease-score, and Rebuilt RMSD were taken from the best-ranked model (rank 001). For datasets modeled from ColabFold a3m, the RMSD reported for a sequence is the mean RMSD over all five models; the added averaging is used because these datasets support the fine per-residue RMSD comparisons where single-model prediction noise would otherwise dominate. The two conventions are never mixed within a single comparison.

Throughout this work, models with TMscore ≥ 0.8 are classified as structurally correct; those below 0.8 are flagged for further analysis. This threshold is deliberately conservative: a TMscore above 0.5 already indicates a shared fold [45], and we adopt 0.8 so that “structurally correct” denotes close backbone agreement rather than merely shared topology. The choice is also calibrated to our own baseline: 86% of native rebuilds exceed 0.8 against their AlphaFoldDB references (the ease-score; Fig 2A), and sub-threshold proteins have systematically high PAE (r = −0.81; Fig 2B), indicating intrinsic flexibility rather than misfolding.

### Ease-score and squared mean PAE

We define the **ease-score** of a protein as the TMscore of its Rebuilt model against its AlphaFoldDB reference. It measures how reliably AlphaFold2 reproduces the same structure from an identical sequence and serves as a baseline for interpreting TMscores of models from non-native sequences. By analogy, a protein’s **Rebuilt RMSD** is the RMSD of its Rebuilt (rank-001) model against its AlphaFoldDB reference — an RMSD counterpart to the ease-score. It is used as a selection covariate for the mut_each_300_msa and QIG_571_msa sets.

**Squared mean PAE** is calculated as the arithmetic mean of all pairwise predicted aligned error (PAE) values from AlphaFold2, squared: (mean PAE)². This transformation linearizes the relationship with TMscore.

For binned analyses (Figs 4 and S6), proteins were sorted by ease-score into 50 equal-sized bins, and each protein keeps its bin across recoding strategies, so strategies are compared on the same proteins within each bin — except the MSA-based series, which cover only the proteins with an available MSA.

### Local confidence analysis

To assess whether glutamine removal affected local structural confidence, we computed the mean pLDDT of residues within 7 Å of each substitution site. Distance was measured between Cα atoms: a residue was included if its Cα was within 7 Å of the Cα of the replaced glutamine. Mean neighborhood pLDDT was compared between Rebuilt and No-Gln models.

### Energetic stability analysis

Thermodynamic impact of glutamine removal was evaluated using PyRosetta (PyRosetta-4 2025.51; Rosetta Commons) [46]. Coordinate constraints were applied to all Cα atoms using a harmonic potential with standard deviation 1.0 Å, tethering the backbone to the initial AlphaFold2 prediction while allowing side-chain repacking and minor backbone adjustments. Structures were minimized using the FastRelax protocol [47] with the ref2015 energy function [48] and coordinate constraint weight 1.0.

The change in stability was calculated as:


$$\begin{array}{c} \Delta \text{Score}=\text{Score}(\text{No}-\text{Gln})-\text{Score}(\text{Rebuilt}) \end{array}$$

where scores are final Rosetta Energy Units (REU). Negative ΔScore indicates that the No-Gln variant is predicted to be more stable.

### Flexible domain alignments

Using a custom piecewise alignment script, we performed flexible superpositions between query models and their AlphaFoldDB references. Backbone atoms and per-residue pLDDT scores were extracted, and “ordered” segments were defined as contiguous regions with smoothed pLDDT ≥ 50 spanning at least 30 residues. Each ordered segment was aligned separately using USalign (-TMscore 1). Segmentation was retained only if it improved the coverage-weighted TMscore by ≥ 0.04 with median Cα deviation ≤ 3 Å. When only one ordered block was present, a single split at the largest local deviation peak was tested under the same criteria. Final scores were aggregated over the union of aligned residues, yielding a single coverage-weighted TMscore per structure pair. The procedure returned no result for 52 of the 600 sub-threshold proteins (S11 Fig).

### FoldMason alignments

Flexible alignments were also performed using FoldMason “easy-msa” (https://github.com/steineggerlab/foldmason, [49]) to compare Rebuilt and recoded models against AlphaFoldDB references. The primary metric was mean LDDT. Models were classified as **perfect** (mean LDDT = 1.0) or **good** (mean LDDT > 0.9).

### Conformational ensembles with BioEmu

BioEmu (https://github.com/microsoft/bioemu, [50]) was used to generate 50 conformational samples from the approximate equilibrium distribution of native-sequence structures. Unrealistic trajectories were filtered, leaving a mean of 38.6 (median 43) usable samples per protein. No usable ensemble was obtained for 103 of the 600 sub-threshold proteins (S11 Fig). Each sample was aligned to the AlphaFoldDB reference using USalign, and TMscore distributions were computed. Because each protein has a single No-Gln model, its No-Gln TMscore was tested as a one-sided outlier against the native BioEmu ensemble. For each protein the No-Gln value was standardized as (x − m)/(s·√(1 + 1/n)), where m, s, and n are the mean, standard deviation, and number of retained trajectory samples of the native ensemble, and a one-sided P-value was obtained from the t-distribution with n − 1 degrees of freedom (testing whether the No-Gln value lies below the ensemble mean). Per-protein P-values were corrected across proteins by the Benjamini–Hochberg procedure.

### ESMFold validation

As an orthogonal structure prediction method, we used ESMFold [51] via EsmForProteinFolding from the HuggingFace Transformers library (version 4.46.3) with the esmfold_v1 checkpoint. Both Rebuilt (native) and No-Gln sequences were modeled for all 4,148 proteins. Default parameters were used, except for proteins longer than 780 residues, where chunk_size was set to 128. TMscores were computed against AlphaFoldDB references using USalign with the same parameters as for AlphaFold2 models.

### Natural proteins without glutamine

Using the MMseqs2 easy-rbh pipeline, we searched each of the 6,369 RefSeq prokaryotic proteomes for orthologs (reciprocal best hits) of *E. coli* proteins with E-value ≤ 1e-15. Orthologs lacking glutamine were identified with Python. The same reciprocal-best-hit ortholog sets were then screened for the absence of each amino acid individually: for every amino acid X, we counted the *E. coli* proteins with at least one ortholog containing no X. These per-amino-acid fractions are the points in Fig 7; glutamine is one of the 20. To summarize the relationship between an amino acid’s abundance and the fraction of *E. coli* proteins with an orthologue lacking it (Fig 7, green curve), we fitted the empirical trend across the 20 amino acids with y = a + b·x^−p^, where x is amino-acid abundance and y is the fraction of queries with at least one orthologue lacking that amino acid. The three parameters were estimated together by nonlinear least squares (scipy.optimize.curve_fit), giving a = −0.099, b = 0.013, p = 0.933 (residual sum of squares = 0.153). The fit is descriptive, providing a reference trend against which individual amino acids are compared, and does not represent a null expectation of amino-acid-free orthology.

### Statistical analysis

All statistical analyses were performed in Python 3.11.14 with pandas 2.3.3, NumPy 1.26.4, SciPy 1.16.3, and statsmodels 0.14.6; plotting and the remaining dependencies are listed under “In-house scripts.” Statistics specific to a single analysis are defined where that analysis is introduced (see below); the procedures common to several analyses are collected here.

**Correlation and regression.** Straight-line fits used numpy.polyfit (degree 1) for panel slopes, with the coefficient of determination computed as R² = 1 − SS_res/SS_tot (equal to the squared Pearson correlation for an ordinary-least-squares line, Fig 5A–5C); regressions reported together with a correlation coefficient used scipy.stats.linregress (Figs 1 and 9). Product-moment correlations used numpy.corrcoef or scipy.stats.pearsonr, and rank correlations used scipy.stats.spearmanr — the latter as the primary statistic for the *E. coli*–S. cerevisiae keep-score comparison (Fig 9) and for the supplementary correlations of S5 Fig. Pairwise sequence identity was computed directly, without alignment, because recoded and native sequences are the same length with no gaps.

**Paired comparisons of removal effect.** Per-protein differences in structural score between recoding strategies (Figs 3, 8A and 8B) were tested with the paired Wilcoxon signed-rank test (scipy.stats.wilcoxon). For the proteome-wide glutamine-versus-isoleucine comparison (IQ_full set), the paired t test (scipy.stats.ttest_rel) was additionally applied to TMscore.

**Multiple-testing correction.** Three procedures were applied, matched to the structure of each family of tests. The per-protein BioEmu outlier screen (Fig 6C; see “Conformational Ensembles with BioEmu”) was corrected for false-discovery rate by the Benjamini–Hochberg procedure. The 190 amino-acid-pair Wilcoxon tests of Fig 3 were corrected for family-wise error by the Hochberg step-up procedure, and the three pairwise secondary-structure comparisons (see “Solvent Accessibility and Glutamine Burial”) by the Holm procedure (statsmodels.stats.multitest.multipletests, method holm). We used false-discovery-rate control for the large per-protein screen, where a few false positives among hundreds of tests are acceptable, and family-wise control for the small, structured pairwise families, where any single false claim of difference would be misleading.

**Effect sizes.** For contingency comparisons we report Cramér’s V = √(χ²/[n·min(r − 1, c − 1)]); for the Kruskal–Wallis comparison of keep-propensity across secondary-structure classes we report epsilon-squared = (H − k + 1)/(n − k). Both were computed directly in NumPy.

**Partial correlation (abundance control).** To separate an amino acid’s conservation (keep-score) from its natural abundance as predictors of per-protein removal sensitivity, we computed partial Spearman correlations: each variable was rank-transformed, the two variables of interest were residualized on the control variable by ordinary least squares (numpy.linalg.lstsq), and the residual ranks were correlated. Significance was assessed by a two-sided Freedman–Lane residual permutation test (99,999 permutations), and a 95% confidence interval by resampling proteins with replacement (10,000 bootstrap replicates); both were implemented in NumPy.

**Percentile envelope (**Fig 5C**).** For the ESMFold analysis, the ease-score range [0.15, 1.0] was divided into 50 equal-width bins; within each bin containing at least 12 proteins, the 5th and 95th percentiles of No-Gln TMscore defined a 90% envelope.

**Tests defined with their analyses.** The chi-square, point-biserial, Mann–Whitney, and Spearman tests for glutamine burial and the chi-square/Cramér’s V and Kruskal–Wallis/epsilon-squared tests for secondary structure are described under “Solvent Accessibility and Glutamine Burial”; the Spearman and Pearson correlations for contact order and the one-sided Mann–Whitney and Welch t tests for chain knotting under “Contact Order and Fold Topology”; the descriptive nonlinear-least-squares trend of Fig 7 under “Natural Proteins Without Glutamine”; and the one-sided predictive-t outlier test for the BioEmu ensembles under “Conformational Ensembles with BioEmu.”

### In house scripts

All scripts and figures were generated with Python 3.11 (https://www.python.org/) and its Standard Library, plus several common packages, including biopython 1.86, matplotlib 3.10.7, numba 0.63.1, numpy 1.26.4, pandas 2.3.3, statsmodels 0.14.6, Pillow 12.0.0, scikit-learn 1.7.2, scipy 1.16.3, seaborn 0.13.2, and torch 2.10.0.

### Disclosure: Use of artificial intelligence (AI)

A significant amount of the scripts used in this work were written with the help of ChatGPT (versions 4.1 through 5.4, OpenAI, [52]) either through GitHub Copilot (https://docs.github.com/en) or through Codex CLI (https://developers.openai.com/codex/cli). Claude (Sonnet and Opus, versions 5.5 and 4.6, Anthropic, 2026; https://claude.ai) was used as a writing assistant during manuscript preparation, including drafting and editing of text. All scientific content, analyses, interpretations, and conclusions are the sole responsibility of the authors.

### Design and validation of a glutamine-free β-lactamase (TEM1-Q)

As an experimental consistency check, we designed a TEM-1 β-lactamase variant (UniProt P62593) lacking all glutamine residues in the mature protein while retaining the glutamine in the signal peptide, using ProteinMPNN [39]. The selected design (TEM1-Q) carried eight substitutions: Q37R, Q86E, Q88D, Q97E, Q203R, Q204L, Q265T, and Q274A. The structure was predicted with AlphaFold3 [53] and assessed by pLDDT, RMSD and TMscore relative to the wild-type structure (USalign; [44]), and predicted thermodynamic stability was evaluated with PyRosetta (PyRosetta-4 2025.51, Rosetta Commons [46]) using the same constrained-relaxation protocol applied to the proteome-wide analysis.

### Plasmid construction

The *TEM1-Q* gene was synthesized by Gene Universal Inc. (Newark, DE, USA). It was first cloned into pET-28a(+), whose inducible T7 promoter permits IPTG-driven high-level expression; this construct served only as an expression-insurance intermediate against the possibility of low TEM1-Q activity and provided no data reported here. For a promoter-matched comparison with native TEM-1, the *TEM1-Q* coding sequence was then transferred into pQE-30 (Qiagen), where it exactly replaced the native β-lactamase (*ampR*) coding sequence while preserving the weak constitutive *ampR* promoter. Because the resulting pQE-30–TEM1-Q and pQE-30–native plasmids are identical except for the eight glutamine codons and are expressed from the same promoter, the comparison controls for expression level and construct context.

Subcloning into pQE-30 used NEBuilder HiFi DNA assembly. The pQE-30 backbone (2,600 bp) was linearized by inverse high-fidelity PCR with primers BB-Fwd (5’-ACTCTTCCTTTTTCAATATT-3’) and BB-Rev (5’-CTGTCAGACCAAGTTTACTC-3’) to excise the native *bla* open reading frame (nt 2396–3256). The *TEM1-Q* CDS (861 bp) was amplified from the pET-28a–TEM1-Q template with primers Ins-Fwd (5’-TCAATAATATTGAAAAAGGAAGAGTATGAGCATTCAGCATTTTCG-3’) and Ins-Rev (5’-TATATGAGTAAACTTGGTCTGACAGTTACCAATGTTTAATCAGGC-3’), adding 25-nt terminal overhangs homologous to the linearized backbone to yield a 911 bp amplicon. The backbone PCR was treated with DpnI (10 U, NEB) to remove methylated parental template. Insert and backbone were assembled with NEBuilder HiFi DNA Assembly Master Mix (50 °C, 60 min) and transformed into competent *E. coli* BL21 (DE3), with selection on ampicillin agar. Clones carrying *TEM1-Q* were identified by a BglII site absent from the native *TEM1* CDS, and the resulting construct (3,461 bp) was verified by Sanger sequencing across both flanking junctions.

### Ampicillin-resistance assay

Growth medium for plates and liquid cultures was Luria–Bertani (LB: 10 g/L tryptone, 5 g/L yeast extract, 10 g/L NaCl). Ampicillin resistance was compared between *E. coli* carrying pQE-30–native TEM-1 and pQE-30–TEM1-Q; because both genes are expressed constitutively from the *ampR* promoter, no inducer was used. Overnight cultures were diluted 10^2^- to 10^6^-fold and plated (50 µL per plate) on LB agar containing ampicillin at graded concentrations from 0 to 800 µg/mL. Plates were incubated at 37 °C for 20 h.

### β-lactamase zymography with nitrocefin

β-lactamase activity was detected in-gel following non-reducing SDS-PAGE. Cells were lysed with BugBuster (Novagen, Millipore) supplemented with egg-white lysozyme (1,000 U/mL; Sigma) and Benzonase nuclease (25 U/mL; MilliporeSigma); lysates were centrifuged and the clarified supernatant was used directly, with no further purification. Supernatant was mixed with sample buffer (1/5 volume; 125 mM Tris-HCl pH 6.8, 25% glycerol, 6.25% SDS, 0.05% bromophenol blue) lacking reducing agents and loaded without boiling — 40 µL per lane as the standard load and 20 µL as the half load — onto 12.5% polyacrylamide resolving gels, alongside prestained molecular-weight markers (Thermofisher) and positive and negative controls. Electrophoresis used a standard Tris-glycine system at 4–10 °C. Gels were renatured in 100 mM Tris-HCl (pH 7.0) with 2% (v/v) Triton X-100 for 2 h at room temperature with gentle agitation, then 2 h at 37 °C in fresh buffer, and equilibrated with two 5-min washes in 100 mM potassium phosphate (pH 7.0). Activity was visualized by immersing the gel in freshly prepared 0.5 mM nitrocefin in 100 mM potassium phosphate (pH 7.0) at room temperature, protected from light; active bands appeared rapidly as a pink-to-red hydrolysis product against the yellow substrate and were documented immediately under white light. Total protein was visualized on a duplicate gel stained with 0.2% Coomassie Brilliant Blue (R-250, BioRad), in 10% acetic acid, and 50% methanol.

## Supporting information

S1 Fig Amino acid abundances across prokaryotes. Distribution across 9,720 prokaryotic proteomes; red markers indicate *E. coli*.
(TIF)

S2 Fig Keep-propensity distributions. **(A)** ProteinMPNN. **(B)** MSA. The area under positive values equals the keep-score.
(TIF)

S3 Fig Amino acid composition of optimally recoded proteomes. Native: natural *E. coli* composition. Consensus: most frequent residue per MSA column. MPNN_ideal: highest-probability residue per ProteinMPNN.
(TIF)

S4 Fig Amino acid replacement preferences. Each cell shows the probability that the row amino acid is replaced by the column amino acid (lower-left triangle: MSA; upper-right: ProteinMPNN). Diagonal cells show keep-scores. Horizontally, the 19 off-diagonal values sum to 1.0. Bottom row (“tend”): mean replacement probability per column, scaled 2.5× for visibility.
(TIF)

S5 Fig Crossed agreement of keep scores with amino-acid RMSD ordering. Heatmap of Spearman rank correlations between the median RMSD of each amino-acid variant and either the ProteinMPNN-derived (mpnn_keep) or MSA-derived (msa_keep) keep score. Each cell reports the correlation (ρ) and its two-sided P-value; color intensity encodes ρ (darker teal indicates a stronger positive correlation). Correlations use the 20 standard amino acids, with RMSD summarized as the median across proteins in each set. “MPNN designed” refers to the mut_each_200 set, whereas “MSA designed” refers to the mut_each_300_msa set. The stronger association crosses between the two designs: msa_keep for the MPNN-designed set and mpnn_keep for the MSA-designed set.
(TIF)

S6 Fig Structural accuracy of optimally recoded proteomes. Mean TMscore by ease-score bin (50 equal-sized bins). Each bin contains identical proteins across strategies. Rebuilt: native sequences.
(TIF)

S7 Fig Sequence identity vs. structural similarity in ideal recodings. RMSD to AlphaFoldDB references plotted against sequence identity. Marginal distributions shown above and right.
(TIF)

S8 Fig Flexible alignment rescues low-TMscore models. Flexible TMscore for Rebuilt (x-axis) vs. No-Gln (y-axis) models with rigid No-Gln TMscore < 0.8. Dashed lines: TMscore = 0.8. Only proteins with rigid No-Gln TMscore < 0.8 are included. Of these 548 proteins, 60 had Rebuilt rigid TMscore ≥ 0.8; the remainder had low rigid TMscores for both Rebuilt and No-Gln, reflecting intrinsic flexibility. Marginal histograms show rigid TMscores.
(TIF)

S9 Fig No-Gln models compared to native conformational ensembles. Proteins with rigid No-Gln TMscore < 0.8, sorted by No-Gln TMscore. Blue: No-Gln TMscore. Orange line and shading: mean ± 2 SD of 50 BioEmu samples from native sequences. Black dots: P < 0.05 before multiple-testing correction.
(TIF)

S10 Fig Structural superposition of the two significant outliers. AlphaFoldDB reference (brown), Rebuilt (blue), and No-Gln (pink) for P37619 and P64439—the only proteins with No-Gln TMscore significantly below their BioEmu ensemble after FDR correction (q ≤ 0.05).
(TIF)

S11 Fig Summary of evaluation outcomes for “No‑Gln” ProteinMPNN designs. Models were generated only for the 4148 proteins that passed the length filter (len_ok; 50 < length <= 890). Columns show intersections and their sizes; rows are outcome sets for each test (counts in parentheses). Horizontal blocks group the four tests: rigid alignment, flexible alignment, FoldMason alignment, and BioEmu ensemble statistics. Outcome labels: “NA” (tool failed); “nok” (score below test’s threshold); “ok”, “good”, and “perfect” indicate a properly folded model. Column shading: green indicates support for correct folding (green dots); pink marks a single protein without such support (P75885, natively without glutamines); yellow is neutral on model quality.
(TIF)

S12 Fig A glutamine-free TEM-1 β-lactamase is enzymatically active. Nitrocefin zymogram of native and glutamine-free TEM-1 β-lactamases expressed in *E. coli* BL21 from pQE30. **(A)** β-Lactamase activity detected in situ with nitrocefin. **(B)** Total protein in a parallel gel, loaded with the same samples, visualized with Coomassie. Lanes: M, molecular-weight marker; N, native TEM-1; Q, glutamine-free TEM1-Q; H, untransformed host; P, partially purified mature TEM-1; –, empty lane. Lowercase letters (n, q, h) denote half the corresponding sample load. Native TEM-1 and TEM1-Q give activity bands of comparable intensity that scale with load, whereas the host shows none; neither enzyme is distinguishable above the cellular-protein background in (B). Horizontal bars mark the 25-kDa marker; the arrowhead indicates purified TEM-1 (~28.9 kDa). Samples were loaded unheated and without β-mercaptoethanol to preserve activity, while the marker was reduced and heated, so migration relative to the marker does not reflect molecular mass. Zymogram band intensities **(A)**, normalized to total protein concentration estimated from Coomassie **(B)**, gave these unit-free values: N = 251, n = 124, Q = 300, q = 179.
(TIF)

S13 Fig Buried glutamines are retained more often, but per-position keep-propensity is method-dependent. Native glutamine (Q) positions were classified from DSSP relative solvent accessibility as buried (RSA < 0.20; blue) or exposed (RSA >= 0.20; orange). (A) MSA Q keep fraction: the fraction of reference-Q positions at which Q is the most frequent of the 20 standard amino acids in a homologous-sequence alignment. (B) ProteinMPNN Q keep fraction: the fraction of native-Q positions at which the Q probability is at least that of every other standard amino acid. Points give group fractions and connecting lines are visual guides; the inset values are point-biserial correlations with exposure coded as 1. (C) MSA per-position keep-propensity, calculated after normalizing the 20 amino-acid frequencies within each alignment column as p_Q - max(p_non-Q). (D) ProteinMPNN per-position keep-propensity, calculated as p_Q - max(p_non-Q) from the output probability table. In (C,D), violin width indicates the distribution density, vertical bars indicate the interquartile range, and open circles indicate the median; the dashed line marks zero Q preference. The MSA analysis includes 58,661 Q positions (13,805 buried and 44,856 exposed); the ProteinMPNN analysis includes 59,949 Q positions (14,044 buried and 45,905 exposed). For visual efficiency, violin shapes use a fixed random sample of at most 10,000 positions per group, whereas the displayed medians and interquartile ranges use all positions. Both methods assign Q as retained substantially more often at buried than exposed sites. In contrast, MSA keep-propensity separates strongly across zero, whereas ProteinMPNN keep-propensity is negative in both groups and differs only weakly in rank distribution.
(TIF)

S14 Fig Independent associations of amino-acid median RMSD with MPNN keep score and amino-acid frequency. Partial-Spearman added-variable plots for the mut_each_300_msa set. Median RMSD was calculated across the 300 proteins for each of the 20 amino-acid variants. All variables were rank-transformed; the x- and y-axis values are residuals after linear adjustment for the variable named in each panel. Points are amino acids, solid lines are partial-regression fits, and pale ribbons are 95% confidence bands around the fits. The reported partial-Spearman ρ values are the correlations of the rank residuals. Confidence intervals were obtained by resampling proteins, recalculating amino-acid median RMSD values, and repeating the analysis; P-values are from two-sided Freedman–Lane residual-permutation tests (99,999 permutations). **A)** Median RMSD versus mpnn_keep, after adjusting for amino-acid frequency in mut_each_300_msa. The association remained strong (partial Spearman ρ = 0.741; 95% protein-bootstrap CI [0.585, 0.816]; permutation P = 0.0003). **B)** Median RMSD versus amino-acid frequency in mut_each_300_msa, after adjusting for mpnn_keep. This residual association was weaker and did not reach the 0.05 threshold (partial Spearman ρ = 0.431; 95% protein-bootstrap CI [0.186, 0.620]; permutation P = 0.0647).
(TIF)

S15 Fig Strict positional conservation per amino acid, and its relationship to the MSA keep-score. (**A**) For each amino acid, the percentage of its positions that are invariable — conserved in over 99% of orthologues — as determined from our standard MSAs (built from the “eubacterial” collection of proteomes). (**B**) The same per-amino-acid conserved percentages plotted against the MSA keep-score (the fraction of positions at which the amino acid is the most frequent residue in its alignment column); each point is one amino acid, and the red line is a linear fit (R² = 0.28). The two measures are positively but only weakly associated, because being the most frequent residue in a column and being conserved in over 99% of orthologues are distinct conditions, the latter far stricter. Because conservation above 99% marks positions under strong purifying selection, this stricter measure highlights positions where an amino acid is retained despite its general replaceability — enriching for sites likely to be functionally important (e.g., catalysis, binding, post-translational modification).
(TIF)

S1 Raw Gel Original uncropped images underlying S12 Fig. Panels A and B of S12 Fig derive from two separate gels run in parallel and loaded from the same sample tubes: (A) the in-gel nitrocefin activity assay (zymogram) and (B) the Coomassie-stained total-protein gel. Lane assignments and imaging methods are annotated on each image.
(PDF)

## References

[1] Koonin EV, Novozhilov AS. Origin and evolution of the universal genetic code. Annu Rev Genet. 2017;51:45–62. doi: 10.1146/annurev-genet-120116-024713 28853922

[2] de la Torre D, Chin JW. Reprogramming the genetic code. Nat Rev Genet. 2021;22(3):169–84. doi: 10.1038/s41576-020-00307-7 33318706

[3] Tamames J, Gil R, Latorre A, Peretó J, Silva FJ, Moya A. The frontier between cell and organelle: Genome analysis of Candidatus Carsonella ruddii. BMC Evol Biol. 2007;7:181. doi: 10.1186/1471-2148-7-181 17908294 PMC2175510

[4] Bohlin J, Brynildsrud O, Vesth T, Skjerve E, Ussery DW. Amino acid usage is asymmetrically biased in AT- and GC-rich microbial genomes. PLoS One. 2013;8:e69878. doi: 10.1371/journal.pone.0069878 23922837 PMC3724673

[5] Liu L, Rochereau C, Kozlov S, Urtecho G, Liu X, Zhao J, et al. Toward life with a 19-amino acid alphabet through generative artificial intelligence design. Science. 2026;392(6797):eaeb5171. doi: 10.1126/science.aeb5171 42060756

[6] Shibue R, Sasamoto T, Shimada M, Zhang B, Yamagishi A, Akanuma S. Comprehensive reduction of amino acid set in a protein suggests the importance of prebiotic amino acids for stable proteins. Sci Rep. 2018;8(1):1227. doi: 10.1038/s41598-018-19561-1 29352156 PMC5775292

[7] Yagi S, Padhi AK, Vucinic J, Barbe S, Schiex T, Nakagawa R, et al. Seven amino acid types suffice to create the core fold of RNA polymerase. J Am Chem Soc. 2021;143(39):15998–6006. doi: 10.1021/jacs.1c05367 34559526

[8] Makarov M, Meng J, Tretyachenko V, Srb P, Březinová A, Giacobelli VG, et al. Enzyme catalysis prior to aromatic residues: Reverse engineering of a dephospho-CoA kinase. Protein Sci. 2021;30(5):1022–34. doi: 10.1002/pro.4068 33739538 PMC8040869

[9] Longo LM, Lee J, Blaber M. Simplified protein design biased for prebiotic amino acids yields a foldable, halophilic protein. Proc Natl Acad Sci U S A. 2013;110(6):2135–9. doi: 10.1073/pnas.1219530110 23341608 PMC3568330

[10] Rovner AJ, Haimovich AD, Katz SR, Li Z, Grome MW, Gassaway BM, et al. Recoded organisms engineered to depend on synthetic amino acids. Nature. 2015;518(7537):89–93. doi: 10.1038/nature14095 25607356 PMC4590768

[11] Torres L, Krüger A, Csibra E, Gianni E, Pinheiro VB. Synthetic biology approaches to biological containment: Pre-emptively tackling potential risks. Essays Biochem. 2016;60(4):393–410. doi: 10.1042/EBC20160013 27903826 PMC5264511

[12] Jones DT, Taylor WR, Thornton JM. The rapid generation of mutation data matrices from protein sequences. Comput Appl Biosci. 1992;8(3):275–82. doi: 10.1093/bioinformatics/8.3.275 1633570

[13] Henikoff S, Henikoff JG. Amino acid substitution matrices from protein blocks. Proc Natl Acad Sci U S A. 1992;89(22):10915–9. doi: 10.1073/pnas.89.22.10915 1438297 PMC50453

[14] Yampolsky LY, Stoltzfus A. The exchangeability of amino acids in proteins. Genetics. 2005;170(4):1459–72. doi: 10.1534/genetics.104.039107 15944362 PMC1449787

[15] Kyte J, Doolittle RF. A simple method for displaying the hydropathic character of a protein. J Mol Biol. 1982;157(1):105–32. doi: 10.1016/0022-2836(82)90515-0 7108955

[16] Murphy LR, Wallqvist A, Levy RM. Simplified amino acid alphabets for protein fold recognition and implications for folding. Protein Eng. 2000;13(3):149–52. doi: 10.1093/protein/13.3.149 10775656

[17] Takabatake K, Izawa K, Akikawa M, Yanagisawa K, Ohue M, Akiyama Y. Improved large-scale homology search by two-step seed search using multiple reduced amino acid alphabets. Genes (Basel). 2021;12(9):1455. doi: 10.3390/genes12091455 34573438 PMC8469100

[18] Solis AD. Amino acid alphabet reduction preserves fold information contained in contact interactions in proteins. Proteins. 2015;83(12):2198–216. doi: 10.1002/prot.24936 26407535

[19] Halpern AL, Bruno WJ. Evolutionary distances for protein-coding sequences: Modeling site-specific residue frequencies. Mol Biol Evol. 1998;15(7):910–7. doi: 10.1093/oxfordjournals.molbev.a025995 9656490

[20] Drummond DA, Wilke CO. Mistranslation-induced protein misfolding as a dominant constraint on coding-sequence evolution. Cell. 2008;134(2):341–52. doi: 10.1016/j.cell.2008.05.042 18662548 PMC2696314

[21] Zou Z, Zhang J. Amino acid exchangeabilities vary across the tree of life. Sci Adv. 2019;5(12):eaax3124. doi: 10.1126/sciadv.aax3124 31840062 PMC6892623

[22] Jumper J, Evans R, Pritzel A, Green T, Figurnov M, Ronneberger O, et al. Highly accurate protein structure prediction with AlphaFold. Nature. 2021;596(7873):583–9. doi: 10.1038/s41586-021-03819-2 34265844 PMC8371605

[23] Tunyasuvunakool K, Adler J, Wu Z, Green T, Zielinski M, Žídek A, et al. Highly accurate protein structure prediction for the human proteome. Nature. 2021;596(7873):590–6. doi: 10.1038/s41586-021-03828-1 34293799 PMC8387240

[24] Dauparas J, Anishchenko I, Bennett N, Bai H, Ragotte RJ, Milles LF, et al. Robust deep learning-based protein sequence design using ProteinMPNN. Science. 2022;378(6615):49–56. doi: 10.1126/science.add2187 36108050 PMC9997061

[25] Giacobelli VG, Andersson S, Srb P, Neuwirthová T, Ruszová Z, Marhoul J, et al. Ancient amino acid sets enable stable protein folds. bioRxiv. 2025:2025.10.29.685319. doi: 10.1101/2025.10.29.685319

[26] Mirdita M, Schütze K, Moriwaki Y, Heo L, Ovchinnikov S, Steinegger M. ColabFold: Making protein folding accessible to all. Nat Methods. 2022;19(6):679–82. doi: 10.1038/s41592-022-01488-1 35637307 PMC9184281

[27] Varadi M, Anyango S, Deshpande M, Nair S, Natassia C, Yordanova G, et al. AlphaFold Protein Structure Database: Massively expanding the structural coverage of protein-sequence space with high-accuracy models. Nucleic Acids Res. 2022;50(D1):D439–44. doi: 10.1093/nar/gkab1061 34791371 PMC8728224

[28] Ieremie I, Ewing RM, Niranjan M. Protein language models meet reduced amino acid alphabets. Bioinformatics. 2024;40(2):btae061. doi: 10.1093/bioinformatics/btae061 38310333 PMC10872054

[29] Todd AE, Orengo CA, Thornton JM. Evolution of function in protein superfamilies, from a structural perspective. J Mol Biol. 2001;307(4):1113–43. doi: 10.1006/jmbi.2001.4513 11286560

[30] DePristo MA, Weinreich DM, Hartl DL. Missense meanderings in sequence space: A biophysical view of protein evolution. Nat Rev Genet. 2005;6(9):678–87. doi: 10.1038/nrg1672 16074985

[31] Fiser A, Simon I, Barton GJ. Conservation of amino acids in multiple alignments: Aspartic acid has unexpected conservation. FEBS Lett. 1996;397(2–3):225–9. doi: 10.1016/s0014-5793(96)01181-7 8955352

[32] Perlinska AP, Sikora M, Sulkowska JI. Everything AlphaFold tells us about protein knots. J Mol Biol. 2024;436(19):168715. doi: 10.1016/j.jmb.2024.168715 39029890

[33] Trifonov EN. Consensus temporal order of amino acids and evolution of the triplet code. Gene. 2000;261(1):139–51. doi: 10.1016/s0378-1119(00)00476-5 11164045

[34] Especial JNC, Faísca PFN. Folding and knotting of biotic and prebiotic amino acid sequences through reverse evolution. Biophys J. 2025;124(17):2877–87. doi: 10.1016/j.bpj.2025.07.030 40734278 PMC12461004

[35] Especial JNC, Faísca PFN. Topological enhancement of protein kinetic stability. Phys Rev E. 2026;113(6):L062402. doi: 10.1103/qqyf-18y8 42493997

[36] Fleming J, Magana P, Nair S, Tsenkov M, Bertoni D, Pidruchna I, et al. AlphaFold protein structure database and 3D-Beacons: New data and capabilities. J Mol Biol. 2025;437(15):168967. doi: 10.1016/j.jmb.2025.168967 40133787

[37] Steinegger M, Söding J. MMseqs2 enables sensitive protein sequence searching for the analysis of massive data sets. Nat Biotechnol. 2017;35(11):1026–8. doi: 10.1038/nbt.3988 29035372

[38] Edgar RC. MUSCLE: Multiple sequence alignment with high accuracy and high throughput. Nucleic Acids Res. 2004;32(5):1792–7. doi: 10.1093/nar/gkh340 15034147 PMC390337

[39] Dauparas J, Lee GR, Pecoraro R, An L, Anishchenko I, Glasscock C, et al. Atomic context-conditioned protein sequence design using LigandMPNN. Nat Methods. 2025;22(4):717–23. doi: 10.1038/s41592-025-02626-1 40155723 PMC11978504

[40] Kabsch W, Sander C. Dictionary of protein secondary structure: Pattern recognition of hydrogen-bonded and geometrical features. Biopolymers. 1983;22(12):2577–637. doi: 10.1002/bip.360221211 6667333

[41] Tien MZ, Meyer AG, Sydykova DK, Spielman SJ, Wilke CO. Maximum allowed solvent accessibilites of residues in proteins. PLoS One. 2013;8(11):e80635. doi: 10.1371/journal.pone.0080635 24278298 PMC3836772

[42] Plaxco KW, Simons KT, Baker D. Contact order, transition state placement and the refolding rates of single domain proteins. J Mol Biol. 1998;277(4):985–94. doi: 10.1006/jmbi.1998.1645 9545386

[43] Dorier J, Goundaroulis D, Benedetti F, Stasiak A. Knoto-ID: A tool to study the entanglement of open protein chains using the concept of knotoids. Bioinformatics. 2018;34(19):3402–4. doi: 10.1093/bioinformatics/bty365 29722808

[44] Zhang C, Shine M, Pyle AM, Zhang Y. US-align: Universal structure alignments of proteins, nucleic acids, and macromolecular complexes. Nat Methods. 2022;19(9):1109–15. doi: 10.1038/s41592-022-01585-1 36038728

[45] Xu J, Zhang Y. How significant is a protein structure similarity with TM-score = 0.5? Bioinformatics. 2010;26(7):889–95. doi: 10.1093/bioinformatics/btq066 20164152 PMC2913670

[46] Chaudhury S, Lyskov S, Gray JJ. PyRosetta: A script-based interface for implementing molecular modeling algorithms using Rosetta. Bioinformatics. 2010;26(5):689–91. doi: 10.1093/bioinformatics/btq007 20061306 PMC2828115

[47] Tyka MD, Keedy DA, André I, Dimaio F, Song Y, Richardson DC, et al. Alternate states of proteins revealed by detailed energy landscape mapping. J Mol Biol. 2011;405(2):607–18. doi: 10.1016/j.jmb.2010.11.008 21073878 PMC3046547

[48] Alford RF, Leaver-Fay A, Jeliazkov JR, O’Meara MJ, DiMaio FP, Park H, et al. The Rosetta all-atom energy function for macromolecular modeling and design. J Chem Theory Comput. 2017;13(6):3031–48. doi: 10.1021/acs.jctc.7b00125 28430426 PMC5717763

[49] Gilchrist CLM, Mirdita M, Steinegger M. Multiple protein structure alignment at scale with FoldMason. Science. 2026;391(6784):485–8. doi: 10.1126/science.ads6733 41610233

[50] Lewis S, Hempel T, Jiménez-Luna J, Gastegger M, Xie Y, Foong AYK, et al. Scalable emulation of protein equilibrium ensembles with generative deep learning. Science. 2025;389(6761):eadv9817. doi: 10.1126/science.adv9817 40638710

[51] Lin Z, Akin H, Rao R, Hie B, Zhu Z, Lu W, et al. Evolutionary-scale prediction of atomic-level protein structure with a language model. Science. 2023;379(6637):1123–30. doi: 10.1126/science.ade2574 36927031

[52] Chen M, Tworek J, Jun H, Yuan Q, de Oliveira Pinto HP, Kaplan J, et al. Evaluating large language models trained on code. arXiv [cs.LG]. 2021. doi: 10.48550/arXiv.2107.03374

[53] Abramson J, Adler J, Dunger J, Evans R, Green T, Pritzel A, et al. Accurate structure prediction of biomolecular interactions with AlphaFold 3. Nature. 2024;630(8016):493–500. doi: 10.1038/s41586-024-07487-w 38718835 PMC11168924

