# Supplementary material for: Folding without glutamine: Proteome-scale removal of a canonical amino acid
Source: PLoS Comput Biol. 2026 Nov 3;22(11):e1014931. doi: 10.1371/journal.pcbi.1014931 (PMC13743030; doi:10.1371/journal.pcbi.1014931)
Supplement: Supplementary file 1 [file pcbi.1014931.s016.pdf]

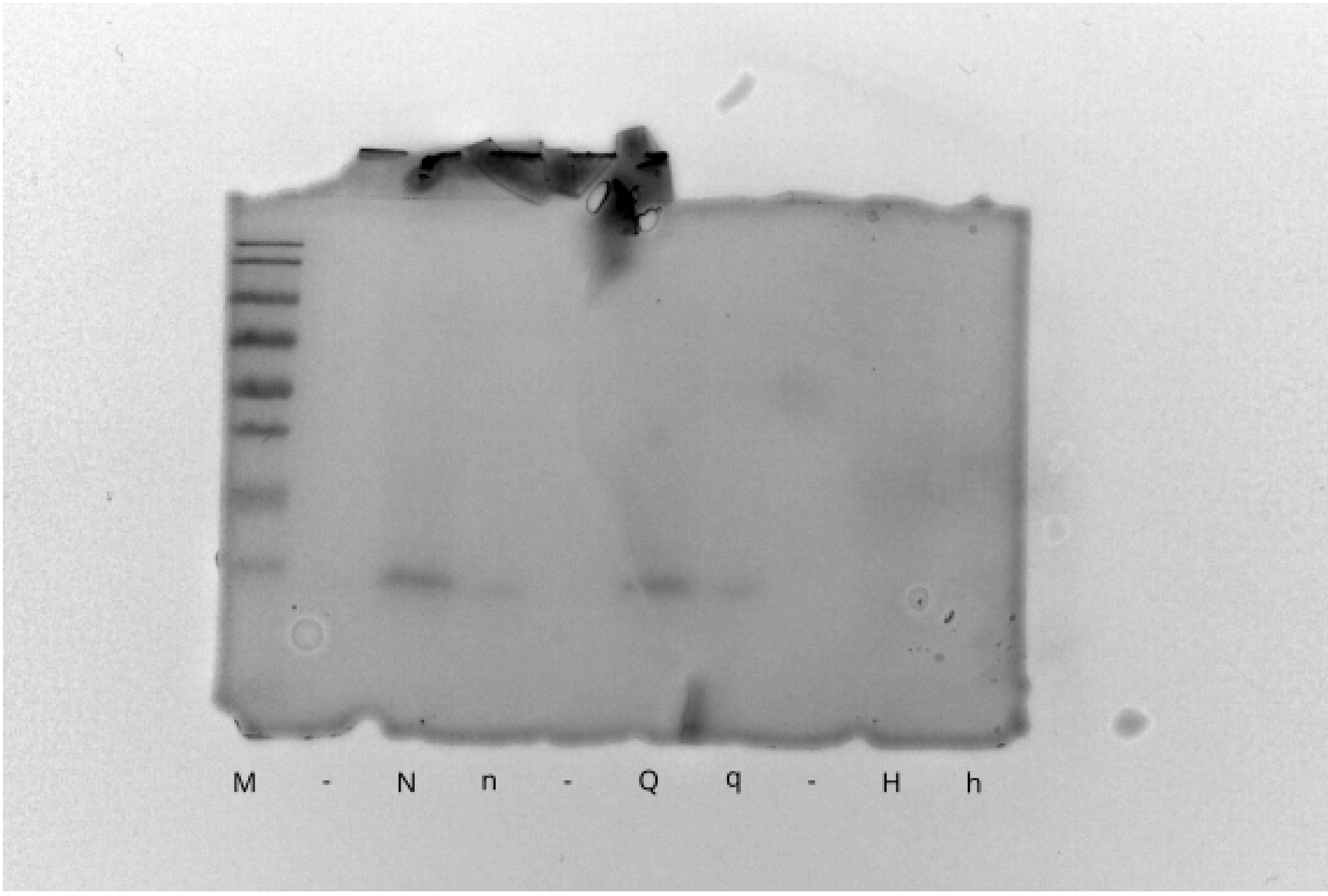

Original uncropped image underlying Fig S12A. Nitrocefin in-gel activity assay (zymogram) of native and glutamine-free TEM-1 β-lactamase expressed in *E. coli* BL21 from pQE30. This gel and the Coomassie-stained gel in Fig S12B are two separate gels, run in parallel and loaded from the same sample tubes. Lanes: M, molecular-weight marker; N, native TEM-1; n, half-load native; Q, glutamine-free TEM1-Q; q, half-load TEM1-Q; H, untransformed host; P, partially purified mature TEM-1; –, empty lane. Detection: hydrolyzed nitrocefin, visible-light photograph.

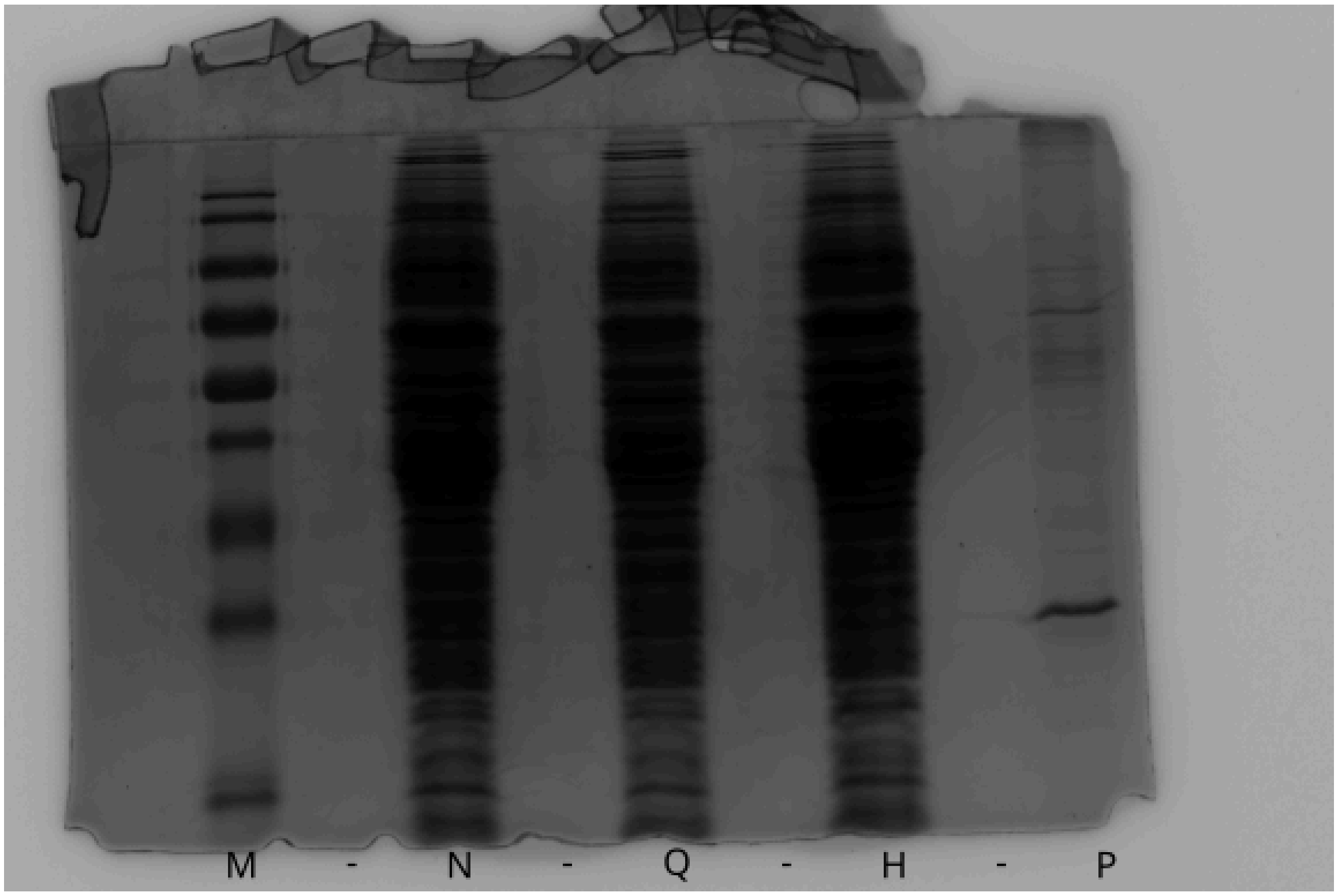

Original uncropped image underlying Fig S12B. Coomassie R-250 total-protein stain of a gel run in parallel with the zymogram in Fig S12A and loaded from the same sample tubes. Lanes: M, molecular-weight marker; N, native TEM-1; Q, glutamine-free TEM1-Q; H, untransformed host; P, partially purified mature TEM-1; –, empty lane. Detection: Coomassie R-250 stain, visible-light photograph.
