# Supplementary material for: Folding without glutamine: Proteome-scale removal of a canonical amino acid
Source: PLoS Comput Biol. 2026 Nov 3;22(11):e1014931. doi: 10.1371/journal.pcbi.1014931 (PMC13743030; doi:10.1371/journal.pcbi.1014931)
Supplement: Supplementary file 2 [file pcbi.1014931.s018.docx]

# Response to Reviewer #1

We thank Reviewer #1 for recognizing the originality and scope of the work and for a set of comments that converge on a single, well-taken theme: that the strength of our claims must be matched to the limitations of a computational framework, and that structural tolerance must be kept distinct from biological dispensability throughout. We have taken this seriously and made it a guiding principle of the revision — reframing the central claim, softening conclusions consistently, adding a dedicated scope paragraph, expanding the treatment of circularity, and adding sensitivity analyses. We address each point below.

**R1-1 (structural tolerance vs biological dispensability).** *"The manuscript repeatedly extends structural predictions into broader biological claims of 'dispensability' and 'proteome-wide fold preservation.' In reality, the analyses primarily demonstrate compatibility with current computational structure prediction models rather than experimentally validated biological function. The distinction… should be stated more clearly and consistently throughout, and several conclusions should be softened accordingly."*

We agree and have made this distinction explicit and consistent. The study is now framed throughout as a computational analysis of structural replaceability, not a demonstration of biological dispensability — beginning with the title, from which we have removed the word "dispensability" (the subtitle now reads "proteome-scale removal of a canonical amino acid"), and continuing in the abstract's closing sentence, the author summary, the Introduction's close, and the Discussion opener. We retired the strongest formulations: the Discussion no longer opens by calling glutamine "the most structurally dispensable canonical amino acid," and the phrase 'confirming its status as the most structurally dispensable amino acid' has been removed. Figure 3 itself has been rebuilt (in response to Reviewer #4): it is now an RMSD-based significance analysis (paired Wilcoxon signed-rank tests, Hochberg-corrected) across 300 matched proteins rather than a display of near-identical TMscore distributions, and the softened claim reflects what this significance analysis actually supports. In their place we now report the more careful pair of findings that the data actually support: glutamine has the lowest keep-score under both methods, and its removal is among the least structurally perturbing across the proteome — significantly less than that of every higher-keep residue, yet statistically indistinguishable from the other low-keep residues (Figure 3). The Discussion now states explicitly that fold preservation is necessary but not sufficient for function: a protein that folds without glutamine may still be catalytically inactive or unable to participate in essential interactions if glutamine is functionally required, a distinction that only genome-wide experimental testing can resolve.

Changes: central claim reframed across abstract, author summary, Introduction, and Discussion; "most structurally dispensable" language removed; dedicated structural-vs-biological scope paragraph added. (Title, line 1; Abstract, final sentence; Author Summary, lines ~34–48; Introduction close, lines ~161–164; Discussion opener, lines ~533–537; scope paragraph, lines ~742–747.)

**R1-2 (circularity and reliance on predicted references).** *"A second important limitation is the heavy reliance on AlphaFoldDB structures as reference 'ground truth.' Since these are themselves computational predictions, the study largely compares predicted models against other predicted models… the manuscript would benefit from a more explicit discussion of this inherent circularity and its implications."*

We have expanded the discussion of circularity into a dedicated paragraph that addresses both the design-and-evaluate concern and the predicted-reference concern the reviewer raises. Three features of the analysis argue that the result is not merely an artifact of comparing predictions to predictions. First, the framework discriminates: removing isoleucine, a higher-keep residue, is measurably more disruptive than removing glutamine across the same proteome and pipeline (Figure 8), and glycine — a positive control — is more disruptive still; a pipeline that only reproduced shared training bias could not order these residues as their keep-scores predict. Second, the two keep-scores are methodologically independent — MSA conservation uses no structural model and shares no training data with ProteinMPNN or AlphaFold2, yet converges on the same ranking (Figure 1). Third, and directly addressing the predicted-reference concern, our baseline is an independent reproducibility metric rather than model self-confidence: the ease-score is the TMscore of the native rebuild against its AlphaFoldDB reference, computed without pLDDT or PAE, so it normalizes precisely for the predicted-versus-predicted issue by asking only whether a redesigned sequence reproduces its own reference as faithfully as the native sequence does. We state plainly that the AlphaFoldDB references are themselves predictions and that ease-score normalization, while controlling for prediction variability, does not eliminate this dependency.

Changes: circularity expanded into a dedicated Discussion paragraph (discrimination, method independence, ease-score as a reproducibility baseline); the predicted-reference dependency stated explicitly in the limitations paragraph.

**R1-3 (limited experimental validation).** *"The experimental validation remains very limited in scope. The TEM-1 β-lactamase experiment is a useful and valuable consistency check, but a single enzyme cannot support broad claims about proteome-wide amino acid elimination. This should be explicitly acknowledged, and the conclusions restricted accordingly."*

We fully agree and have restricted the conclusions accordingly. The TEM1-Q experiment is now presented explicitly as a single consistency check, not a validation of the proteome-wide analysis. The Discussion states that it does not validate the proteome-wide result — which would require systematic experimental testing of essential proteins, as Liu et al. performed for isoleucine in the ribosome — and that what it does show is more modest: that the computational pipeline produces predictions consistent with biological function in at least one well-characterized case, and that glutamine removal is compatible with the full chain of events required for a periplasmic enzyme (translation, secretion, signal-peptide processing, and catalysis). We have also substantially strengthened the single data point itself. In the revised experiment (detailed in response to Reviewer #4), TEM1-Q was re-cloned into a promoter-matched vector in which it exactly replaces the native β-lactamase, so that it and native TEM-1 differ only by the eight glutamine substitutions and are expressed identically. Under this exact comparison the two enzymes are indistinguishable — both confer resistance up to 400 µg/mL ampicillin and neither at 800 — and a matched-loading in-gel activity assay shows comparable activity for both. The glutamine-free enzyme is therefore not a weakened variant but one native-like in both resistance and in-gel activity. Even so, we are careful not to let this one case carry proteome-wide claims.

Changes: TEM1-Q framed as a single consistency check with its scope explicitly limited; the enzyme experiment strengthened (pQE-30 re-clone, matched in-gel activity assay) without over-extending its interpretation.

**R1-4 (methodological justification: threshold, ensemble statistics, MSA robustness).** *"Several methodological choices require clarification or justification, including the TMscore ≥ 0.8 threshold, the statistical treatment of ensemble data, and the robustness of MSA construction parameters. The manuscript would be strengthened by sensitivity analyses showing that the main conclusions are stable under reasonable alternative assumptions."*

We addressed all three.

*Threshold.* We now justify the TMscore ≥ 0.8 cutoff explicitly in Methods. A TMscore above 0.5 already indicates a shared fold [45]; we adopt the stricter 0.8 so that "structurally correct" denotes close backbone agreement rather than merely shared topology. The choice is also calibrated to our own data: 86% of native rebuilds exceed 0.8 against their AlphaFoldDB references (the ease-score; Figure 2A), and sub-threshold proteins have systematically high PAE (r = −0.81; Figure 2B), indicating that low scores reflect intrinsic flexibility rather than misfolding. Where finer discrimination than this saturating metric allows is needed, we use RMSD instead (Figures 3, 8).

*Ensemble statistics.* The treatment of ensemble and per-protein data is now specified in a consolidated Statistical Analysis section. The BioEmu outlier screen tests each protein's single No-Gln TMscore as a one-sided outlier against its native conformational ensemble (predictive t-test, n − 1 df) with Benjamini–Hochberg FDR correction across proteins; the amino-acid-pair comparisons of Figures 3 and 8 use paired Wilcoxon signed-rank tests with Hochberg family-wise correction. We explain the rationale for using FDR control on the large per-protein screen and family-wise control on the small structured pairwise families.

*MSA robustness.* To test whether the MSA-derived keep-scores depend on the alignment-construction settings, we recomputed them under substantially altered parameters and compared the results to the originals across all 20 amino acids (2,000 proteins). Two changes were applied simultaneously: the non-redundant homologue collection was rebuilt at 90% maximum identity rather than 95%, and the alignment-depth decimation was removed entirely, retaining all homologues up to the MMseqs2 default of 420 per query. The per-amino-acid keep-scores were essentially unchanged (linear fit slope = 0.97, intercept = 0.03, Pearson r = 0.98; Spearman ρ = 0.95, n = 20, P = 1.1 × 10⁻¹⁰), and glutamine remained the lowest-keep residue with proline and glycine the highest. The central ranking is therefore stable under reasonable alternative alignment-construction assumptions. This analysis is reported here in response to the reviewer rather than added to the manuscript.

Changes: TMscore threshold justified in Methods (Xu & Zhang fold-significance value plus ease-score calibration); statistical procedures consolidated and rationalized in the Statistical Analysis section. The MSA-parameter sensitivity analysis is reported in this letter (not added to the manuscript), per the note above.

**R1-5 (cautious interpretation when moving to evolutionary/biological generalization).** *"While the keep-score framework and multi-method convergence are interesting and potentially useful, the manuscript would benefit from more cautious interpretation, especially when moving from computational structural compatibility to evolutionary or biological generalization."*

We have tempered these transitions. Where the manuscript moves from structural compatibility to evolutionary or biological statements, it now does so with explicit caveats. The evolutionary-ortholog result is described as showing that glutamine-free orthologs are more common than abundance would predict — evidence that glutamine is unusually dispensable in natural proteomes — without claiming it establishes biological dispensability. The cross-species keep-score conservation (Figure 9) is interpreted as indicating that keep-score reflects intrinsic properties of protein architecture rather than the composition of any single proteome, explicitly qualified as being "from ProteinMPNN's perspective." And a new limitations paragraph, added in response to Reviewer #3, notes that preservation of the native structural state does not establish preservation of folding kinetics, cooperativity, or kinetic stability, citing Faísca and co-workers on the topology-dependence of alphabet-reduction effects — so the leap from a preserved fold to a functioning, foldable protein is now explicitly bounded.

Changes: evolutionary and cross-species interpretations qualified; kinetics/cooperativity caveat added to the limitations paragraph (with Faísca references), bounding the generalization from structural compatibility to biological viability.

# Response to Reviewer #2

We thank Reviewer #2 for the concise and constructive assessment. The two points raised — testing whether higher-keep amino acids behave differently, and making the analysis code available — are both well taken. The first is now answered directly by a new analysis added to the manuscript (removing isoleucine and glycine across the same proteome), and the second by depositing the full pipeline in a public repository. We address each below.

**R2-1 (test amino acids with higher keep-scores).** *"The central claim rests on glutamine having the lowest keep-score, but the manuscript does not show what happens for amino acids with higher keep-scores. Without such a comparison it is difficult to know whether the pipeline is genuinely sensitive to the choice of amino acid, or whether it would report almost any single amino acid as removable. Applying the same procedure to one or more higher-keep-score residues would establish that the framework discriminates."*

We agree that this is the necessary control, and we have added it. Using the same proteome and the same pipeline, we removed isoleucine (a mid-range keep-score residue) and glycine (a high-keep-score residue, serving as a positive control) and compared the structural impact to glutamine removal, at two scales.

On 571 proteins with MSA-consensus replacement, structural disruption increased in the order glutamine < isoleucine << glycine, with all three pairwise differences significant (paired Wilcoxon signed-rank test; P = 1.7 × 10⁻¹¹ for glutamine versus isoleucine, and P < 10⁻⁵¹ for both comparisons against glycine); glutamine removal was the least disruptive of the three in 65% of proteins (Figure 8A). Across the full proteome with ProteinMPNN-optimal replacement, glutamine and isoleucine are nearly indistinguishable by TMscore — an expected consequence of ProteinMPNN reproducing the native backbone whatever residue is removed — but by the more sensitive RMSD metric glutamine removal remains the less disruptive (lower RMSD in 57.4% of proteins; median ΔRMSD = −0.024 Å; paired Wilcoxon P = 1.1 × 10⁻¹⁵), and this advantage increases monotonically with ease-score, emerging most clearly where AlphaFold2 is most reliable (Figure 8B).

The framework therefore does not report every amino acid as equally removable: it orders residues as their keep-scores predict, with glycine strongly disruptive, isoleucine intermediate, and glutamine least. This directly answers the reviewer's concern that the pipeline might be indiscriminately tolerant, and it is the same discrimination result we now use to address the circularity question raised by Reviewers #1 and #4.

Changes: new "Keep-score discriminates" Results subsection and Figure 8 (isoleucine and glycine removed by the same pipeline, at two scales, with significance); the corresponding Discussion text presents this as a completed discrimination control. (Results subsection "Keep-score discriminates: isoleucine is harder to remove than glutamine," lines ~418–460; Fig 8 and caption, lines ~462–479. Corresponding Discussion text at lines ~639–655.)

**R2-2 (code availability).** *"The data availability statement indicates that analysis scripts are available on request. For a study of this kind, whose conclusions depend entirely on a custom computational pipeline, the code should be publicly deposited so that the analysis can be reproduced and scrutinized. I have marked data availability as 'No' for this reason."*

We agree without reservation and have deposited the code publicly. All in-house scripts implementing the pipeline — flexible alignment, keep-score and keep-propensity calculation, significance testing, contact-order and knot analysis, and figure generation — together with the sequence and structural-score data underlying the study, are now deposited at Zenodo (DOI: 10.5281/zenodo.22558702) and the Data availability statement has been rewritten to point there, replacing the previous "available on request" language. The deposit is self-contained, so that the full analysis can be reproduced and scrutinized independently, as the reviewer requests.

Changes: analysis pipeline and underlying data deposited at Zenodo (DOI: 10.5281/zenodo.22558702); Data availability statement rewritten from "available on request" to a public-repository citation.

# Response to Reviewer #3

We thank Reviewer #3 for the exceptionally generous assessment and for identifying the mechanistic question — *why* glutamine is so replaceable — as the most interesting biological problem raised by our study. This prompted the analysis we consider the most important new addition to the revision: partitioning every reference glutamine by its local structural environment, we now show, directly in the *E. coli* proteome, that the glutamines conservation and design retain are the buried, structurally constrained minority, whereas the replaceable majority sit at exposed, low-constraint positions. This converts what was previously a literature inference into an in-proteome result and, together with the topology analyses the reviewer suggested and the Faísca kinetic-stability caveat, is reflected throughout the revised Discussion and Methods. We address each point below.

**R3-1.** *"ProteinMPNN was trained on naturally occurring proteins that contain glutamine… it would be useful to discuss the extent to which ProteinMPNN probabilities remain reliable when evaluating sequence spaces subject to constraints that were not represented during training, such as a completely glutamine-free proteome. Have the authors assessed whether the model remains properly calibrated when one amino acid type is systematically excluded from the design space?"*

We appreciate this point and have clarified an aspect of the pipeline that we think resolves the central concern, while adding an explicit caveat about a residual limitation the reviewer's question rightly implies.

First, the pipeline never asks ProteinMPNN to operate on a sequence space it did not see in training. ProteinMPNN is only ever applied to **native, glutamine-containing backbones evaluated in native sequence context** (--single_aa_score 1 --use_sequence 1); at each position it returns an ordinary 20-way probability distribution, and we select the highest-probability non-glutamine residue. This is an in-distribution prediction: at no point is a glutamine-free protein passed back to ProteinMPNN to be scored or designed. The recoded, glutamine-free sequences are handed only to the *folding* evaluators (AlphaFold2, and orthogonally ESMFold, Rosetta, FoldMason, BioEmu), never to the model that generated them. The out-of-distribution scenario the reviewer describes — scoring within a systematically glutamine-free design space — therefore does not arise in our procedure, and we now state this explicitly where the ProteinMPNN scoring is introduced.

Second, the residual question — whether the top-ranked non-glutamine choice is itself trustworthy — is addressed not by relying on the model's internal calibration but by convergent, ProteinMPNN-independent evidence, which we now foreground: (i) MSA conservation, which shares no training data or architecture with ProteinMPNN or AlphaFold2, independently assigns glutamine the lowest keep-score and independently identifies proline and glycine as least replaceable (Figure 1); (ii) the framework discriminates among residues in the order their keep-scores predict — removing isoleucine is measurably more disruptive than removing glutamine, and glycine (positive control) more disruptive still (Figure 8) — which a systematically miscalibrated model could not do; and (iii) the resulting glutamine-free folds are corroborated by four evaluators that do not inherit ProteinMPNN's biases (Figures 5, 6). Even were ProteinMPNN's per-position probabilities imperfectly calibrated, the downstream conclusion is anchored by tools that are independent of it.

Third — and this is the caveat the reviewer's question implies — because ProteinMPNN's per-position probabilities are conditioned on the surrounding sequence, our choice of each glutamine replacement from the *native* background takes the marginal optimum at each position independently and does not re-optimize jointly across the co-substituted sites. We therefore do not model second-order (epistatic) coupling among the removed glutamines. We now note this limitation explicitly, together with its mitigation: the structural evaluation is performed on the **fully recoded** sequence, so any destabilization arising from coupling among the simultaneous substitutions would be registered by AlphaFold2, ESMFold, Rosetta, and BioEmu — and none is detected. This is consistent with the contrast to Liu et al. [5], whose isoleucine removals frequently required *compensatory* mutations at structural-neighbor positions (epistasis made explicit), whereas single top-choice glutamine replacement, without compensatory changes, preserved native folds in 85.5% of proteins — as expected if glutamine tends to occupy the less-constrained, less-coupled positions.

Changes: Methods ("ProteinMPNN Probability Tables") now states that scoring is performed only on native backbones in native context and never on glutamine-free inputs; the Discussion circularity paragraph now includes the calibration argument (MSA independence, discrimination, orthogonal validation); and the limitations paragraph now carries the epistasis caveat and its mitigation. (Methods "ProteinMPNN Probability Tables," lines ~858–873; Discussion circularity paragraph, lines ~656–679; limitations/epistasis paragraph, lines ~766–778.)

**R3-2.** *"The manuscript convincingly demonstrates that glutamine is highly replaceable, but the structural reasons for this remain unclear… are retained glutamines preferentially associated with buried sites, ligand-binding pockets, protein-protein interfaces, or particular secondary-structure elements? Understanding why glutamine is unusually replaceable would significantly strengthen the impact of the work."*

We fully agree, and this is the analysis we were most glad to add. We partitioned every reference glutamine by relative solvent accessibility (DSSP; buried = RSA < 0.20) and by DSSP secondary-structure class, and compared retention between the two methods. Burial is the dominant determinant: glutamine is the most conserved residue in its alignment column at 68% of buried positions versus 42% of exposed ones, and ProteinMPNN likewise retains it more often when buried (28% versus 9%; Figure S13). By MSA conservation the per-position keep-propensity favoring glutamine is positive at buried positions and essentially absent at exposed ones (median +0.35 versus −0.05). Secondary structure shows the same directionality far more weakly (helices/strands retained slightly more than coil; Cramér's V = 0.0824), explaining little of the variance. We report the ProteinMPNN result honestly: its continuous keep-propensity remains negative for both groups, so buried glutamines are retained more *often* without being more *strongly* favored by every metric.

This per-position finding has an independent, pre-AI precedent that we now cite as convergent support: Fiser et al. [31] reported that glutamine is the least conserved of all twenty amino acids and that conservation declines with solvent accessibility, with glutamine falling below even what its high exposure would predict — reached decades earlier, from alignments alone, without any structure-prediction model. Together these establish that whether a glutamine is retained is governed chiefly by its local structural environment — principally burial — rather than by any intrinsic property of the residue, and provide a common root for the agreement of our two keep-scores (an exposed glutamine is both structurally unconstrained, which ProteinMPNN registers, and weakly conserved, which the alignments register).

Changes: new Methods section "Solvent Accessibility and Glutamine Burial" (DSSP RSA, Tien et al. maximum-accessibility normalization, statistical tests); new Figure S13; new Discussion paragraph on the burial mechanism and the Fiser precedent. Per our scope decision, we frame the mechanism in structural terms (exposure/constraint) and keep it behind the ProteinMPNN geometry-only firewall; we do not invoke codon or metabolic explanations, which would concern *conservation* non-structurally and are not needed for the structural claim.

**R3-3.** *"It remains unclear whether glutamine dispensability is uniform across proteins with different structural architectures. Are proteins with complex topologies (e.g. knotted), large contact order, or non-trivial folding mechanisms more sensitive to glutamine removal than the proteome average?"*

We added two topology analyses and reconcile them explicitly. First, relative contact order (RCO, following Plaxco et al. [42]) predicts neither which glutamines are retained (Spearman ρ = −0.002 for keep-score, 0.010 for keep-propensity; both non-significant) nor how well a fold tolerates their removal (ρ = −0.044; P = 0.009, n = 3,581 — significant but negligible, and if anything toward *greater* tolerance). Global topological complexity therefore imposes no penalty on glutamine removal. Second, as an extreme discrete feature we classified chain knotting with Knoto-ID [43] on the well-ordered set (Rebuilt PAE < 5 Å; 9 knotted of 1,589). We frame the knot result cautiously and as double-edged, because it is: glutamine removal in knotted chains did not measurably increase ΔRMSD relative to unknotted chains (one-sided Mann–Whitney P = 0.383), but knotted chains do carry higher glutamine keep-propensity (median 0.67 vs 0.50; keep-propensity 0.36 vs 0.10; P = 0.075 and 0.012). We therefore report that knotting, where detectable, tracks greater glutamine *retention* rather than reduced tolerance to removal — and, with only nine knotted chains, that any such effect is tentative and underpowered. The two analyses are consistent once one notes that a knot is a discrete topological feature not captured by the continuous contact-order metric; we say so explicitly so the apparent tension is not mistaken for a contradiction. The overall conclusion is that tolerance of glutamine removal is governed by *local* structural environment (burial, R3-2) rather than global fold complexity.

Changes: new Methods section "Contact Order and Fold Topology" (RCO definition; Knoto-ID classification; directional tests), lines ~924–961; new Discussion paragraph reconciling the contact-order null with the knot hint, lines ~625–638. These analyses are reported in the text and Methods; no dedicated SI figure is included.

**R3-4.** *"The manuscript would benefit from a broader discussion of previous studies examining how reductions in amino-acid diversity affect protein folding and the extent to which these effects depend on protein architecture… Faísca and co-workers (Biophysical Journal, 2025) … a follow-up study (Physical Review E, 2026) … reductions in amino-acid diversity may have important consequences for kinetic stability even when native-state structures appear largely preserved."*

We thank the reviewer for these references, which sharpen an important limitation, and have added both along with a dedicated caveat. Our readout evaluates whether reduced-alphabet sequences are compatible with the native *structural state*, not the *kinetics* by which that state is reached; preservation of the predicted native fold does not establish preservation of folding rates, cooperativity, kinetic stability, or avoidance of off-pathway intermediates. We now cite Faísca and co-workers on exactly this point: using alphabet size as a proxy for evolutionary time, they found the folding consequences of a reduced alphabet to be strongly topology-dependent — unknotted and shallowly knotted proteins largely retained two-state cooperativity under an ancestral ten-amino-acid alphabet, whereas a deeply knotted protein had its cooperativity and folding ability significantly compromised [34]; and, in a companion structure-based study, that kinetic stability rises with knot depth and that enlarging the alphabet predominantly enhances kinetic stability [35]. Native-state structure can thus appear preserved while kinetic and cooperative properties are not — a caveat that applies directly to conclusions, such as ours, drawn from structural and stability metrics alone. This dovetails with our own knot observation (R3-3): the special status of knotted topologies in their work is consistent with our finding that knotted chains retain glutamine more.

Changes: two references added [34,35]; new limitations paragraph on the structure-vs-kinetics distinction, explicitly tied to topology and to our knot result.

# Response to Reviewer #4

We thank Reviewer #4 for the careful, item-by-item reading and for pushing hardest on the experimental interpretation, which prompted the single largest new addition to the revision. Following the reviewer's recommendation (M5, M6), we re-cloned the glutamine-free enzyme into a promoter-matched vector (pQE-30) in which the *TEM1-Q* coding sequence exactly replaces the native β-lactamase, removing the His-tag and the accidental upstream-ATG artifact, and we added a matched-loading in-gel activity assay against native TEM-1. This one construct resolves the vector, His-tag, and gel-legibility concerns together (M5, M6, m8) and puts the whole-cell resistance comparison on an exact, expression-controlled footing. We address each point below.

## Major Comments

**R4-M1.** *"The manuscript repeatedly extends structural predictions into broader biological claims of 'dispensability' and 'proteome-wide fold preservation'… The distinction between structural tolerance and true biological dispensability should be stated more clearly and consistently throughout, and several conclusions should be softened accordingly."*

We agree and have reframed the central claim throughout. The study is now explicitly presented as a computational analysis of structural replaceability, not a demonstration of biological dispensability — this is stated in the revised title (from which "dispensability" has been removed), the abstract's closing sentence ("a computational analysis of structural replaceability, not a demonstration of biological dispensability"), the author summary, the Introduction's closing, and the Discussion opener, which no longer claims glutamine is "the most structurally dispensable" but that an entire amino acid can be removed without large structural consequences and that keep-score identifies glutamine a priori as the best single target. Correspondingly, we retired the phrase "confirming its status as the most structurally dispensable amino acid." The underlying analysis was itself rebuilt (Figure 3 is now an RMSD-based significance test; see R4-M3), and it shows glutamine to be significantly less disruptive to remove than every higher-keep residue yet statistically indistinguishable from the other low-keep residues. Accordingly, we describe glutamine as lying firmly among the least impactful residues and, by virtue of its lowest keep-score, the best a priori target — not as uniquely dispensable. A dedicated scope paragraph in the Discussion states that fold preservation is necessary but not sufficient for function, and that glutamine at active sites, interfaces, or modification sites may remain functionally essential even where the surrounding fold is maintained. We further verified that the main ranking does not depend on these alignment-construction choices: recomputing the keep-scores with the non-redundant collection rebuilt at 90% identity and with the depth decimation removed (all homologues retained up to the MMseqs2 default) leaves them essentially unchanged (Pearson r = 0.98; Spearman ρ = 0.95, P = 1.1 × 10⁻¹⁰), with glutamine lowest and proline and glycine highest — reported in full in our response to point R1-4

Changes: title/abstract/author-summary/Introduction/Discussion reframed; "most structurally dispensable" language removed; scope paragraph added. (Title, line 1; Abstract, final sentence; Author Summary, lines ~34–48; Introduction close, lines ~161–164; Discussion opener, lines ~533–537; scope paragraph, lines ~742–747.)

**R4-M2 (circularity, and the Discussion opener).** *"There is a circular logic in the validation pipeline… a high TM-score might simply show that they share the same training biases… While the authors comment on this partly in the discussion, it did not prevent them from (excessive) claims, such as 'The central finding of this study is that glutamine is the most structurally dispensable canonical amino acid…' (discussion opening)."*

We have both softened the opener (R4-M1) and strengthened the circularity discussion into a dedicated paragraph. The opener no longer asserts that glutamine is "the most structurally dispensable canonical amino acid." The circularity paragraph now makes three arguments that the result is not merely shared training bias. First, the framework discriminates: removing isoleucine — a higher-keep-score residue — is measurably more disruptive than removing glutamine across the same proteome and pipeline (Figure 8), and glycine, a positive control, is more disruptive still; a pipeline reproducing only shared bias could not order these residues as their keep-scores predict. Second, the two keep-scores are methodologically independent: MSA conservation uses no structural model and shares no training data with ProteinMPNN or AlphaFold2, yet converges on the same ranking (Figure 1), and sequences designed by one criterion are predicted as replaceable by the other. Third, our baseline is an independent reproducibility metric, not model self-confidence — the ease-score is the TMscore of the native rebuild against its AlphaFoldDB reference, computed without pLDDT or PAE, so it normalizes for the predicted-versus-predicted concern by asking only whether a redesigned sequence reproduces its own reference as faithfully as the native does. We also concede honestly that, because ProteinMPNN reproduces the native backbone whatever residue is removed, it folds an isoleucine-free proteome nearly as well as a glutamine-free one by TMscore, which is why we resolve the per-residue difference with the more sensitive RMSD metric and with MSA-guided designs that do not optimize for structure.

Changes: Discussion opener softened; circularity expanded into a dedicated paragraph (discrimination, method independence, ease-score as reproducibility baseline, honest concession on TMscore compression).

**R4-M3 (Figure 3 significance).** *"'Removing Gln caused the least disruption, confirming its status as the most structurally dispensable amino acid.' The problems… (1) sample of 200 proteins is too small (2) the difference between the displayed TMscore distributions are infinitesimal. Are the differences statistically significant? — no such metric is reported."*

We have rebuilt this analysis. Figure 3 is now based on 300 matched high-ease proteins (the mut_each_300_msa set) rather than 200, and reports RMSD rather than TMscore because, for these high-ease proteins, TMscore saturates near 1.0 and cannot resolve small per-amino-acid differences whereas RMSD remains sensitive — a rationale we now state explicitly, which also addresses the "infinitesimal differences" concern (the differences were compressed by a saturating metric, not absent). Significance is now reported for every pair: each off-diagonal cell is a two-sided paired Wilcoxon signed-rank test, Hochberg-corrected across all 190 amino-acid pairs. Of the 134 pairwise differences significant after correction, 126 agree with the keep-score ranking and 8 oppose it. Glutamine removal is significantly less disruptive than that of eleven higher-keep residues and never significantly more disruptive than any residue, though statistically indistinguishable from the other low-keep residues — which is why we now describe it as among the least impactful rather than uniquely least.

Changes: Figure 3 redrawn as an RMSD significance heatmap (paired Wilcoxon, Hochberg-corrected); n increased to 300; TMscore-saturation rationale stated; claim softened.

**R4-M4 (isoleucine as necessary validation, not future work).** *"The whole pipeline… is interpreted as 'the glutamine-free proteome is structurally equivalent to the native', but is it really an intrinsic feature of Q-free proteome?… I believe [removing Ile] is not an informative future extension but a necessary validation that needs to be added into this study."*

We agree, and the isoleucine comparison is now part of the study rather than proposed future work. We removed isoleucine across the same proteome with the same pipeline used for glutamine, at two scales. On 571 proteins with MSA-consensus replacement (the QIG_571_msa set), structural impact increased in the order Q < I << G, all three pairwise differences significant (paired Wilcoxon P = 1.7 × 10⁻¹¹ for Q vs I; P < 10⁻⁵¹ for both comparisons against the glycine positive control), with glutamine removal less disruptive than isoleucine removal in 65% of proteins (Figure 8A). Across the full proteome with ProteinMPNN-optimal replacement (the IQ_full set), the two are nearly indistinguishable by the saturating TMscore metric — an expected consequence of the design method — but by RMSD, glutamine removal remains the less disruptive of the two (lower RMSD in 57.4% of proteins; median ΔRMSD = −0.024 Å; paired Wilcoxon P = 1.1 × 10⁻¹⁵), and this advantage rises monotonically with ease-score, emerging most clearly where AlphaFold2 is most reliable (Figure 8B). The framework therefore does not tolerate any substitution uniformly; it orders residues as their keep-scores predict, which is the direct answer to whether the glutamine-free result reflects a property of glutamine rather than of the design tool.

Changes: new "Keep-score discriminates" Results subsection and Figure 8 (QIG_571_msa and IQ_full sets); the corresponding Discussion text now presents isoleucine removal as completed validation, not future work.

**R4-M5 (WT TEM-1 titration).** *"TEM1-Q confers resistance up to 50 µg/mL without induction and up to 600 µg/mL with IPTG… a severe loss of activity compared to wild-type TEM-1 which confers resistance to thousands of µg/mL. A similar titration should be done on cells carrying the wild-type variant… Even better would be growth-rate analysis or an in vitro activity assay."*

We addressed this by making the comparison exact rather than comparing to a literature value. The IPTG-induced pET-28a construct that gave the 50/600 µg/mL numbers served only as an expression-insurance intermediate and reported no data in the revised manuscript. For the resistance comparison we transferred *TEM1-Q* into pQE-30, where it exactly replaces the native β-lactamase coding sequence under the same weak constitutive *ampR* promoter, and compared it head-to-head with the pQE-30–native construct. Because the two plasmids are identical except for the eight glutamine codons and are expressed from the same promoter, the comparison controls for expression level and construct context. Both grew at up to 400 µg/mL ampicillin and neither at 800 µg/mL — i.e. native TEM-1 and TEM1-Q are indistinguishable in this matched assay, and the apparent "severe loss" relative to a literature figure reflected the different (non-native, un-induced) expression context of the earlier construct, not an intrinsic deficit of the glutamine-free enzyme. We further added the reviewer's suggested activity assay: a nitrocefin in-gel zymogram of soluble extracts at matched loading (Figure S12), in which native TEM-1 and TEM1-Q produce activity bands of comparable intensity that scale with load, while the untransformed host shows none.

Changes: pQE-30 re-clone replaces the pET-28a data for the resistance comparison; matched-promoter titration (both cap at 400 µg/mL) and nitrocefin zymogram (Figure S12) added; TEM1-Q Results and Discussion rewritten accordingly.

**R4-M6 (His-tag / vector choice / re-clone).** *"S13 reveals two bands for TEM1-Q… an unprocessed enzyme initiating at an upstream accidental NcoI ATG that adds a 20-residue His-tag extension, burying the signal peptide and preventing translocation… The authors should explain why they used pET-28a(+)… Even better would be to reclone TEM1-Q into a vector without His-tag."*

We took the recommended route and re-cloned. In pQE-30 the *TEM1-Q* coding sequence exactly replaces the native *ampR* gene with no His-tag and no upstream accidental ATG, so only the mature, correctly processed enzyme is expressed and the two-band artifact is eliminated. The pET-28a construct is now described in Methods solely as an expression-insurance intermediate that provided none of the reported data, which explains its original use while making clear it is not the basis of any conclusion. Because pQE-30–TEM1-Q and pQE-30–native differ only by the eight glutamine codons, the whole-cell resistance and in-gel activity comparisons are now made between constructs that are otherwise identical.The SDS-PAGE that revealed the artifact (former Figure S13) is retired accordingly; the pQE-30 constructs are now documented by the nitrocefin in-gel activity assay in the new Figure S12 (see R4-m8).

Changes: TEM1-Q re-cloned into pQE-30 (Methods "Plasmid construction" rewritten with the assembly details); pET-28a reduced to an expression-insurance intermediate; His-tag/accidental-ATG artifact removed. The SDS-PAGE that revealed the artifact (former Figure S13) is retired accordingly, and the pQE-30 constructs are now documented by the nitrocefin in-gel activity assay in the new Figure S12 (see R4-m8).

## Minor Comments

**R4-m1 (reduced-alphabet references).** *"The authors might wish to include a more comprehensive set of key references on proteins previously constructed from reduced alphabets, next to Shibue et al. 2018… e.g. pnas.1219530110; molbev msac032; jacs.1c05367; pro.4068."*

We thank the reviewer. All four are directly relevant reduced-alphabet demonstrations and are already cited in the manuscript: Yagi et al. (ref 7), Makarov et al. (ref 8), and Longo et al. (ref 9) in the Introduction's survey of single-protein alphabet reduction (lines ~65–68), and Giacobelli et al. (ref 25) in the Introduction and Discussion where inverse-folding design from restricted alphabets is discussed (line ~112 and lines ~714–716).

Changes: four reduced-alphabet references added to the Introduction's alphabet-reduction survey.

**R4-m2 (Fig 1 regression equation and statistics).** *"Missing linear regression equation and statistical analysis in Fig1."*

Added. The Figure 1 caption now reports the regression line (y = 1.50 x − 0.53), Pearson r = 0.78, and P = 5.2 × 10⁻⁵, and the Methods "Statistical Analysis" section specifies that this fit used scipy.stats.linregress.

Changes: regression equation and statistics added to the Figure 1 caption; method specified.

**R4-m3 (compare No-Q to Rebuilt rather than AfDB).** *"If authors did rebuild the AF2 models, why didn't they use them for TM-score comparison… the comparison will be more accurate."*

Much of the manuscript already rests on exactly this logic. The ease-score normalization uses each protein's own in-house Rebuilt model as its control, and the three validation panels of Figure 5 (local pLDDT, Rosetta energetics, ESMFold) as well as the contact-order and knot analyses are all direct, paired No-Gln↔Rebuilt comparisons — precisely to remove the cross-method (AfDB-version) confound the reviewer raises. We report the population TMscore against the common AlphaFoldDB reference for both the No-Gln and the Rebuilt series because that shared reference is what makes them comparable to each other on one axis; a direct No-Gln↔Rebuilt TMscore would be ambiguous, since neither model is ground truth and any difference could not be attributed to one rather than the other. The same-settings confound is instead controlled by the ease-score and the paired Figure 5 analyses.

Changes: none required (addressed by argument); the ease-score and paired Figure 5 comparisons already implement the reviewer's intent.

**R4-m4 (Fig 4 color/legend mismatch).** *"Switched color labels in Fig4 — discrepancy between captions and color legend in the plot."*

Corrected in the revised Figure 4; the legend colors now match the caption assignments (Rebuilt, ProteinMPNN No-Gln, MSA No-Gln).

Changes: Figure 4 color legend corrected.

**R4-m5 (explain the ease-score).** *"Better explanation and introduction of an 'ease-score' is missing."*

The ease-score is now defined where it is first used in Results (as each protein's Rebuilt TMscore against its AlphaFoldDB reference, quantifying how reliably AlphaFold2 reproduces that structure and controlling for intrinsic flexibility) and again in a dedicated Methods subsection ("Ease-score and Squared Mean PAE"). Figure 2 and its caption present the ease-score distribution and its correlation with PAE, making explicit that low ease-scores reflect flexibility rather than prediction failure.

Changes: ease-score defined at first use in Results and in a dedicated Methods subsection; Figure 2 illustrates it.

**R4-m6 (weights, seeds, temperatures).** *"Confirm that the exact model weights, random seeds, and temperature parameters (e.g. T = 0.1 or 0.3 in ProteinMPNN) are explicitly detailed in Methods… Applies to almost all tools."*

We have specified these throughout the Methods. For sequence scoring we used LigandMPNN v1.5.5 with --model_type 'ligand_mpnn' --seed 111 --single_aa_score 1 --use_sequence 1 --batch_size 1 --number_of_batches 10 at the software default temperature of 0.1, with per-position probabilities taken as the average over the 10 scoring runs; the fixed seed makes this reproducible while the averaging accounts for stochastic decoding order. Structure prediction used LocalColabFold with default parameters (five models per sequence, colabfold_batch <a3m> <outdir>; no seed or temperature overridden), and ESMFold via HuggingFace Transformers 4.46.3 with the esmfold_v1 checkpoint. Rosetta calculations used PyRosetta-4 2025.51 with the ref2015 energy function and the FastRelax protocol under Cα coordinate constraints (harmonic, σ = 1.0 Å). Versions for USalign, MMseqs2, MUSCLE, DSSP, Knoto-ID, BioEmu, and FoldMason are given in their respective Methods subsections.

Changes: Methods "ProteinMPNN Probability Tables" corrected to ligand_mpnn, seed 111, temperature 0.1; tool versions and parameters stated across the Methods.

**R4-m7 (Fig 1 keep-score vs S11 conserved-positions discrepancy).** *"The keep-score should heavily depend on conservation of the given residue at a given position. How can the authors explain the discrepancy between calculated keep-scores and percentage of conserved positions shown in S11?"*

There is no discrepancy; the two quantities measure different things, and this difference is intentional rather than a problem to be reconciled. We now make the distinction explicit, explain why the analysis was included, and provide the visible comparison the reviewer asked for.

The three metrics used in this work are complementary by design. The ProteinMPNN keep-score reflects structural constraint alone; the MSA keep-score (Figure 1, x-axis) is the fraction of positions at which the native residue is the single most frequent (modal, plurality-optimal) residue in its alignment column, and so summarizes the evolutionary behavior of glutamines in bulk; and the fraction of positions conserved in over 99% of orthologues is a deliberately strict filter that isolates the small minority of glutamines retained across evolution *despite* low structural constraint. Being modal is a far weaker condition than being conserved in >99% of homologues, so for every residue the keep-score exceeds the strict-conservation fraction, and the two need not agree in level even where they agree in rank — this is expected, not contradictory. For glutamine both keep-scores are low and consistently so — rarely even modal, and strictly conserved at only ~7% of positions.

The purpose of the >99%-conservation analysis is precisely to recognize that not all glutamines are dispensable even when they are not structurally required: a glutamine can be retained for reasons beyond fold stability — catalysis, binding, post-translational modification, or regulation. The strict-conservation filter enriches for exactly this functionally essential subset, and identifying it complements, rather than contradicts, the two keep-scores. We now state this rationale explicitly in the Discussion, where we note that these three measures are complementary by design and that the ~7% of glutamine positions conserved in over 99% of orthologues may mark functionally critical sites worthy of targeted investigation. Per the reviewer's request for a visible comparison, we present the composite Figure S15, whose panels show the per-amino-acid >99%-conserved fraction and a per-amino-acid scatter of keep-score against that conserved fraction.

Changes: definitional paragraph added distinguishing modal (keep-score) from strict conservation; Figure S15 made a composite that includes a per-amino-acid keep-score-vs-conservation scatter.

**R4-m8 (S13b gel unreadable).** *"The SDS-PAGE gel in S13b is very hard to read… the authors should rework the figure."*

The gel has been reworked. The nitrocefin in-gel activity assay from the pQE-30 constructs replaces the previous S13; the new Figure S12 shows β-lactamase activity detected in situ with nitrocefin (panel A) and total protein by coomassie (panel B), with native TEM-1, glutamine-free TEM1-Q, untransformed host, and half-load lanes, and legible activity bands that scale with load.

Changes: previous S13 replaced by the pQE-30 nitrocefin zymogram (Figure S12).
