# Supplementary material for: Folding without glutamine: Proteome-scale removal of a canonical amino acid
Source: PLoS Comput Biol. 2026 Nov 3;22(11):e1014931. doi: 10.1371/journal.pcbi.1014931 (PMC13743030; doi:10.1371/journal.pcbi.1014931)
Supplement: Peer review history

# Author comment: Author response to Decision Letter 0

**DOI:** 10.1371/journal.pcbi.1014931.r001

**Transfer Alert**

This paper was transferred from another journal. As a result, its full editorial history (including decision letters, peer reviews and author responses) may not be present.

8 Jun 2026

# Peer review: Decision Letter 0

**DOI:** 10.1371/journal.pcbi.1014931.r002

**Authors:** Arne Elofsson, Arli Aditya Parikesit

29 Jun 2026

PCOMPBIOL-D-26-01364

Folding without glutamine: proteome-scale analysis of amino acid dispensability.

PLOS Computational Biology

Dear Dr. Garciarrubio,

Thank you for submitting your manuscript to PLOS Computational Biology. After careful consideration, we feel that it has merit but does not fully meet PLOS Computational Biology's publication criteria as it currently stands. Therefore, we invite you to submit a revised version of the manuscript that addresses the points raised during the review process.

Please submit your revised manuscript by Aug 28 2026 11:59PM. If you will need more time than this to complete your revisions, please reply to this message or contact the journal office at ploscompbiol@plos.org. When you're ready to submit your revision, log on to https://www.editorialmanager.com/pcompbiol/ and select the 'Submissions Needing Revision' folder to locate your manuscript file.

Please include the following items when submitting your revised manuscript:

* A letter that responds to each point raised by the editor and reviewer(s). You should upload this letter as a separate file labeled 'Response to Reviewers'. This file does not need to include responses to formatting updates and technical items listed in the 'Journal Requirements' section below.

* A marked-up copy of your manuscript that highlights changes made to the original version. You should upload this as a separate file labeled 'Revised Manuscript with Track Changes'.

* An unmarked version of your revised paper without tracked changes. You should upload this as a separate file labeled 'Manuscript'.

If you would like to make changes to your financial disclosure, competing interests statement, or data availability statement, please make these updates within the submission form at the time of resubmission. Guidelines for resubmitting your figure files are available below the reviewer comments at the end of this letter. As the corresponding author, your ORCID iD is verified in the submission system and will appear in the published article. PLOS supports the use of ORCID, and we encourage all coauthors to register for an ORCID iD and use it as well. Please encourage your coauthors to verify their ORCID iD within the submission system before final acceptance, as unverified ORCID iDs will not appear in the published article. Only the individual author can complete the verification step; PLOS staff cannot verify ORCID iDs on behalf of authors. We look forward to receiving your revised manuscript.

Kind regards,

Arli Aditya Parikesit, PhD

Academic Editor

PLOS Computational Biology

Arne Elofsson

Section Editor

PLOS Computational Biology

**Additional Editor Comments (if provided):**

It is clear as per reviewers' comment that extensive revision is necessary. Please kindly do the needful and provide the letter of reply to address the comments.

Kindly check If the reviewer is providing the commented manuscript for follow ups

Duly note that the suggested reference citations from the reviewers are not mandatory to be incorporated, if they are irrelevant with your manuscript.

**Journal Requirements:**

If the reviewer comments include a recommendation to cite specific previously published works, please review and evaluate these publications to determine whether they are relevant and should be cited. There is no requirement to cite these works unless the editor has indicated otherwise.

1) We ask that a manuscript source file is provided at Revision. Please upload your manuscript file as a .doc, .docx, .rtf or .tex. If you are providing a .tex file, please upload it under the item type u2018LaTeX Source Fileu2019 and leave your .pdf version as the item type u2018Manuscriptu2019.

2) We do not publish any copyright or trademark symbols that usually accompany proprietary names, eg ©,  ®, or TM  (e.g. next to drug or reagent names). Therefore please remove all instances of trademark/copyright symbols throughout the text, including:

- TM on page: 48.

3) Please upload all main figures as separate Figure files in .tif or .eps format. For more information about how to convert and format your figure files please see our guidelines:

https://journals.plos.org/ploscompbiol/s/figures

4) Please upload a copy of Figure Fig 1-8 which you refer to in your text on pages 17, 19, 21, 22, 24, and 25. Or, if the figure is no longer to be included as part of the submission please remove all reference to it within the text.

5) We have noticed that you have uploaded Supporting Information files, but you have not included a list of legends. Please add a full list of legends for your Supporting Information files after the references list.

**Reviewers' comments:**

Reviewer's Responses to Questions

**Comments to the Authors:**

**Please note here if the review is uploaded as an attachment.**

Reviewer #1: This is a highly original and technically ambitious manuscript addressing an important question in protein evolution and synthetic biology: which amino acids are most structurally replaceable at proteome scale. The combination of ProteinMPNN-based inverse folding, MSA conservation, AlphaFold2/ESMFold structural evaluation, and evolutionary ortholog analysis is impressive, and the central observation that glutamine consistently emerges as the most replaceable residue is interesting and potentially impactful.

However, several key issues need to be addressed before the conclusions can be fully supported.

1. The main concern is that the manuscript repeatedly extends structural predictions into broader biological claims of “dispensability” and “proteome-wide fold preservation.” In reality, the analyses primarily demonstrate compatibility with current computational structure prediction models rather than experimentally validated biological function. The distinction between structural tolerance and true biological dispensability should be stated more clearly and consistently throughout the manuscript, and several conclusions should be softened accordingly.

2. A second important limitation is the heavy reliance on AlphaFoldDB structures as reference “ground truth.” Since these are themselves computational predictions, the study largely compares predicted models against other predicted models. While the authors partially mitigate this with multiple orthogonal tools (Rosetta, ESMFold, FoldMason, BioEmu), the manuscript would benefit from a more explicit discussion of this inherent circularity and its implications for interpretation.

3. In addition, the experimental validation remains very limited in scope. The TEM-1 β-lactamase experiment is a useful and valuable consistency check, but a single enzyme cannot support broad claims about proteome-wide amino acid elimination. This should be explicitly acknowledged, and the conclusions should be restricted accordingly.

4. There are also several methodological choices that require clarification or justification, including the TMscore ≥ 0.8 threshold, the statistical treatment of ensemble data, and the robustness of MSA construction parameters. The manuscript would be strengthened by sensitivity analyses showing that the main conclusions are stable under reasonable alternative assumptions.

5. Finally, while the keep-score framework and multi-method convergence are interesting and potentially useful contributions, the manuscript would benefit from more cautious interpretation, especially when moving from computational structural compatibility to evolutionary or biological generalization.

Overall, the study is novel, large-scale, and methodologically rich, and the central finding regarding glutamine is intriguing. However, substantial revision is needed to better align the strength of the claims with the limitations of the computational framework.

Reviewer #2: The authors used existing computational tools to evaluate structural dispensability of amino acids in the E. coli proteome. The paper shows substantial computational evidence that Glutamine has highest dispensability. A functional validation was also done on one of the proteins.

My main concern is the following. Structural dispensability of Gluatmine is predominanlty shown by how replacing it perturbs the "actual" fold the least. This correlates with the "keep score". Folds were determined by AF2. Although authors acknowledge the variability of AF2 predictions, it would make the claim of the paper much stronger if the authors can pick another higher "keep score" amino acid and repeated the AF2 predictions. For example, how do figures 4 and 5 look for this alternative experiment?

Nevertheless, I think the built pipeline has potential to explore dispensability of amino acids in other proteomes as well. Regarding this, can authors please provide open access to their homegrown scripts?

Reviewer #3: This manuscript addresses a fundamental and long-standing question in molecular evolution and protein biophysics: to what extent is the canonical set of twenty amino acids necessary for the structure and function of contemporary proteomes? Building on recent experimental demonstrations that individual amino acids can be eliminated from the translational machinery of Escherichia coli, the authors develop an ambitious computational framework to assess amino-acid replaceability at proteome scale. By combining structure-based probabilities derived from ProteinMPNN with evolutionary conservation inferred from multiple sequence alignments, they introduce a "keep-score" metric and identify glutamine as the amino acid most amenable to replacement, whereas glycine and proline emerge as the least replaceable. The convergence of two independent methodologies on the same ranking constitutes a major strength of the study and lends credibility to the central conclusion.

I thoroughly enjoyed reading this manuscript and believe that it is suitable for publication in PLOS Computational Biology. The work is original, technically sophisticated, and addresses an important question with potentially broad implications for protein evolution, amino-acid alphabet reduction, and synthetic biology. The results are compelling and, in my view, warrant publication. Nevertheless, I would like the authors to address the following points in a revised version of the manuscript.

1. The central conclusions of the manuscript rely heavily on ProteinMPNN predictions. However, ProteinMPNN was trained on naturally occurring proteins that contain glutamine. It would therefore be useful to discuss the extent to which ProteinMPNN probabilities remain reliable when evaluating sequence spaces subject to constraints that were not represented during training, such as a completely glutamine-free proteome. Have the authors assessed whether the model remains properly calibrated when one amino acid type is systematically excluded from the design space?

2. The manuscript convincingly demonstrates that glutamine is highly replaceable, but the structural reasons for this remain unclear. In my view, this is one of the most interesting biological questions raised by the study. A comparative characterization of replaceable and non-replaceable glutamines could provide valuable mechanistic insight. For example, are retained glutamines preferentially associated with buried sites, ligand-binding pockets, protein-protein interfaces, or particular secondary-structure elements? Understanding why glutamine is unusually replaceable would significantly strengthen the impact of the work.

3. The manuscript presents a global ranking of amino-acid replaceability across the proteome. However, it remains unclear whether glutamine dispensability is uniform across proteins with different structural architectures. Are proteins with complex topologies (e.g. knotted), large contact order, or non-trivial folding mechanisms more sensitive to glutamine removal than the proteome average? Addressing this question could reveal whether amino-acid replaceability depends not only on local structural context but also on the complexity of the underlying folding landscape.

Finally, the manuscript would benefit from a broader discussion of previous studies examining how reductions in amino-acid diversity affect protein folding and the extent to which these effects depend on protein architecture. In particular, Faísca and co-workers (Biophysical Journal, 2025, DOI: 10.1016/j.bpj.2025.07.030.) showed that the effects of amino-acid alphabet reduction are strongly dependent on protein topology. While unknotted and shallowly knotted proteins retained cooperative folding under reduced amino-acid alphabets, a deeply knotted protein required substantially greater sequence heterogeneity to preserve efficient folding and knot formation. These results suggest that the consequences of amino-acid reduction are highly context-dependent and may vary considerably among different structural classes of proteins. In a follow-up study (Physical Review E, 2026, DOI: https://doi.org/10.1103/qqyf-18y8) they investigated the evolutionary consequences of increasing amino-acid alphabet complexity and found that the incorporation of biotic amino acids has a clear effect on kinetic stability which incrasses substantiallywith the incorporation of biotic amino acids. These results suggest that reductions in amino-acid diversity may have important consequences for kinetic stability even when native-state structures appear largely preserved. This observation is particularly relevant in the context of the present work, where the conclusions are primarily based on structural predictions and stability-related metrics.

Reviewer #4: This manuscript addresses a foundational question in evolutionary biology and protein engineering: to what extent is the canonical 20-amino-acid alphabet structurally redundant at a proteome-wide scale? Combining computational workflows (ProteinMPNN structure-based probabilities and Multiple Sequence Alignment conservation) across the Escherichia coli proteome, the authors introduce a metric called the 'keep-score' to evaluate amino acid replaceability, which we believe is interesting point of view on the introduced problem. The manuscript is well-written, and conceptually innovative. However, several critical items in experimental interpretation, machine learning circularity, and evolutionary framing should be addressed before its publication.

Major Comments:

1. One of the two methods used for "keep-score" calculation is based on MSA analyses. This type of analysis primarily depends on how well the database of analyzed homologs is built. In this manuscript, the study that the authors reference for comparison (Liu, Rochereau, Kozlov, Urtecho et al., 2025) Specifically, it uses HHfilter and considers a maximum sequence identity threshold and minimum coverage with further restrictions introduced to compromise for optimal memory usage and model performance. The authors of this study use MMseqs2 for finding homologs but do not further specify the settings, which can heavily influence the search. Additionally, I believe that "if there were more than 400 homologs, we randomly selected 400" is an insufficient explanation of the reasoning for building such a database and can influence the main findings of the study. I believe that this part needs to be redone more rigorously because the main outcomes of the referenced study and this study differ significantly, but this could potentially change when redone as suggested.

2. There is a circular logic in the validation pipeline. The authors use ProteinMPNN to design sequences based on structural constraints and then use AlphaFold2 or ESMFold to validate them. Because all these models are trained on similar structural datasets, a high TM-score might simply show that they share the same training biases, rather than proving that the designed proteins will fold in real life. This should be properly reflected in the narrative. While the authors comment on this partly in the the discussion, it did not prevent them from (excessive) claims, such as “The central finding of this study is that glutamine is the most structurally dispensable canonical amino acid and that the Escherichia coli proteome can achieve its native three-dimensional folds without it” (discussion opening).

3. Based on the findings visualized in Figure 3 authors state that: “Removing Gly or Leu caused the largest structural disruptions, consistent with their high keep-scores and unique roles in backbone geometry. Removing Gln caused the least disruption, confirming its status as the most structurally dispensable amino acid.” The problems with this statement are multiple: (1) sample of 200 proteins only is too small (2) visually the difference between the displayed TMscore distributions are infinitesimal. Are the differences statistically significant? - no such metric is reported.

4. The whole pipeline of redesigning all proteins in E. coli proteome without Q is interpreted as “the glutamine-free proteome is structurally equivalent to the native”, but is it really an intrinsic feature of Q-free proteome? Based on my opinion it could just reflect the good design ability of the ProteinMPNN tool, and it relates to the problem described in the above points. As the authors clearly state in the Discussion that “An informative extension of the present work would be to repeat the proteome-wide computational analysis for isoleucine - replacing every Ile across all 4,148 proteins with ProteinMPNN-optimal alternatives and modeling the resulting sequences with AlphaFold2, exactly as we did for glutamine.”, I believe that this is not an informative future extension but a necessary validation that needs to be added into this study to support the main conclusions of the study.

5. The authors highlight that the fully glutamine-free TEM1-Q confers resistance up to 50 µg/mL ampicillin without IPTG induction and up to 600 µg/mL with 0.1 mM IPTG. While this definitively proves the enzyme is partially functional, it represents a severe loss of activity compared to wild-type TEM-1 which is known to confer resistance to thousands of µg/mL of ampicillin.

In my opinion, a similar titration assay should be done also on cells carrying the plasmid with the wild-type variant to assess how much the TEM1 activity is reduced by substituting all glutamines. Even better would be to include more robust tests of activity such as growth rate analysis in multiplicate or in vitro activity assay.

6. Supplementary Figure S13 reveals an alarming detail regarding the physical expression of TEM1-Q. The SDS-PAGE shows two distinct bands for TEM1-Q: Arrow 'a' (~33.7 kDa) and Arrow 'b' (~28.9 kDa). The caption explains that Arrow 'a' represents an unprocessed enzyme initiating at an upstream, accidental NcoI ATG site, which adds a 20-residue His-tag extension that buries the native signal peptide internally, thereby preventing translocation via the Sec machinery. This means a major fraction of the synthesized TEM1-Q is a cytosolic, improperly targeted artifact.

The authors should explain why they used the pET-28a(+) expression vector that adds His-tag at the 5’ end of the expressed gene. The His-tag is not used in any of the downstream analyses and only hinders the activity of TEM1-Q.

Even better, in my opinion, would be to reclone TEM1-Q into a vector without His-tag where only the mature, correctly processed variant would be expressed.

Minor Comments:

1. The authors might wish to include a more comprehensive set of key references on proteins previously constructed from reduced alphabets, next to the Shibue et al. 2018 Ref, as that adds great experimental support that such reductions can be compatible with structural and functional integrity. E.g.:

www.pnas.org/cgi/doi/10.1073/pnas.1219530110

doi:10.1093/molbev/msac032

https://doi.org/10.1021/jacs.1c05367

https://doi.org/10.1002/pro.4068

2. Missing linear regression equation and statistical analysis in Fig1

3. If authors did rebuild the AF2 models, why didn’t they use for TM-score comparison because I assume that models of the No-Q mutants have been generated with the same settings as the rebuilt models so the comparison will be more accurate

4. Switched color labels in Fig4 – discrepancy between captions and color legend in the plot

5. Better explanation and introduction of an “ease-score” is missing

6. Confirm that the exact model weights, random seeds, and temperature parameters (e.g., T=0.1 or T=0.3 used in ProteinMPNN) are explicitly detailed in the Methods section to facilitate complete pipeline reproducibility. Applies to almost all of the used tools.

7. Discrepancy between calculated keep-scores in Fig1 and Supp Fig S11 – the keep-score should heavily depend on conservation of the given residue at given position. How can the authors explain the discrepancy between calculated keep-scores and percentage of the conserved positions shown in S11? Explanation and visible comparison of both should be added.

8. The SDS-PAGE gel in S13b is very hard to read. I’m not sure any conclusions can be drawn from it. I think that the authors should rework the figure.

**********

**Have the authors made all data and (if applicable) computational code underlying the findings in their manuscript fully available?**

The PLOS Data policy requires authors to make all data and code underlying the findings described in their manuscript fully available without restriction, with rare exception (please refer to the Data Availability Statement in the manuscript PDF file). The data and code should be provided as part of the manuscript or its supporting information, or deposited to a public repository. For example, in addition to summary statistics, the data points behind means, medians and variance measures should be available. If there are restrictions on publicly sharing data or code —e.g. participant privacy or use of data from a third party—those must be specified.

Reviewer #1: Yes

Reviewer #2: **No:**  Authors script used for the experiments are not provided. This is necessary to check reproducibility and ease of future use of the developed pipeline for other use cases.

Reviewer #3: Yes

Reviewer #4: None

**********

PLOS authors have the option to publish the peer review history of their article (what does this mean?). If published, this will include your full peer review and any attached files.

If you choose “no”, your identity will remain anonymous but your review may still be made public.

**Do you want your identity to be public for this peer review?**  For information about this choice, including consent withdrawal, please see our Privacy Policy.

Reviewer #1: No

Reviewer #2: No

Reviewer #3: No

Reviewer #4: No

[NOTE: If reviewer comments were submitted as an attachment file, they will be attached to this email and accessible via the submission site. Please log into your account, locate the manuscript record, and check for the action link "View Attachments". If this link does not appear, there are no attachment files.]

**Figure resubmission:**

While revising your submission, we strongly recommend that you use PLOS’s NAAS tool (https://ngplosjournals.pagemajik.ai/artanalysis) to test your figure files. NAAS can convert your figure files to the TIFF file type and meet basic requirements (such as print size, resolution), or provide you with a report on issues that do not meet our requirements and that NAAS cannot fix.

After uploading your figures to PLOS’s NAAS tool - https://ngplosjournals.pagemajik.ai/artanalysis, NAAS will process the files provided and display the results in the "Uploaded Files" section of the page as the processing is complete. If the uploaded figures meet our requirements (or NAAS is able to fix the files to meet our requirements), the figure will be marked as "fixed" above. If NAAS is unable to fix the files, a red "failed" label will appear above. When NAAS has confirmed that the figure files meet our requirements, please download the file via the download option, and include these NAAS processed figure files when submitting your revised manuscript.

**Reproducibility:**

To enhance the reproducibility of your results, we recommend that authors of applicable studies deposit laboratory protocols in protocols.io, where a protocol can be assigned its own identifier (DOI) such that it can be cited independently in the future. Additionally, PLOS ONE offers an option to publish peer-reviewed clinical study protocols. Read more information on sharing protocols at https://plos.org/protocols?utm_medium=editorial-email&utm_source=authorletters&utm_campaign=protocols

# Author comment: Author response to Decision Letter 1

**DOI:** 10.1371/journal.pcbi.1014931.r003

9 Sep 2026

Attachment Submitted filename: response_to_reviewers.docx

# Peer review: Decision Letter 1

**DOI:** 10.1371/journal.pcbi.1014931.r004

**Authors:** Arne Elofsson, Arli Aditya Parikesit

26 Oct 2026

Dear Dr. Garciarrubio,

We are pleased to inform you that your manuscript 'Folding without glutamine: proteome-scale removal of a canonical amino acid.' has been provisionally accepted for publication in PLOS Computational Biology.

Before your manuscript can be formally accepted you will need to complete some formatting changes, which you will receive in a follow up email. A member of our team will be in touch with a set of requests.

Please note that your manuscript will not be scheduled for publication until you have made the required changes, so a swift response is appreciated.

IMPORTANT: The editorial review process is now complete. PLOS will only permit corrections to spelling, formatting or significant scientific errors from this point onwards. Requests for major changes, or any which affect the scientific understanding of your work, will cause delays to the publication date of your manuscript.

Should you, your institution's press office or the journal office choose to press release your paper, you will automatically be opted out of early publication. We ask that you notify us now if you or your institution is planning to press release the article. All press must be co-ordinated with PLOS.

Thank you again for supporting Open Access publishing; we are looking forward to publishing your work in PLOS Computational Biology.

Best regards,

Arli Aditya Parikesit, PhD

Academic Editor

PLOS Computational Biology

Arne Elofsson

Section Editor

PLOS Computational Biology

***********************************************************

The authors have revised the manuscript accordingly. Therefore, I decided to accept this manuscript for publication.

Reviewer's Responses to Questions

**Comments to the Authors:**

**Please note here if the review is uploaded as an attachment.**

Reviewer #2: The authors have addressed both of my comments.

Reviewer #3: The authors have successfully responded to my all questions and criticisms. The manuscript has substantially improved with the revision and I recommend publication in PLoS CB.

Reviewer #4: The authors have satisified all my comments and I thus encourage acceptance of the manuscript for publication.

**********

**Have the authors made all data and (if applicable) computational code underlying the findings in their manuscript fully available?**

The PLOS Data policy requires authors to make all data and code underlying the findings described in their manuscript fully available without restriction, with rare exception (please refer to the Data Availability Statement in the manuscript PDF file). The data and code should be provided as part of the manuscript or its supporting information, or deposited to a public repository. For example, in addition to summary statistics, the data points behind means, medians and variance measures should be available. If there are restrictions on publicly sharing data or code —e.g. participant privacy or use of data from a third party—those must be specified.

Reviewer #2: Yes

Reviewer #3: Yes

Reviewer #4: Yes

**********

PLOS authors have the option to publish the peer review history of their article (what does this mean?). If published, this will include your full peer review and any attached files.

If you choose “no”, your identity will remain anonymous but your review may still be made public.

**Do you want your identity to be public for this peer review?**  For information about this choice, including consent withdrawal, please see our Privacy Policy.

Reviewer #2: No

Reviewer #3: **Yes:**  PATRICIA FERREIRA NEVES FAISCA

Reviewer #4: No

# Editor report: Acceptance letter

**DOI:** 10.1371/journal.pcbi.1014931.r005

**Authors:** Arne Elofsson, Arli Aditya Parikesit

PCOMPBIOL-D-26-01364R1

Folding without glutamine: proteome-scale removal of a canonical amino acid.

Dear Dr Garciarrubio,

I am pleased to inform you that your manuscript has been formally accepted for publication in PLOS Computational Biology. Your manuscript is now with our production department and you will be notified of the publication date in due course.

The corresponding author will soon be receiving a typeset proof for review, to ensure errors have not been introduced during production. Please review the PDF proof of your manuscript carefully, as this is the last chance to correct any errors. Please note that major changes, or those which affect the scientific understanding of the work, will likely cause delays to the publication date of your manuscript.

Soon after your final files are uploaded, unless you have opted out, the early version of your manuscript will be published online. The date of the early version will be your article's publication date. The final article will be published to the same URL, and all versions of the paper will be accessible to readers.

For Research, Software, and Methods articles, you will receive an invoice from PLOS for your publication fee after your manuscript has reached the completed accept phase. If you receive an email requesting payment before acceptance or for any other service, this may be a phishing scheme. Learn how to identify phishing emails and protect your accounts at https://explore.plos.org/phishing.

Thank you again for supporting PLOS Computational Biology and open-access publishing. We are looking forward to publishing your work!

With kind regards,

Sharmila Kamatchi

PLOS Computational Biology | Carlyle House, Carlyle Road, Cambridge CB4 3DN | United Kingdom ploscompbiol@plos.org | Phone +44 (0) 1223-442824 | ploscompbiol.org | @PLOSCompBiol
